# Supplementary material for: Chromatin binding by HORMAD proteins regulates meiotic recombination initiation
Source: EMBO J. 2024 Feb 8;43(5):8. doi: 10.1038/s44318-024-00034-3 (PMC10907721; doi:10.1038/s44318-024-00034-3)
Supplement: Supplementary file 1 — Appendix [file 44318_2024_34_MOESM1_ESM.pdf]

Appendix for: Chromatin binding by HORMAD proteins regulates meiotic recombination initiation

Appendix Table of Contents

Page 3	Appendix Table S1
Page 4	Appendix Table S2
Page 5	Appendix Table S3
Page 6	Appendix Table S4
Page 7	Appendix Table S5
Page 8	Appendix Table S6
Page 9	Appendix Table S7
Page 10-11	Appendix Figure S1
Page 12	Appendix Figure S2
Page 13	Appendix Figure S3
Page 14-15	Appendix Figure S4
Page 16	Appendix Figure S5
Page 17	Appendix Figure S6
Page 18-19	Appendix Figure S7
Page 20-21	Appendix Figure S8
Page 22	Appendix Figure S9
Page 23-24	Appendix Figure S10

Appendix Tables

Table S1. Crystallographic data collection and refinement

**Data collection**	***Vp* Hop1 Zn SAD**
Synchrotron/Beamline	ALS 12.3.1
Date collected	May 14, 2015
Resolution (Å)	32.6 – 1.51
Wavelength (Å)	1.283
Space Group	$P2_1$
Unit Cell Dimensions (a, b, c) Å	46.40, 38.94, 69.00
Unit cell Angles (α,β,γ) °	90, 109.05, 90
I/σ (last shell)	13.9 (1.2)
[a] R_{sym} (last shell)	0.048 (0.729)
[b] R_{meas} (last shell)	0.076 (0.628)
[c] $CC_{1/2}$, last shell	0.53
Completeness (last shell) %	92.2 (61.0)
Number of reflections	219746
unique	65701
Multiplicity (last shell)	3.3 (2.0)
Number of sites	2
**Refinement**	
Resolution (Å)	32.6 – 1.55
No. of reflections	32716
working	30347
free	2369
[e] R_{work} (%)	15.47
[e] R_{free} (%)	18.08
**Structure/Stereochemistry**	
Number of atoms	3237
hydrogen	1515
solvent	210
r.m.s.d. bond lengths (Å)	0.017
r.m.s.d. bond angles (°)	1.596
Ramachandran favored/allowed	98.91%/100.0%
Poor rotamers	0.57%
MolProbity Score	1.28
Clashscore (all atoms)	5.25
[f] PDB ID	7UBA
[g] SBGrid Data Bank ID	826

[a] $R_{sym} = \sum\sum_j | I_j - \langle I \rangle | / \sum I_j$, where I_j is the intensity measurement for reflection j and $\langle I \rangle$ is the mean intensity for multiply recorded reflections.

[b] $R_{meas} = \sum_h [\sqrt{(n/(n-1))} \sum_j [I_{hj} - \langle I_h \rangle] / \sum_{hj} \langle I_h \rangle$

where I_{hj} is a single intensity measurement for reflection h, $\langle I_h \rangle$ is the average intensity measurement for multiply recorded reflections, and n is the number of observations of reflection h.

[c] $CC_{1/2}$ is the Pearson correlation coefficient between the average measured intensities of two randomly-assigned half-sets of the measurements of each unique reflection.

[e] $R_{work, free} = \sum ||F_{obs}| - |F_{calc}|| / |F_{obs}|$, where the working and free *R*-factors are calculated using the working and free reflection sets, respectively.

[f] Coordinates and structure factors have been deposited in the RCSB Protein Data Bank (http://www.rcsb.org).

[g] Diffraction data have been deposited with the SBGrid Data Bank (http://data.sbgrid.org) with the noted accession codes.

Table S2. Saccharomycetaceae Hop1 proteins used for sequence alignments

NCBI Accession #	Species
NP_012193.3	*Saccharomyces cerevisiae* S288C
EJS43276.1	*Saccharomyces arboricola* H-6
EJT42208.1	*Saccharomyces kudriavzevii* IFO 1802
XP_018221544.1	*Saccharomyces eubayanus*
XP_001642921.1	*Vanderwaltozyma polyspora* DSM 70294
XP_003667640.1	*Naumovozyma dairenensis* CBS 421
XP_003674303.1	*Naumovozyma castellii* CBS 4309
XP_003648139.1	*Eremothecium cymbalariae* DBVPG#7215
XP_017987107.1	*Eremothecium sinecaudum*
XP_452539.1	*Kluyveromyces lactis*
BAO40435.1	*Kluyveromyces marxianus* DMKU3-1042
BAP71922.1	*Kluyveromyces marxianus*
XP_002551673.1	*Lachancea thermotolerans* CBS 6340
CUS23296.1	*Lachancea quebecensis*
CEP60095.1	*Lachancea lanzarotensis*
SCU79936.1	*Lachancea* sp. CBS 6924
SCU95736.1	*Lachancea meyersii* CBS 8951
SCU82479.1	*Lachancea nothofagi* CBS 11611
SCU91559.1	*Lachancea dasiensis*
SCU92480.1	*Lachancea mirantina*
SCV99872.1	*Lachancea fermentati*
CCK69524.1	*Kazachstania naganishii* CBS 8797
XP_449898.1	*Candida glabrata*
XP_003955564.1	*Kazachstania africana* CBS 2517
XP_003688665.1	*Tetrapisispora phaffii* CBS 4417
XP_004182647.1	*Tetrapisispora blattae* CBS 6284
XP_459121.2	*Debaryomyces hansenii* CBS767
XP_002496029.1	*Zygosaccharomyces rouxii*

Table S3. Cryo-electron microscopy data collection and refinement

Data collection		
Microscope	TFS Titan Krios G3	
Voltage (keV)	300	
Nominal magnification	130,000x	
Exposure navigation	Image Shift	
Cumulative Exposure (e-/Å^2)	50.02	
Exposure rate (e-/pixel/sec)	6	
Detector	Gatan K2	
Pixel size (Å)	1.1	
GIF slit width (eV)	20	
Defocus range (µm)	-0.5 to -2	
Micrographs collected	1,314	
**Reconstruction**	**Hop1 CBR + Nucleosome**	**Nucleosome**
Final particles (no.)	139,629	302,427
B-factor (Å^2)	75.0	80.7
Resolution (Å)		
FSC 0.5 (unmasked/masked)	4.51/3.18	3.99/3.01
FSC 0.143 (unmasked/masked)	3.45/2.74	3.29/2.78
[c]Resolution range (25th/75th percentile)	2.62-4.92	2.46-4.79
**Refinement**	**Hop1 CBR + Nucleosome**	**Nucleosome**
Number of atoms	13625	12029
ligands	2 (Zn)	0
Model-Map Correlation Coefficient (masked)	0.87	0.86
Model-Map Resolution (Å)		
FSC 0.5 (unmasked/masked)	3.2/3.1	3.0/2.9
FSC 0.143 (unmasked/masked)	2.8/2.7	2.6/2.6
r.m.s.d. bond lengths (Å)	0.003	0.003
r.m.s.d. bond angles (°)	0.479	0.552
Ramachandran (%)		
Outliers	0	0
Allowed	2.65	1.87
Favored	97.35	98.13
Poor rotamers (%)	0.25	0.32
MolProbity Score	0.88	0.89
Clashscore (all atoms)	0.83	1.52
EMRinger Score	3.79	3.06
[a] PDB ID	8CWW	8CZE
[b] EMDB ID	27030	27096

[a] Coordinates have been deposited in the RCSB Protein Data Bank (http://www.rcsb.org).

[b] EM density maps (final unsharpened and sharpened maps, half maps, and masks) have been deposited to the Electron Microscopy Data Bank (https://pdbe.org/emdb).

[C] Local resolution range calculated at atom positions from final model.

Table S4. Yeast strains used in this study

Strain	Genotype	Ref.
H7797	MAT**a**/MATalpha, ho::LYS2/ho::LYS2, lys2/lys2, ura3/URA3, leu2::hisG/LEU2, his3::hisG/HIS3, trp1::hisG/TRP1	34
H8644 ("SK288c")	MAT**a**/MAT alpha, his3Δ1/HIS3, LEU/leu2Δ0, LYS/lys2Δ0, ura3Δ0/URA3, RME1(ins-308a)/RME1(ins-308a), TAO3(E1493Q)/TAO3(E1493Q), MKT1(D30G)/MKT1(D30G)	43
H9120	MATalpha/MAT**a**, ho::LYS2/ho::LYS2, lys2/lys2, leu2::hisG/leu2::hisG, HIS/his3::hisG, ura3::hisG/URA3, trp1::hisG/TRP1, hop1::LEU2/hop1::LEU2	This work
H11644	MAT**a/**MATalpha, ho::LYS2/ho::LYS2, lys2/lys2, ura3/URA3, leu2::hisG/LEU2, his3::hisG/HIS3, trp1::hisG/TRP1, hop1-loop2/hop1-loop2 (hop1-loop2 = R402A, K403A, K404A, K405A)	This work
H11757	MAT**a/**MATalpha, ho::LYS2/ho::LYS2, lys2/lys2, URA3/ura3, LEU2/leu2::hisG, his3::hisG/HIS3, trp1::hisG/TRP1, pch2Δ::kanMX, hop1-loop2/pch2Δ::kanMX, hop1-loop2	This work
H11758	MATalpha/MAT**a,** ho::LYS2/ho::LYS2, lys2/lys2, URA3/ura3, LEU2/leu2::hisG, HIS3/his3::hisG, TRP1/trp1::hisG, pch2Δ::kanMX/pch2Δ::kanMX	This work
H11276	MAT**a**/MATalpha, ho::hisG/ho::hisG, leu2::hisG/leu2::hisG, ura3(Δsma-pst::hisG)/ura3(Δsma-pst::hisG), HIS4::LEU2-(NBam;ori)/his4X::LEU2-(NgoMIV)-URA3	This work
H11688	MAT**a**/MATalpha, ho::hisG(?)/ho::hisG(?), leu2::hisG/leu2::hisG, ura3(Δsma-pst::hisG)/ura3(Δsma-pst::hisG), HIS3/his3::hisG(?), TRP1/TRP, his4X::LEU2-(NgoMIV)-URA3, hop1-loop2 HIS4::LEU2-(NBam;ori), hop1-loop2	This work
H11811	MAT**a**/MATalpha, leu2::hisG/leu2::hisG, ura3(Δsma-pst::hisG), HIS3/his3::hisG(?), TRP1/trp1::hisG, ho::hisG(?)/ho::hisG(?), ura3/ura3(Δsma-pst::hisG), his4X::LEU2-(NgoMIV)-URA3/HIS4::LEU2-(NBam;ori), hop1-loop2/hop1-loop2, pch2Δ::kanMX/pch2Δ::kanMX4	This work
H11812	MAT**a**/MATalpha, ho::hisG(?)/ho::hisG(?), leu2::hisG/leu2::hisG, ura3(Δsma-pst::hisG)/ura3, HIS3/his3::hisG(?), trp1::hisG/TRP1, his4X::LEU2-(NgoMIV)-URA3, pch2Δ::kanMX/HIS4::LEU2-(NBam;ori), pch2Δ::kanMX4	This work
H11569	MAT**a**/MATalpha, ho::hisG/ho::hisG, leu2::hisG/leu2::hisG, ura3(Δsma-pst::hisG)/ura3(Δsma-pst::hisG), HIS3/his3::hisG, trp1::hisG/TRP1, HIS4::LEU2-(NBam;ori)/HIS4::LEU2-(NBam;ori), rad50S::URA3/rad50S::URA3, hop1::kanMX/hop1::kanMX (kanMX replacing aa 71-134)	This work
H11570	MAT**a**/MATalpha, ho::hisG/ho::hisG, leu2::hisG/leu2::hisG, ura3(Δsma-pst::hisG)/ura3(Δsma-pst::hisG), his3::hisG/HIS3, trp1::hisG/TRP1 HIS4::LEU2-(NBam;ori)/HIS4::LEU2-(NBam;ori), rad50S::URA3/rad50S::URA3	This work
H11810	MAT**a**/MATalpha, ho::LYS2/ho::LYS2, lys2/lys2, ura3/ura3, TRP1/trp1::hisG, rad50S::URA3/rad50S::URA3, pch2Δ::kanMX/pch2Δ::kanMX	This work
H11809	MAT**a**/MATalpha, ho::LYS2/ho::LYS2, lys2/lys2, ura3/ura3, leu2::hisG/LEU2, hop1-loop2/hop1-loop2, rad50S::URA3/rad50S::URA3, pch2Δ::kanMX/pch2Δ::kanMX	This work
H12322	MAT**a**/MATalpha, ho::LYS2/ho::LYS2, lys2/lys2, URA3/ura3, leu2::hisG/LEU2, HIS3/his3::hisG(?), TRP1/trp1::hisG, tel1Δ::HIS3/tel1Δ::HIS3	This work
H12321	MAT**a**/MATalpha, ho::LYS2/ho::LYS2, lys2/lys2, URA3/ ura3, leu2::hisG/LEU2, his3::hisG/ his3::hisG(?), trp1::hisG/TRP1, tel1Δ::HIS3/tel1Δ::HIS3, hop1-loop2/hop1-loop2	This work

Table S5. Southern probes

Primer Name	Sequence	Probe Size (bp)	Reference
His4leu2 R (probe4)	AGATCTCCTACAATATCATTTTCTCGC	~6000	52
His4leu2 R (probe4)	ACCGGTGTTGGGCCTTTCAGTG		
gat1 fowrd	AGCTCAGTGTGCGTTATGCTTCC	1219	
gat1 rev	GACGAAATACACTAGGCAGG		
cbp2 fwd	gtgttccctcgctgtaagcaagcg	1640	
cbp2 rev	tgcccatgaagttctacctccgac		

Table S6. Antibodies used in immunofluorescence analyses

Antibody	Host	Working Dilution	Source
Zip1 (yN-16)	Goat	1:100	Santa Cruz
Hop1	Rabbit	1:500	Nancy Hollingsworth
phospho-Hop1	Rabbit	1:200	Andreas Hochwagen
Gmc2	Mouse	1:500	Amy MacQueen
Donkey anti-goat IgG w/ Cy3 conjugate (1.0 mg)	Donkey	1:1000	Jackson
Fluorescein (FITC) AffiniPure Donkey Anti Mouse IgG	Donkey	1:1000	Jackson
Fluorescein (FITC) AffiniPure Donkey Anti Rabbit IgG	Donkey	1:1000	Jackson
Anti-Rabbit-HRP	Goat	1:1000	Kindle Biosciences

Table S7. cc-Sequencing data sets

Shorthand Name	Library Name	Million Reads
rad50S_A1	RA77_11570_rad50S_6h_A1	7.76
rad50S_B1	RA78_11570_rad50S_6h_B1	7.62
rad50S_hop1-loop2_A1	RA79_11687_rad50Shop1-loop2_6h_A1	6.32
rad50S_hop1-loop2_B1	RA80_11687_rad50Shop1-loop2_6h_B1	8.04

Appendix Figure S1

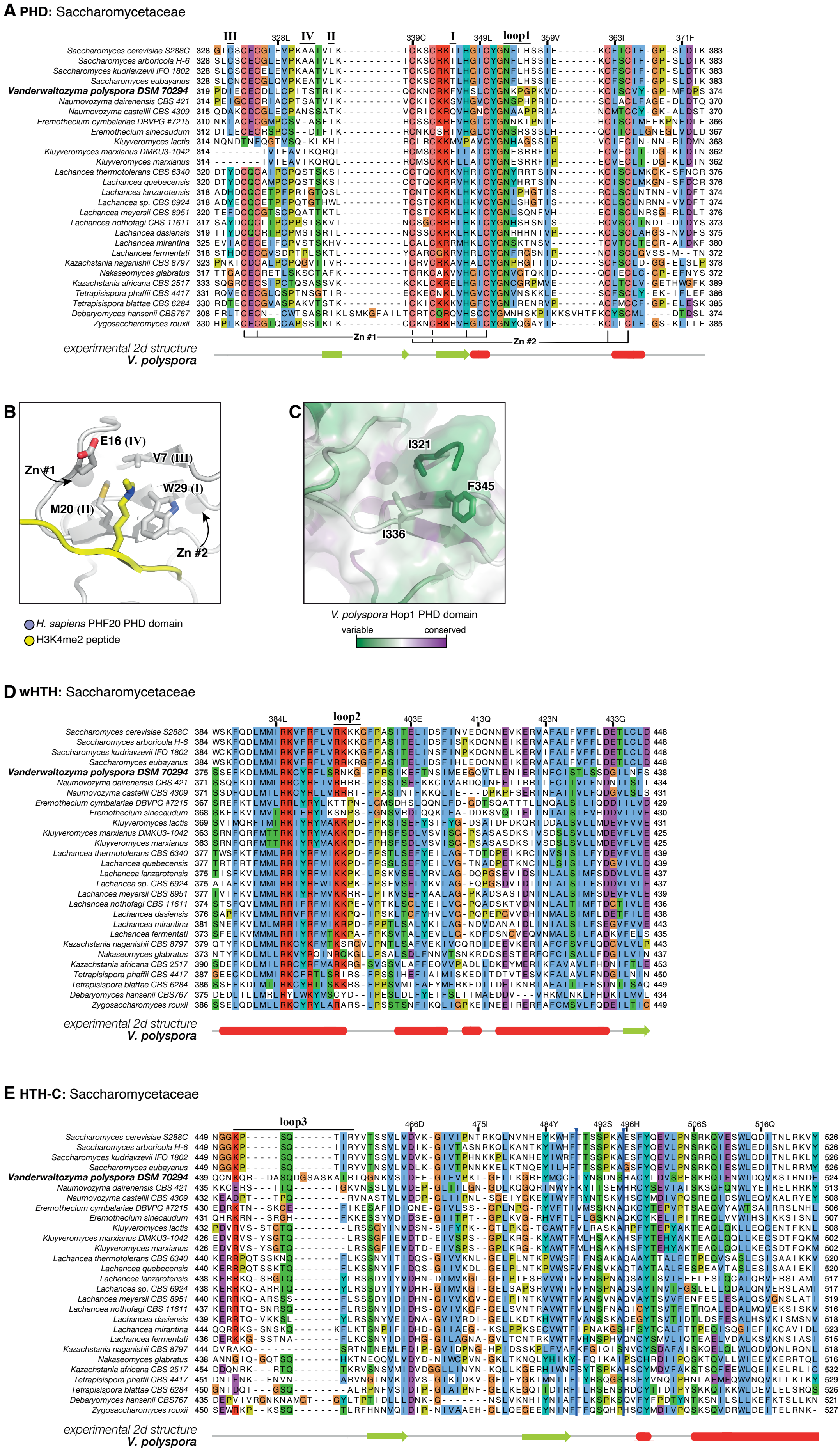

Appendix Figure S1. Structure of the Hop1 CBR PHD-wHTH-HTH-C domains from Saccharomycetaceae

(A) Sequence alignment of 28 unique Saccharomycetaceae Hop1 CBR PHD domains (NCBI accession numbers listed in Appendix Table S2), with residues coordinating zinc ions #1 and #2 noted at bottom, and the equivalent residues of the canonical PHD domain hydrophobic cage positions I-IV noted (Sanchez & Zhou, 2011). DNA binding loop 1 is noted at top. *V. polyspora* is used as a reference.

(B) Structure of the *H. sapiens* PHF20 PHD domain (white) bound to an H3K4me2 peptide (yellow) (PDB ID 5TBN) (Klein et al, 2016). Shown in sticks and labeled are PHD domain positions I-IV.

(C) Structure of the *V. polyspora* Hop1 CBR PHD domain, colored by conservation within Saccharomycetaceae (green: variable; purple: conserved) as calculated by the CONSURF server (Ashkenazy et al, 2016) from the sequence alignment in panel (A). Residues corresponding to PHD domain positions I-III are shown in sticks and labeled.

(D) Sequence alignment of 28 unique Saccharomycetaceae Hop1 CBR wHTH domains (similar to panel A), with DNA binding loop 2 is noted at top. *V. polyspora* is used as a reference.

(E) Sequence alignment of 28 unique Saccharomycetaceae Hop1 CBR HTH-C domains (similar to panel A), with DNA binding loop 3 is noted at top. *V. polyspora* is used as a reference.

Appendix Figure S2

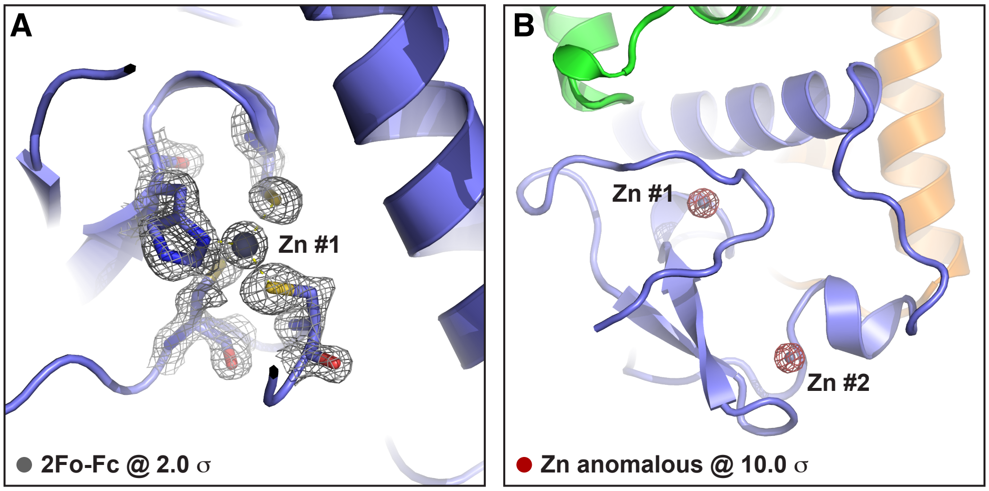

Appendix Figure S2. *V. polyspora* Hop1(317-535) electron density
(A) *2Fo-Fc* electron density at 2.0 sigma for the residues surrounding zinc site #1 in the *V. polyspora* Hop1(317-535) crystal structure.
(B) Anomalous difference electron density at 10.0 sigma for the two zinc atoms bound to *V. polyspora* Hop1(317-535).

Appendix Figure S3

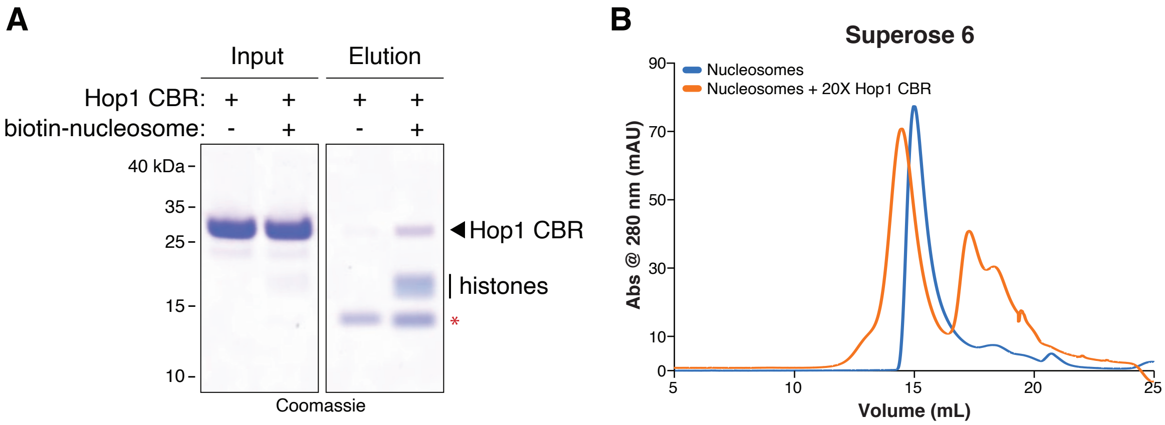

Appendix Figure S3. Assembly of a Hop1 CBR:nucleosome complex

(A) Pulldown assay with biotinylated mononucleosomes and the *S. cerevisiae* Hop1 CBR. Red asterisk indicates streptavidin eluted from the affinity resin.

(B) Superose 6 size exclusion chromatography of glutaraldehyde-crosslinked nucleosomes, either alone (blue) or pre-incubated with a 20x excess of the *S. cerevisiae* Hop1 CBR (orange).

Appendix Figure S4

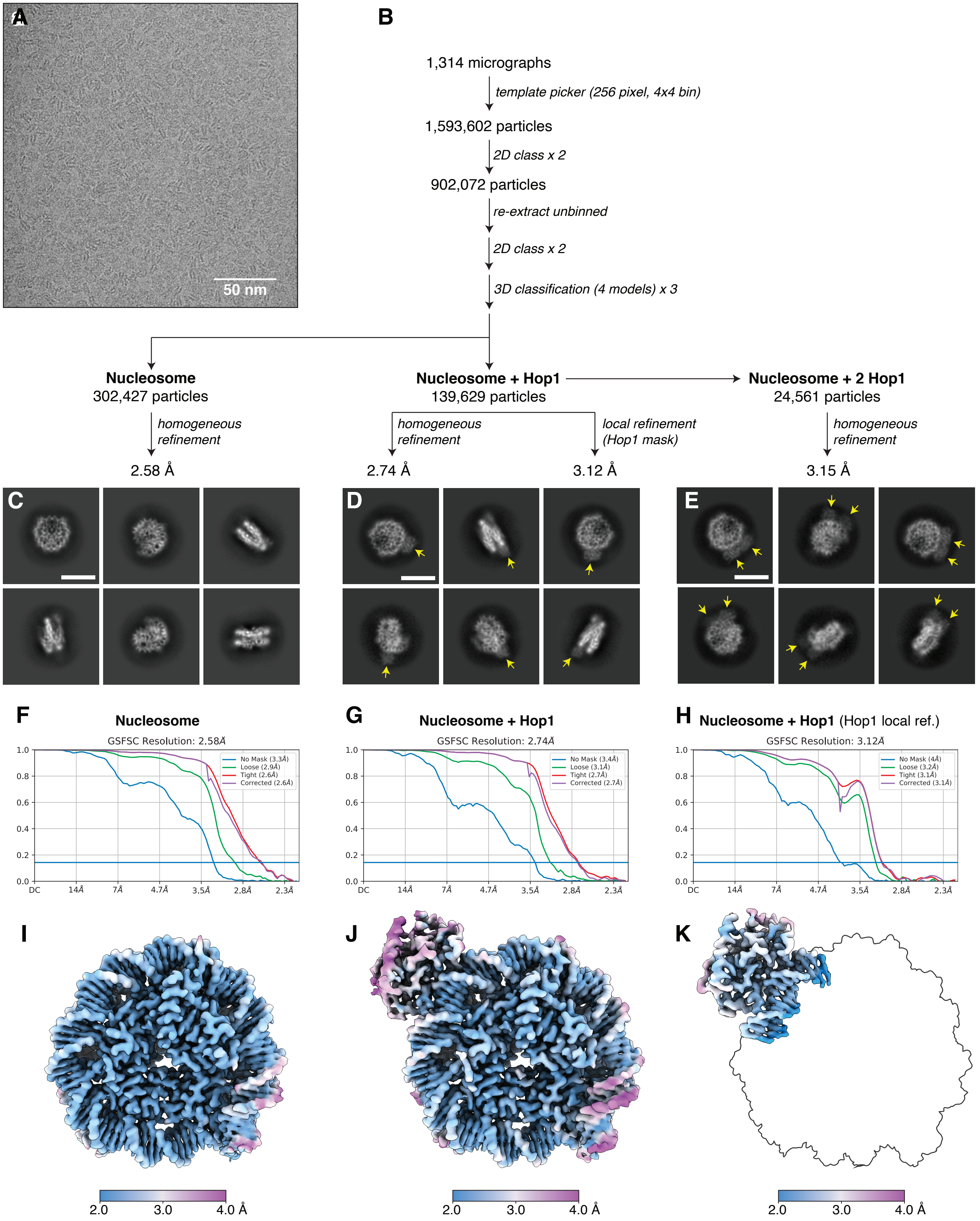

Appendix Figure S4. Cryo-EM structure of a Hop1 CBR-bound nucleosome
(A) Raw micrograph of the Hop1 CBR-nucleosome complex. Scale bar: 50 nm.
(B) Workflow for cryo-EM structure determination of a nucleosome and the Hop1 CBR:nucleosome complex.
(C-E) Selected 2D class averages for nucleosome (C), nucleosome + Hop1 CBR (D), and nucleosome + 2 Hop1 CBR (E). In panels (D) and (E), Hop1 CBR density is indicated with yellow arrows. Scale bars = 10 nm.
(F-H) Gold-standard Fourier Shell Correlation plots for refinements of nucleosome (F), nucleosome + Hop1 CBR (G), nucleosome + Hop1 CBR (local refinement of the Hop1 CBR region) (H).
(I-K) Cryo-EM maps of nucleosome (I), nucleosome + Hop1 CBR (J), nucleosome + Hop1 CBR (local refinement of the Hop1 CBR region) (K), colored by local resolution.

Appendix Figure S5

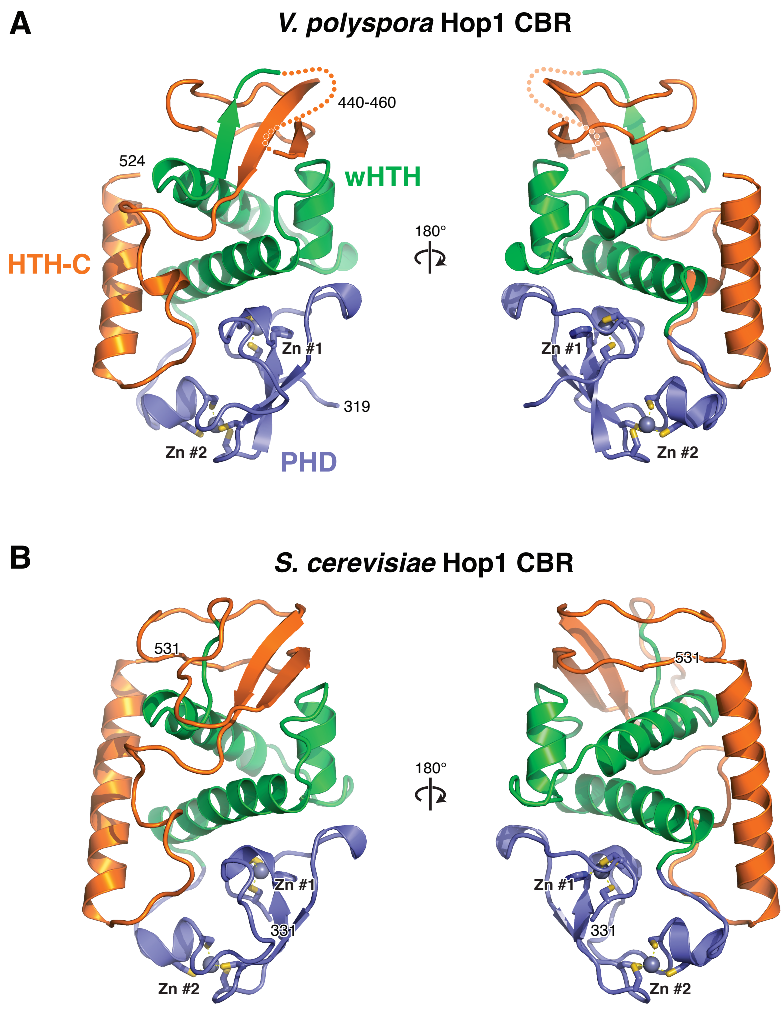

Appendix Figure S5. Hop1 CBR structure comparison
(A) Two views of the *V. polyspora* Hop1 CBR crystal structure, with PHD domain colored blue, wHTH green, and HTH-C orange. Residues 440-460 in the HTH-C region are disordered and represented as a dotted line. (B) Two views of the *S. cerevisiae* Hop1 CBR from the nucleosome-bound cryoEM structure. The two structures overlay with an overall Cα r.m.s.d. of 1.06 Å (111 residue pairs aligned).

Appendix Figure S6

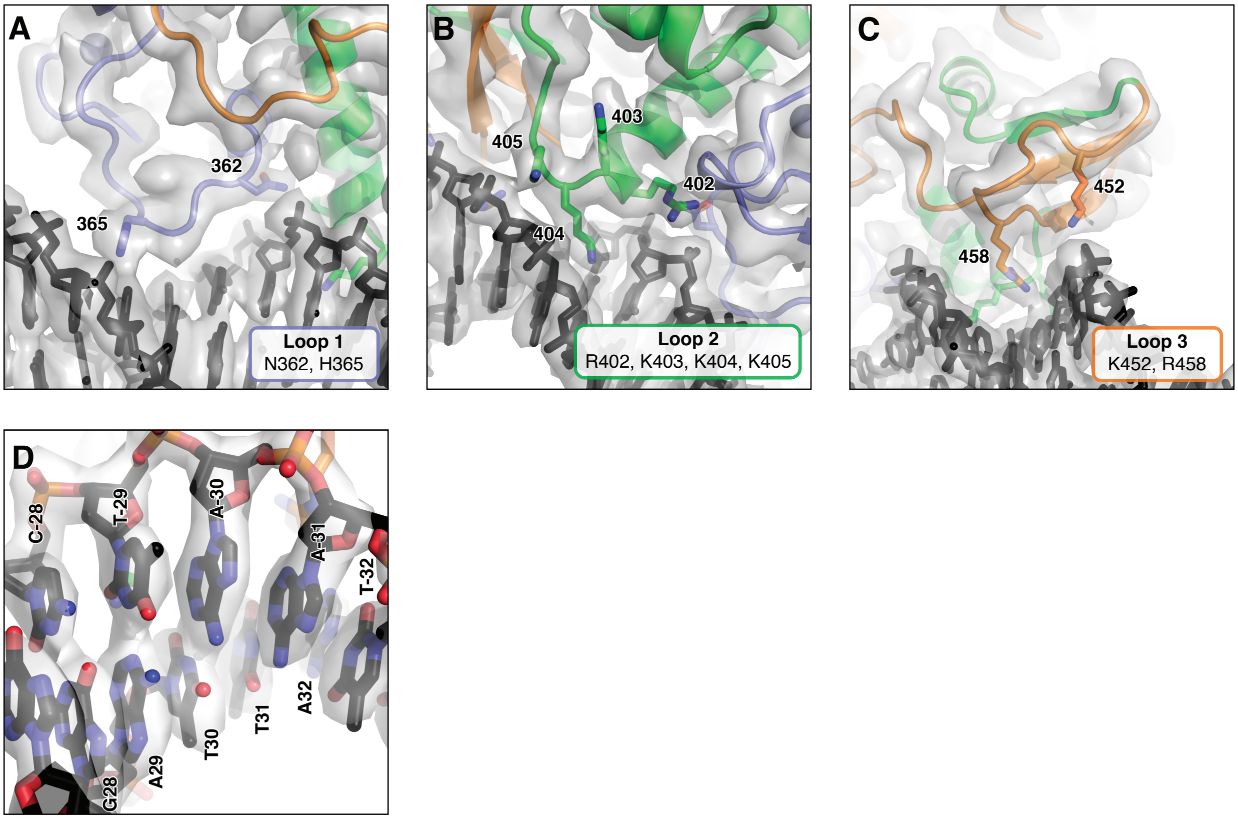

Appendix Figure S6. Hop1 CBR-DNA interactions
(A-C) CryoEM density (semi-transparent surface) at 2.74 Å resolution (unsharpened) for the Hop1 CBR Loop 1 (panel A), Loop 2 (B), and Loop 3 (C) interactions with DNA. DNA-interacting residues are labeled.
(D) CryoEM density showing a portion of nucleosomal DNA within the Hop1 CBR-bound region, showing unambiguous assignment of the orientation of the Widom 601 DNA sequence. Chain I residues -28 to -32 are at top/front, and Chain J residues 28 to 32 are at bottom/back.

Appendix Figure S7

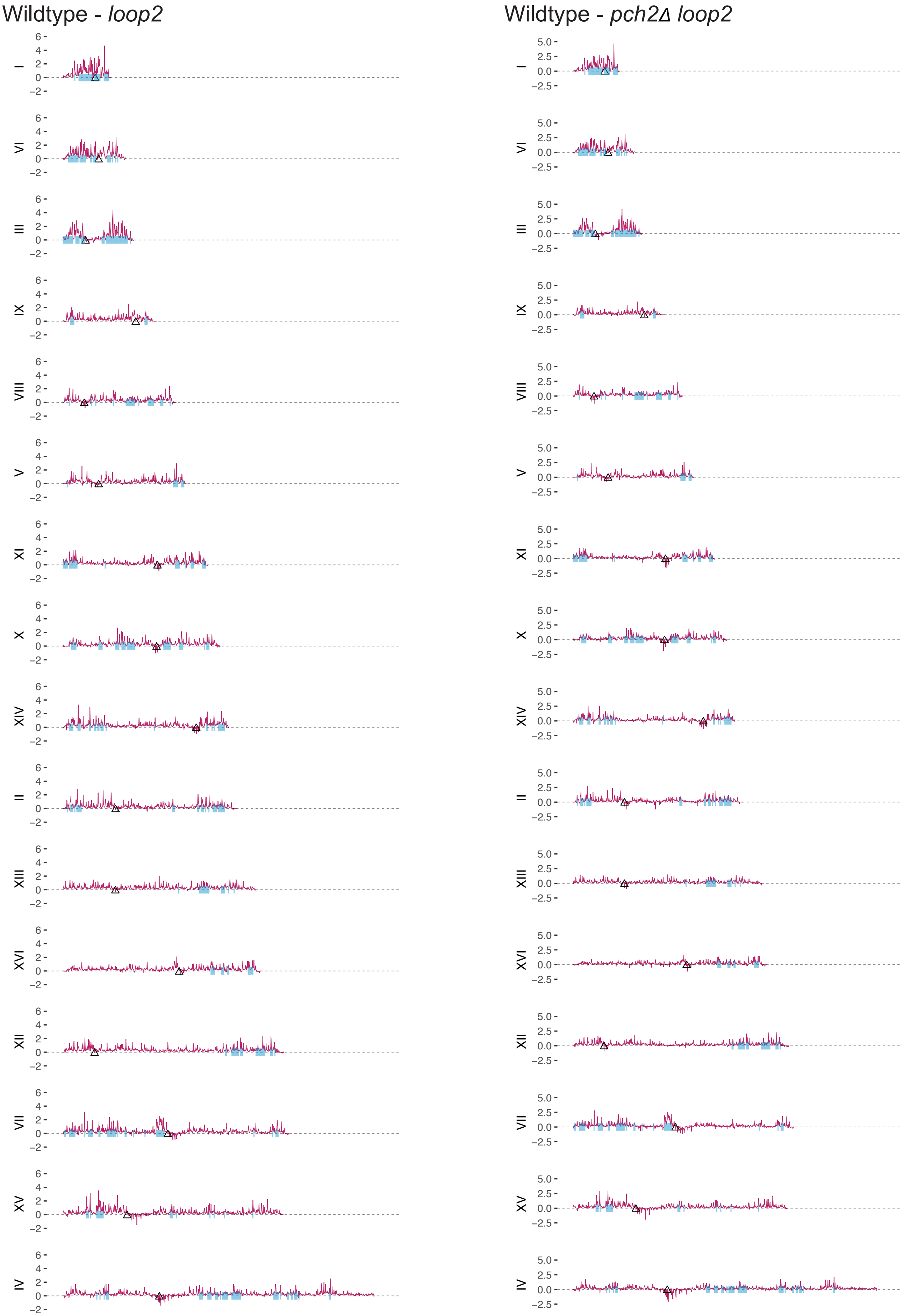

Appendix Figure S7. Whole genome plots of differential Hop1 ChIP signal

The *hop1-loop2* allele is simplified to *loop2* in figure labels. Calibrated Hop1 ChIP signal from *hop1-loop2* cells (left) or *hop1-loop2 pch2Δ* cells (right) was subtracted from wild type Hop1 ChIP signal and the remaining signal is plotted.

Appendix Figure S8

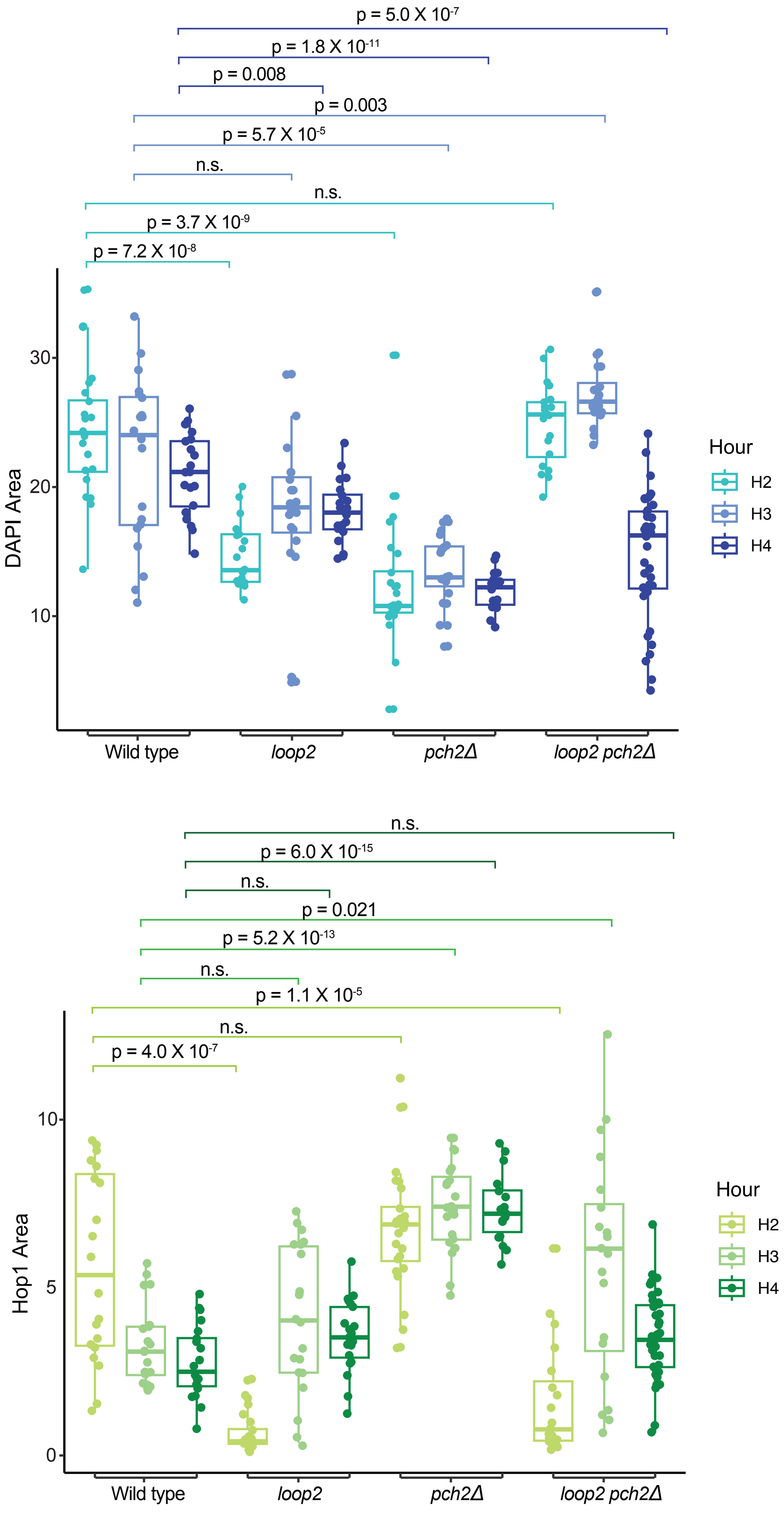

Appendix Figure S8. Hop1 and DAPI area for each genotype
The *hop1-loop2* allele is simplified to *loop2* in figure labels. (Top) Dot plot of the quantification of total DAPI area per cell. Hours 2, 3, and 4 are separated by color as depicted in the legend. Standard deviation and means are displayed for each as a box plot. Lines between categories represent p value results; n.s. is not significant. Statistics are determined by unpaired Wilcoxon test with Bonferroni correction. (Bottom) Dot plot of the quantification of total Hop1 area per cell. Graph details are the same as the top panel.

Appendix Figure S9

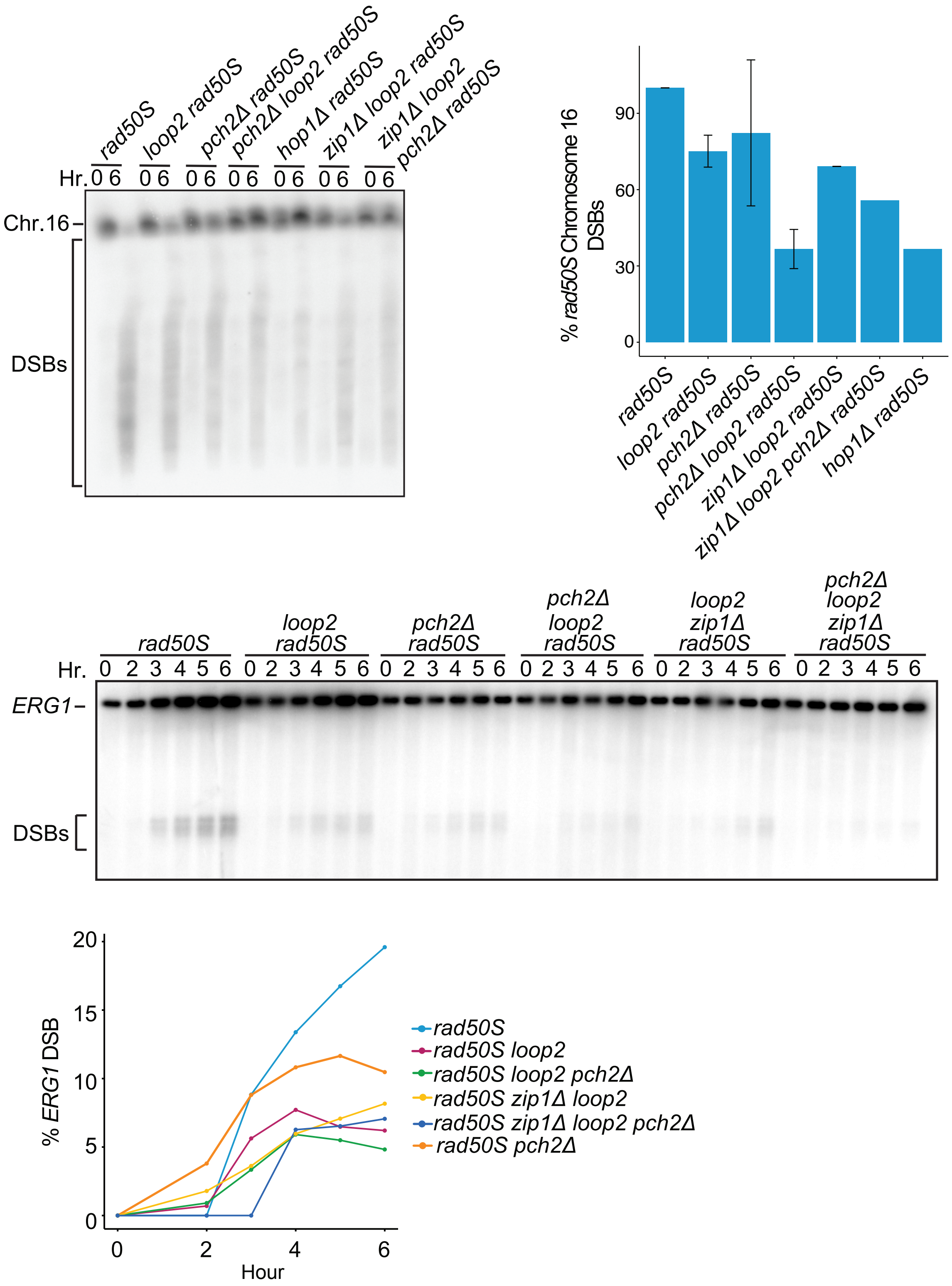

Appendix Figure S9. Supplemental Southern data

The *hop1-loop2* allele is simplified to *loop2* in figure labels. (Top Left) Pulsed-field gel and Southern blot analysis of chromosome XVI (analyzed using a probe against *SAM3*). (Top Right) Percent total broken DNA calculated, from two biological replicates, based on a Poisson distribution using the intact chromosome XVI bands for each genotype and assuming full replication by 6 hours (Thacker et al, 2014). (Middle) Southern blot analysis of the *ERG1* locus across genotypes during meiosis. Top band represents the intact *ERG1* locus. Bottom band represents DSBs that occur at the *ERG1* locus. (Bottom) Measurements of the DSB induction for each genotype at the *ERG1* locus.

Appendix Figure S10

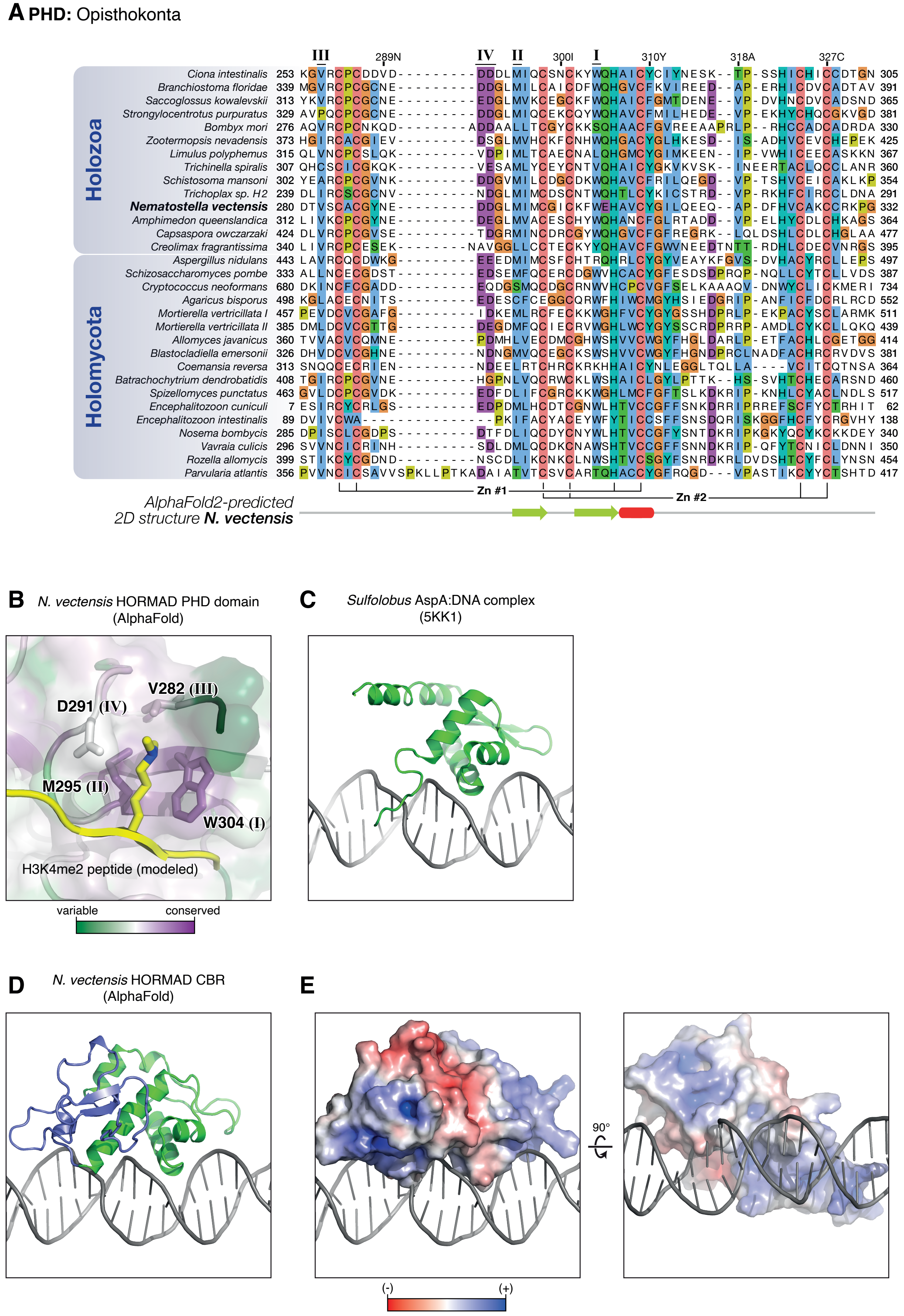

Appendix Figure S10. Meiotic HORMAD PHD domains have a conserved binding pocket
(A) Sequence alignment of the PHD domain of meiotic HORMADs found among Opisthokonta (Fungi+animals+unicellular relatives) with a PHD+wHTH domain pair (excluding HORMADs with HTH-C) shown in Figure 5A, focusing on the PHD domain. Residues coordinating zinc ions #1 and #2 noted at bottom, and the equivalent residues of the canonical PHD domain hydrophobic cage positions I-IV noted (Sanchez & Zhou, 2011).
(B) AlphaFold 2 model structure of *N. vectensis* meiotic HORMAD (Uniprot ID A7RLI6), focusing on the putative binding pocket for a lysine residue in a histone tail. Shown in yellow is an H3K4me2 peptide modeled from a structure of the *H. sapiens* PHF20 PHD domain (white) bound to an H3K4me2 peptide (PDB ID 5TBN) (Klein et al, 2016). The *N. vectensis* meiotic HORMAD PHD domain is colored by conservation, as calculated by the CONSURF server (Ashkenazy et al, 2016) from the sequence alignment in panel (a).
(C) Structure of a model wHTH domain, *Sulfolobus* sp. NOBH2 pNOB8 AspA (green) bound to DNA (gray) (PDB ID 5KK1; not published)
(D) Model of the DNA-bound *N. vectensis* HORMAD CBR, created by overlaying the *N. vectensis* HORMAD CBR (PHD blue, wHTH green) onto AspA.
(E) Two views of the DNA-bound *N. vectensis* HORMAD CBR model, shown with electrostatic surface calculated by APBS (Jurrus et al, 2018). The surface predicted to bind DNA is positively charged (blue).
