# Supplementary figures and images for: Chromatin binding by HORMAD proteins regulates meiotic recombination initiation (part 1 of 2)
Source: EMBO J. 2024 Feb 8;43(5):8. doi: 10.1038/s44318-024-00034-3 (PMC10907721; doi:10.1038/s44318-024-00034-3)

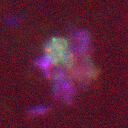

Supplement: Supplementary file 6 — Source Data Fig. 5 [file 44318_2024_34_MOESM6_ESM.zip › Data_Figure_5/2022-11-17_11758_tc/2022-10-TC_2022-11-17_11758-H4_01_R3D_RGB.tif]

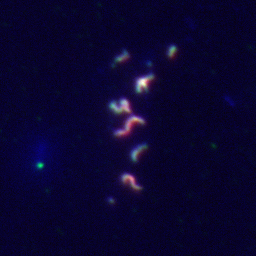

Supplement: Supplementary file 6 — Source Data Fig. 5 [file 44318_2024_34_MOESM6_ESM.zip › Data_Figure_5/2022-11-17_11758_tc/11-18-2022_cal-11758-H3-18_R3D_RGB.tif]

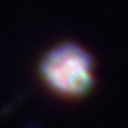

Supplement: Supplementary file 6 — Source Data Fig. 5 [file 44318_2024_34_MOESM6_ESM.zip › Data_Figure_5/2022-11-17_11758_tc/11-18-2022_cal-11758-H4-17_R3D_RGB.tif]

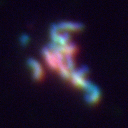

Supplement: Supplementary file 6 — Source Data Fig. 5 [file 44318_2024_34_MOESM6_ESM.zip › Data_Figure_5/2022-11-17_11758_tc/11-18-2022_cal-11758-H3-24_R3D_RGB.tif]

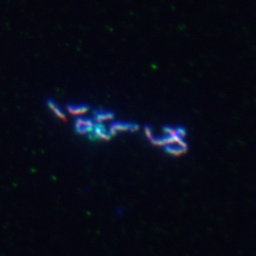

Supplement: Supplementary file 6 — Source Data Fig. 5 [file 44318_2024_34_MOESM6_ESM.zip › Data_Figure_5/2022-11-17_11758_tc/11-18-2022_cal-11758-H4-16_R3D_RGB.tif]

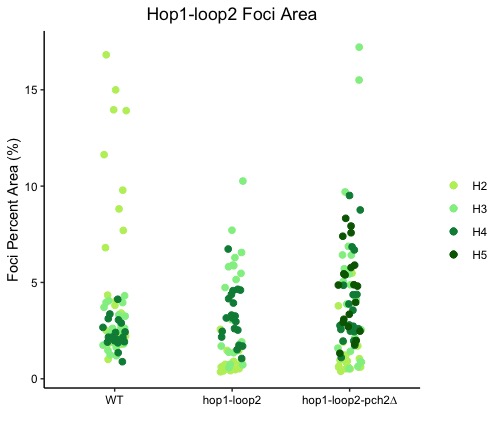

Supplement: Supplementary file 6 — Source Data Fig. 5 [file 44318_2024_34_MOESM6_ESM.zip › Data_Figure_5/Zip1TC_loop2/Hop1 Foci Area Jitter Plot 7-2022 Data.jpeg]

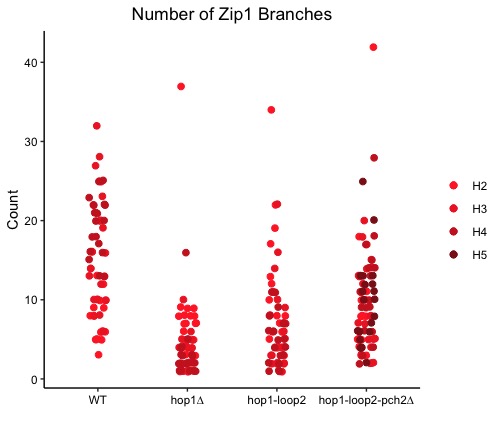

Supplement: Supplementary file 6 — Source Data Fig. 5 [file 44318_2024_34_MOESM6_ESM.zip › Data_Figure_5/Zip1TC_loop2/Number of Zip1 Branches Jitter Plot 7-2022 Data.jpeg]

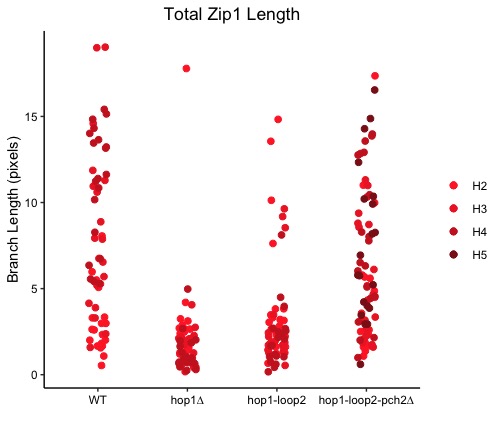

Supplement: Supplementary file 6 — Source Data Fig. 5 [file 44318_2024_34_MOESM6_ESM.zip › Data_Figure_5/Zip1TC_loop2/Total Zip1 Length Jitter Plot 7-2022 Data.jpeg]

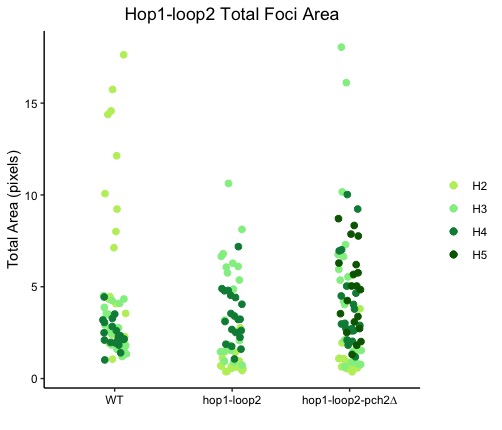

Supplement: Supplementary file 6 — Source Data Fig. 5 [file 44318_2024_34_MOESM6_ESM.zip › Data_Figure_5/Zip1TC_loop2/Hop1 Total Foci Area Jitter Plot 7-2022 Data.jpeg]

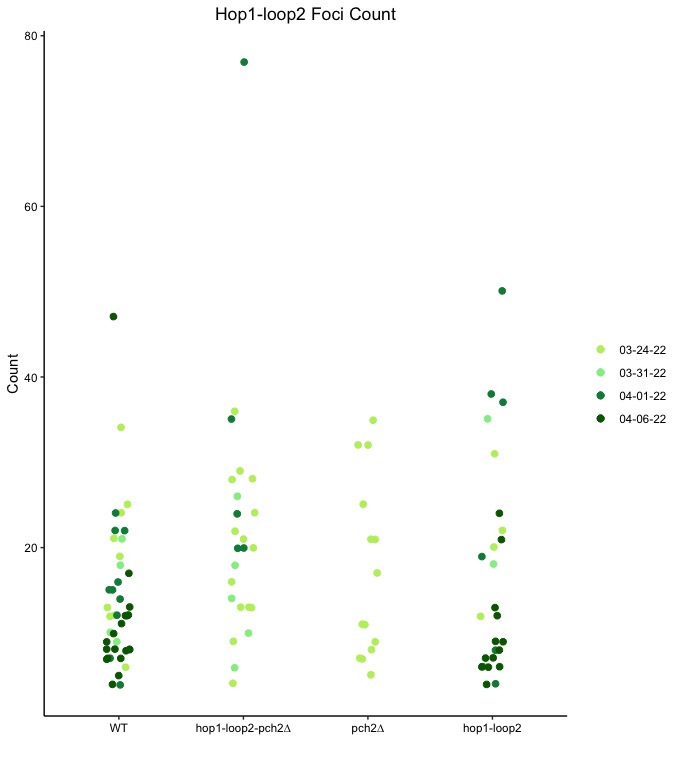

Supplement: Supplementary file 6 — Source Data Fig. 5 [file 44318_2024_34_MOESM6_ESM.zip › Data_Figure_5/hop1_loop2 images/representative images/Hop1-loop2 Foci Count compiled.jpeg]

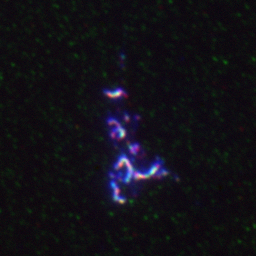

Supplement: Supplementary file 6 — Source Data Fig. 5 [file 44318_2024_34_MOESM6_ESM.zip › Data_Figure_5/hop1_loop2 images/representative images/4-1-2022_11757-hr3_07_R3D_RGB.tif]

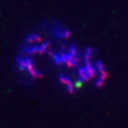

Supplement: Supplementary file 6 — Source Data Fig. 5 [file 44318_2024_34_MOESM6_ESM.zip › Data_Figure_5/hop1_loop2 images/representative images/4-1-2022_11644-hr3_02_R3D_RGB.tif]

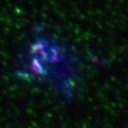

Supplement: Supplementary file 6 — Source Data Fig. 5 [file 44318_2024_34_MOESM6_ESM.zip › Data_Figure_5/hop1_loop2 images/2022-3-31/3-31-2022_11757-H3-5_09_R3D_RGB.tif]

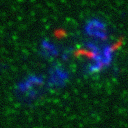

Supplement: Supplementary file 6 — Source Data Fig. 5 [file 44318_2024_34_MOESM6_ESM.zip › Data_Figure_5/hop1_loop2 images/2022-3-31/3-31-2022_11644-H3-5_02_R3D_RGB.tif]

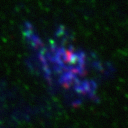

Supplement: Supplementary file 6 — Source Data Fig. 5 [file 44318_2024_34_MOESM6_ESM.zip › Data_Figure_5/hop1_loop2 images/2022-3-31/3-31-2022_7797-H3-5_09_R3D_RGB.tif]

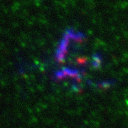

Supplement: Supplementary file 6 — Source Data Fig. 5 [file 44318_2024_34_MOESM6_ESM.zip › Data_Figure_5/hop1_loop2 images/2022-3-31/3-31-2022_11644-H3-5_07_R3D_RGB.tif]

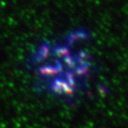

Supplement: Supplementary file 6 — Source Data Fig. 5 [file 44318_2024_34_MOESM6_ESM.zip › Data_Figure_5/hop1_loop2 images/2022-3-31/3-31-2022_11757-H3-5_06_R3D_RGB.tif]

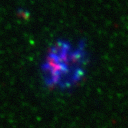

Supplement: Supplementary file 6 — Source Data Fig. 5 [file 44318_2024_34_MOESM6_ESM.zip › Data_Figure_5/hop1_loop2 images/2022-3-31/3-31-2022_11644-H3-5_08_R3D_RGB.tif]

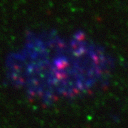

Supplement: Supplementary file 6 — Source Data Fig. 5 [file 44318_2024_34_MOESM6_ESM.zip › Data_Figure_5/hop1_loop2 images/2022-3-31/3-31-2022_11644-H3-5_03_R3D_RGB.tif]

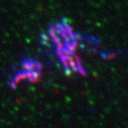

Supplement: Supplementary file 6 — Source Data Fig. 5 [file 44318_2024_34_MOESM6_ESM.zip › Data_Figure_5/hop1_loop2 images/2022-3-31/3-31-2022_7797-H3-5_07_R3D_RGB.tif]

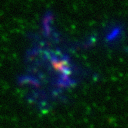

Supplement: Supplementary file 6 — Source Data Fig. 5 [file 44318_2024_34_MOESM6_ESM.zip › Data_Figure_5/hop1_loop2 images/2022-3-31/3-31-2022_11644-H3-5_10_R3D_RGB.tif]

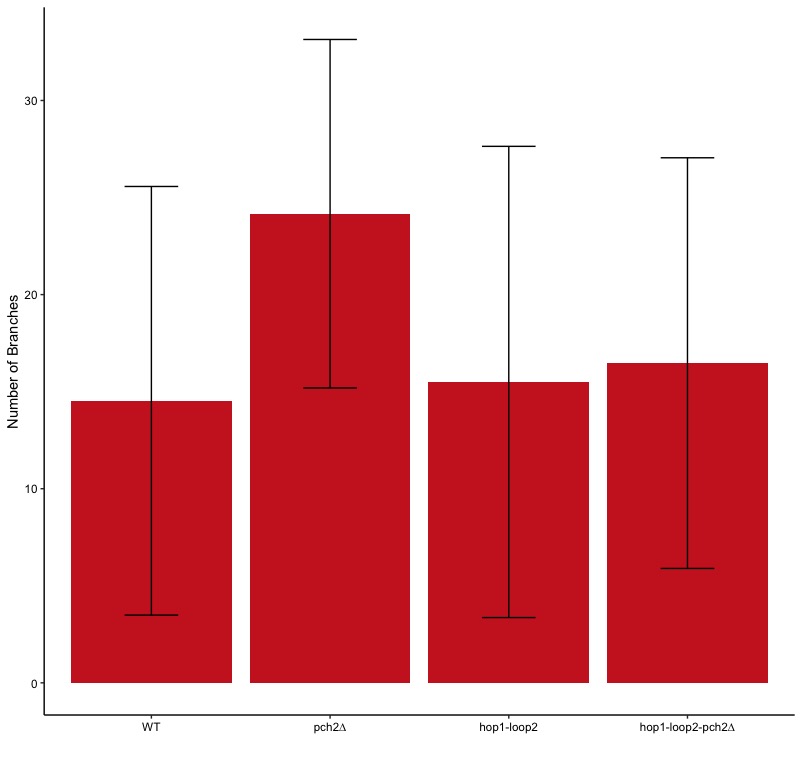

Supplement: Supplementary file 6 — Source Data Fig. 5 [file 44318_2024_34_MOESM6_ESM.zip › Data_Figure_5/hop1_loop2 images/graphs_FINAL/Number of Branches Bar Graph (all data).jpeg]

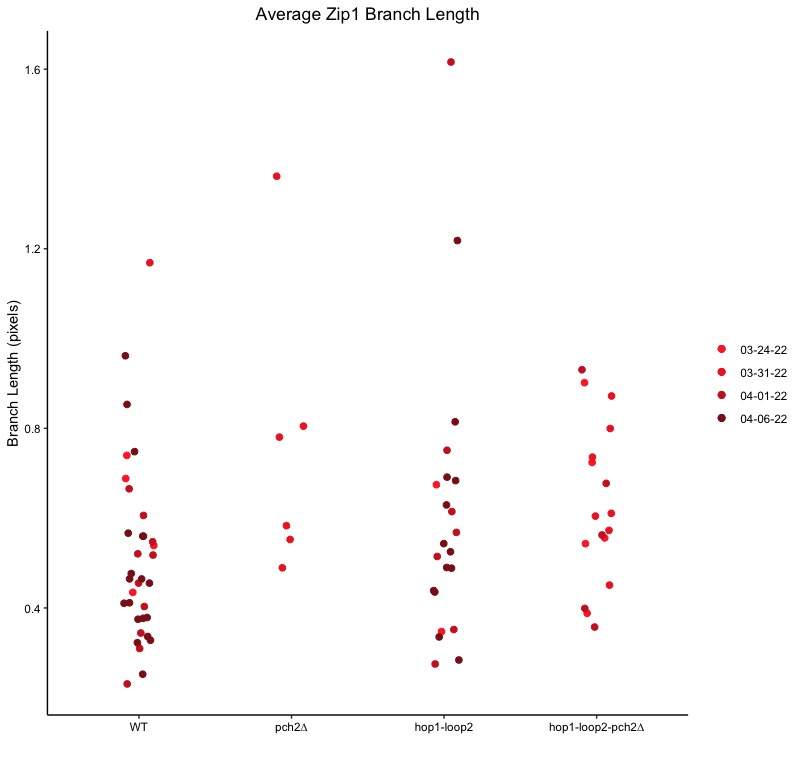

Supplement: Supplementary file 6 — Source Data Fig. 5 [file 44318_2024_34_MOESM6_ESM.zip › Data_Figure_5/hop1_loop2 images/graphs_FINAL/Average Zip1 Branch Length Jitter Plot (by DS).jpeg]

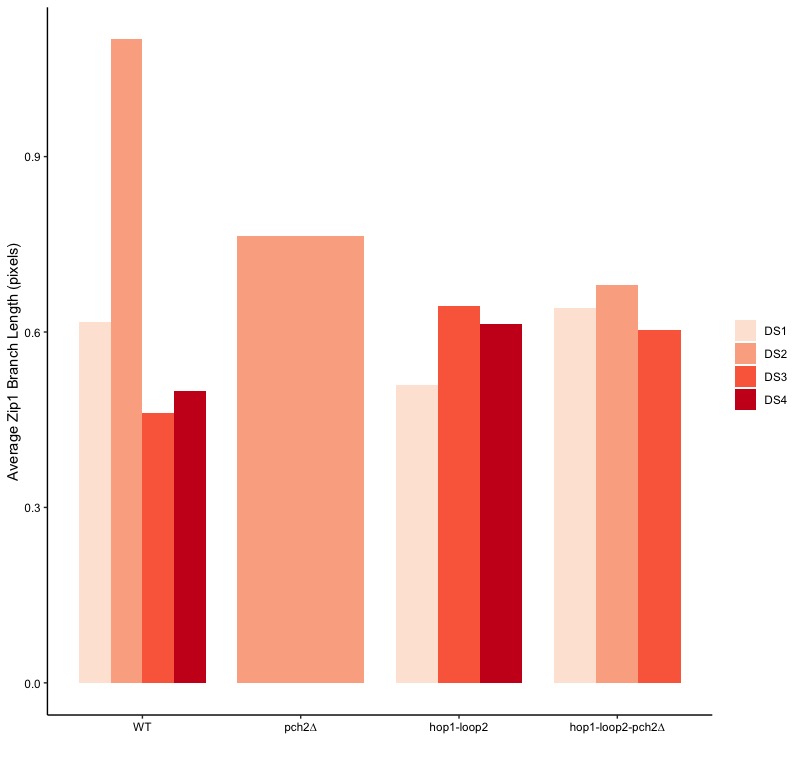

Supplement: Supplementary file 6 — Source Data Fig. 5 [file 44318_2024_34_MOESM6_ESM.zip › Data_Figure_5/hop1_loop2 images/graphs_FINAL/Average Zip1 Branch Length Bar Graph (by DS).jpeg]

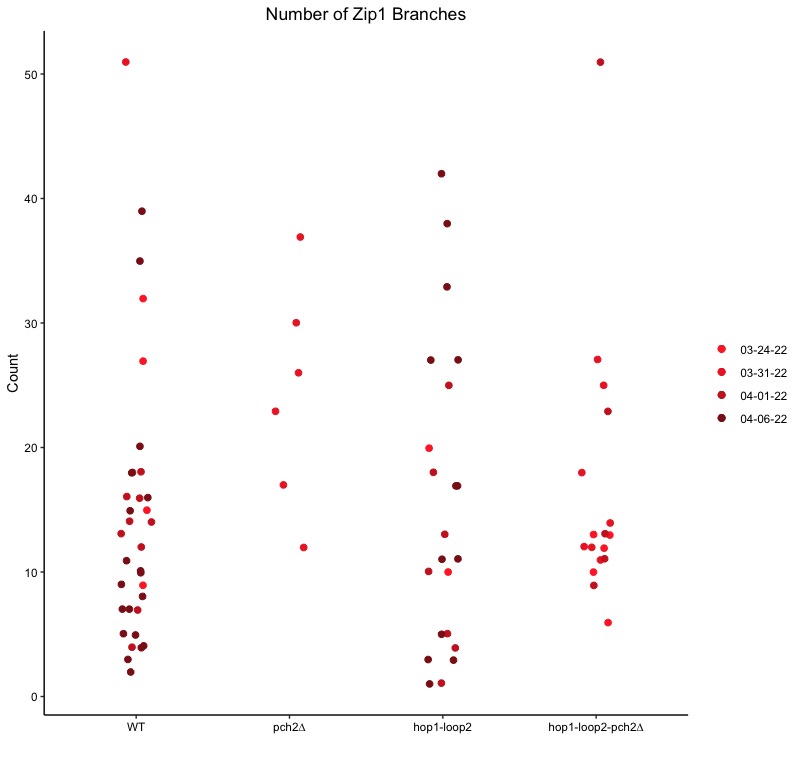

Supplement: Supplementary file 6 — Source Data Fig. 5 [file 44318_2024_34_MOESM6_ESM.zip › Data_Figure_5/hop1_loop2 images/graphs_FINAL/Number of Zip1 Branches Jitter Plot (by DS).jpeg]

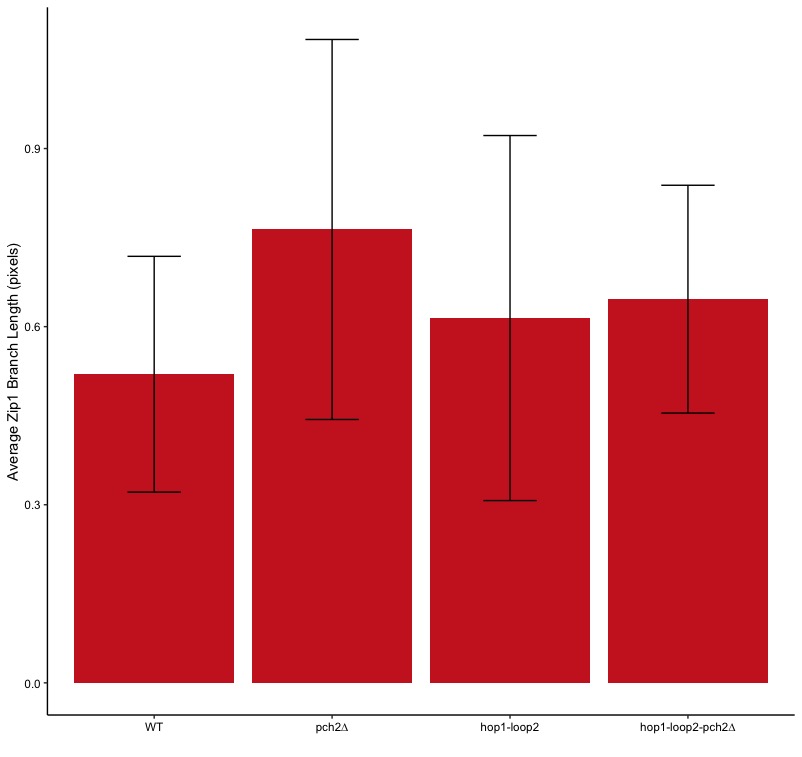

Supplement: Supplementary file 6 — Source Data Fig. 5 [file 44318_2024_34_MOESM6_ESM.zip › Data_Figure_5/hop1_loop2 images/graphs_FINAL/Average Zip1 Branch Length Bar Graph (all DS).jpeg]

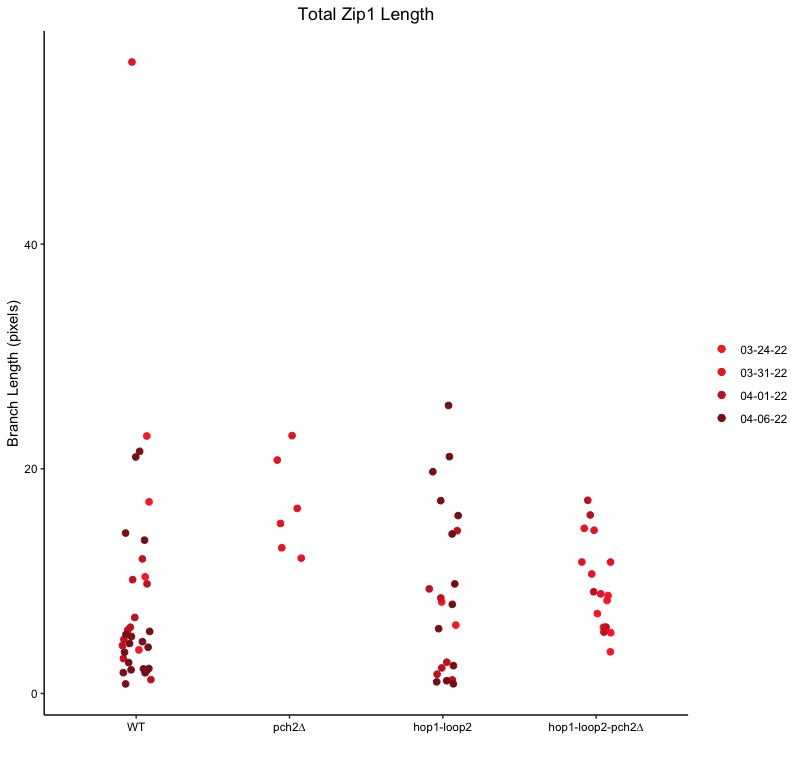

Supplement: Supplementary file 6 — Source Data Fig. 5 [file 44318_2024_34_MOESM6_ESM.zip › Data_Figure_5/hop1_loop2 images/graphs_FINAL/Total Zip1 Length Jitter Plot (by DS).jpeg]

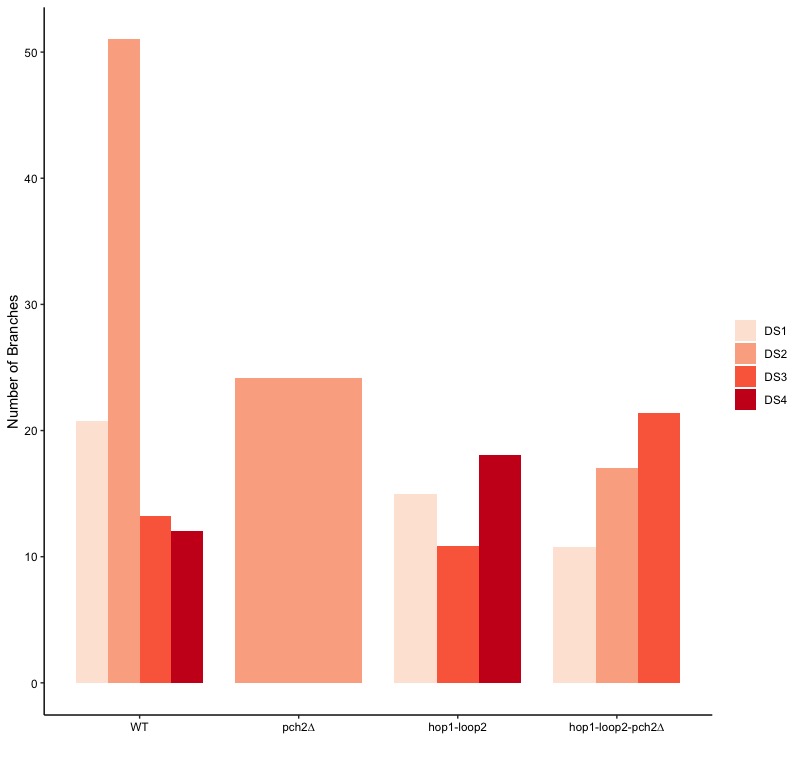

Supplement: Supplementary file 6 — Source Data Fig. 5 [file 44318_2024_34_MOESM6_ESM.zip › Data_Figure_5/hop1_loop2 images/graphs_FINAL/Number of Branches Bar Graph (by DS).jpeg]

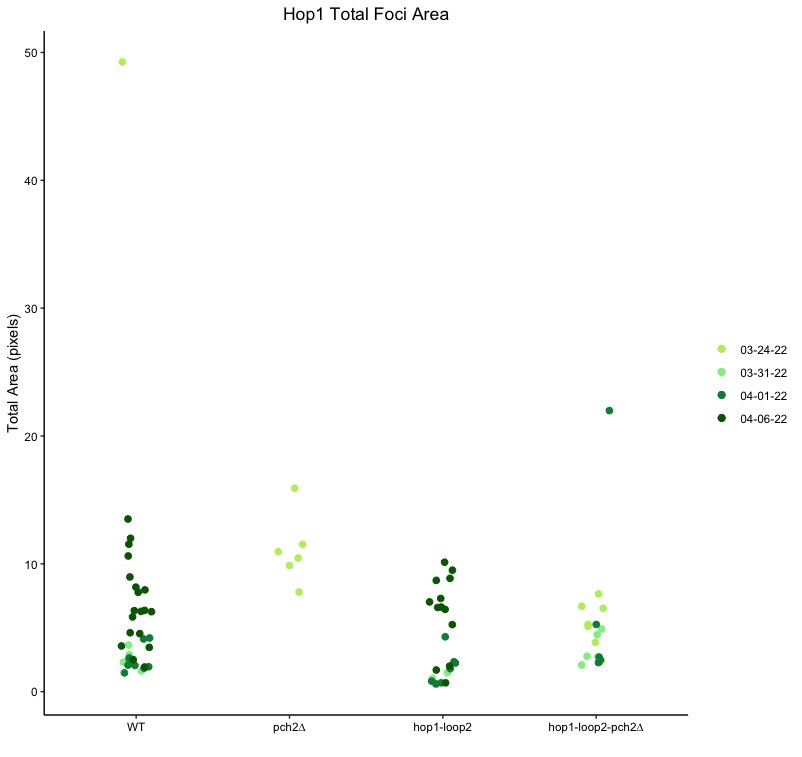

Supplement: Supplementary file 6 — Source Data Fig. 5 [file 44318_2024_34_MOESM6_ESM.zip › Data_Figure_5/hop1_loop2 images/graphs_FINAL/Hop1 Total Foci Area Jitter Plot (by DS).jpeg]

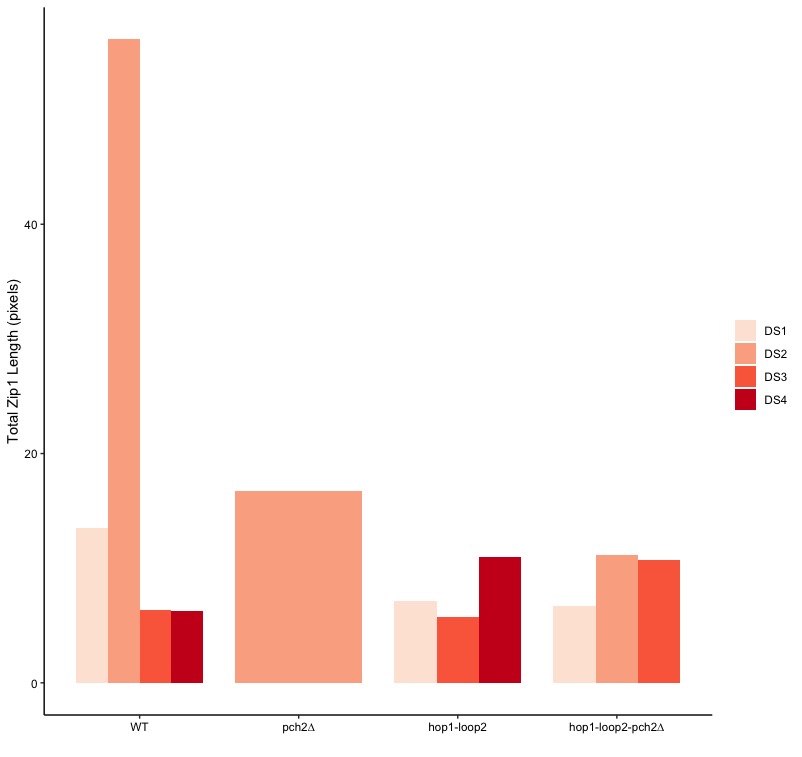

Supplement: Supplementary file 6 — Source Data Fig. 5 [file 44318_2024_34_MOESM6_ESM.zip › Data_Figure_5/hop1_loop2 images/graphs_FINAL/Total Zip1 Length Bar Graph (by DS).jpeg]

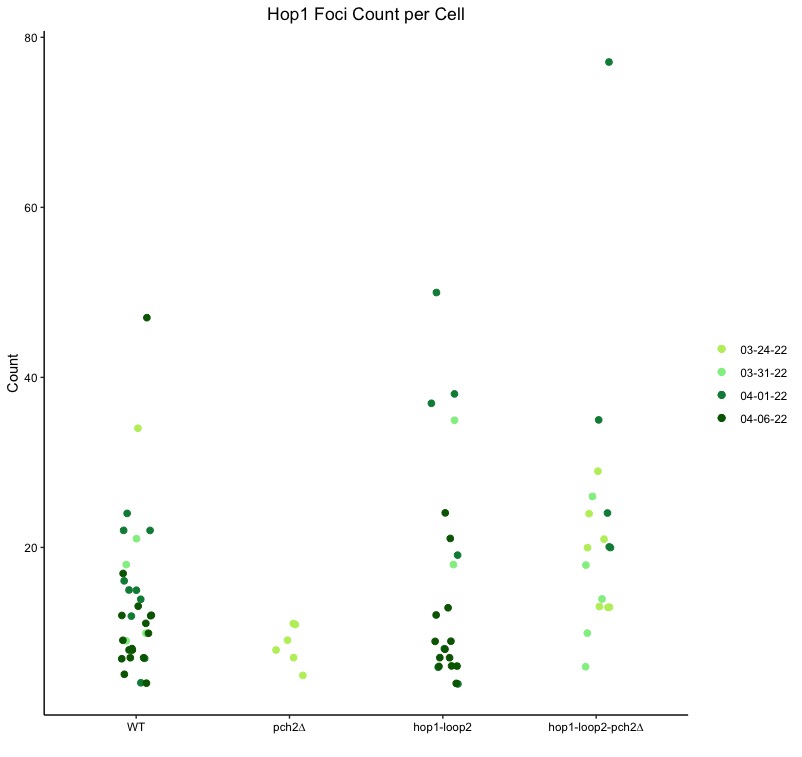

Supplement: Supplementary file 6 — Source Data Fig. 5 [file 44318_2024_34_MOESM6_ESM.zip › Data_Figure_5/hop1_loop2 images/graphs_FINAL/Hop1 Foci Count Jitter Plot (by DS).jpeg]

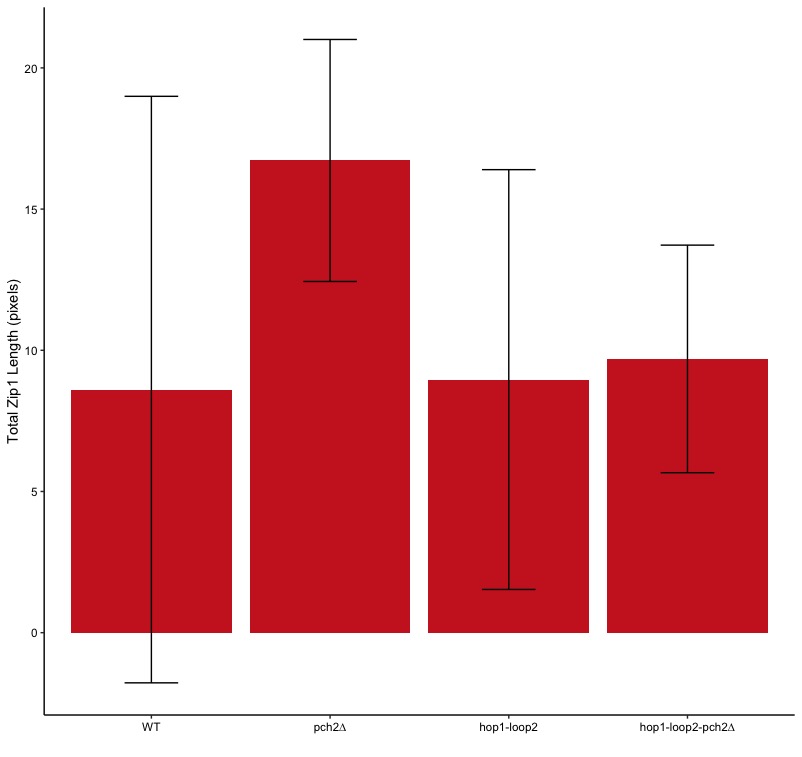

Supplement: Supplementary file 6 — Source Data Fig. 5 [file 44318_2024_34_MOESM6_ESM.zip › Data_Figure_5/hop1_loop2 images/graphs_FINAL/Total Zip1 Length Bar Graph (all data).jpeg]

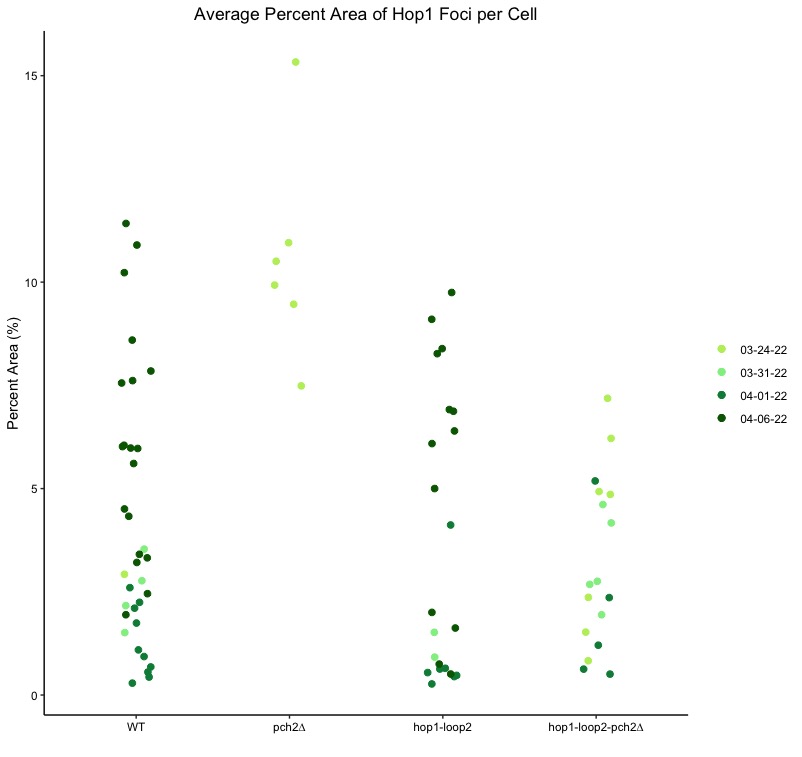

Supplement: Supplementary file 6 — Source Data Fig. 5 [file 44318_2024_34_MOESM6_ESM.zip › Data_Figure_5/hop1_loop2 images/graphs_FINAL/Average Percent Area of Hop1 Foci Jitter Plot (by DS).jpeg]

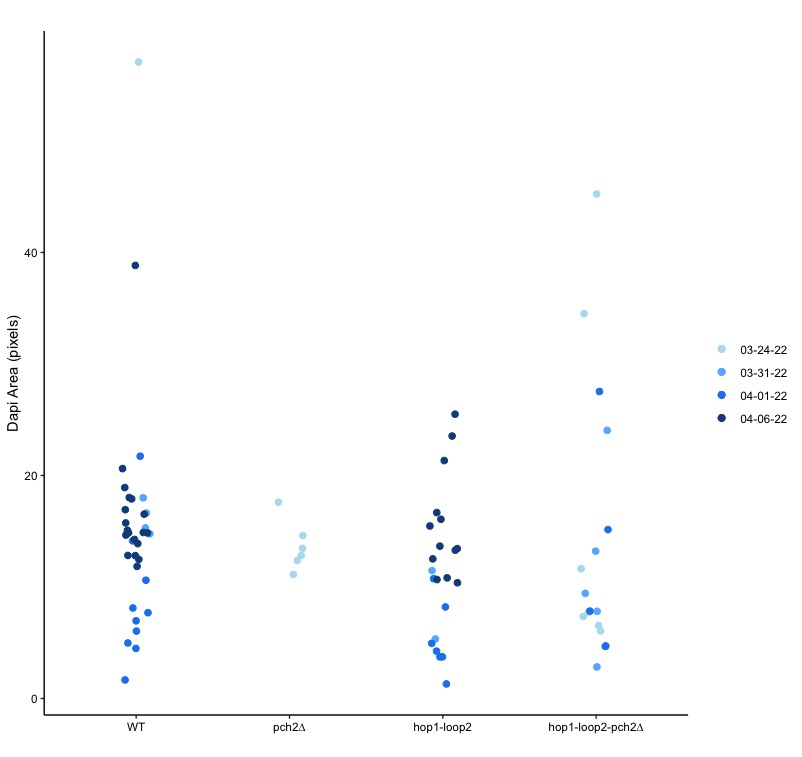

Supplement: Supplementary file 6 — Source Data Fig. 5 [file 44318_2024_34_MOESM6_ESM.zip › Data_Figure_5/hop1_loop2 images/graphs_FINAL/Dapi Area Jitter Plot (by DS).jpeg]

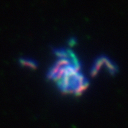

Supplement: Supplementary file 6 — Source Data Fig. 5 [file 44318_2024_34_MOESM6_ESM.zip › Data_Figure_5/2022-11-17_11758_tc/RGB/11-18-2022_cal-11758-H4-14_R3D_RGB.tif]

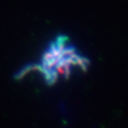

Supplement: Supplementary file 6 — Source Data Fig. 5 [file 44318_2024_34_MOESM6_ESM.zip › Data_Figure_5/2022-11-17_11758_tc/RGB/11-18-2022_cal-11758-H4-08_R3D_RGB.tif]

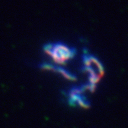

Supplement: Supplementary file 6 — Source Data Fig. 5 [file 44318_2024_34_MOESM6_ESM.zip › Data_Figure_5/2022-11-17_11758_tc/RGB/11-18-2022_cal-11758-H4-11_R3D_RGB.tif]

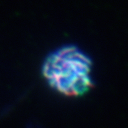

Supplement: Supplementary file 6 — Source Data Fig. 5 [file 44318_2024_34_MOESM6_ESM.zip › Data_Figure_5/2022-11-17_11758_tc/RGB/11-18-2022_cal-11758-H4-18_R3D_RGB.tif]

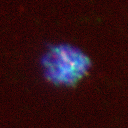

Supplement: Supplementary file 6 — Source Data Fig. 5 [file 44318_2024_34_MOESM6_ESM.zip › Data_Figure_5/2022-11-17_11758_tc/RGB/2022-10-TC_2022-11-17_11758-H2_01_R3D_RGB.tif]

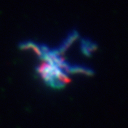

Supplement: Supplementary file 6 — Source Data Fig. 5 [file 44318_2024_34_MOESM6_ESM.zip › Data_Figure_5/2022-11-17_11758_tc/RGB/11-18-2022_cal-11758-H4-07_R3D_RGB.tif]

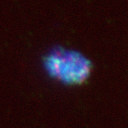

Supplement: Supplementary file 6 — Source Data Fig. 5 [file 44318_2024_34_MOESM6_ESM.zip › Data_Figure_5/2022-11-17_11758_tc/RGB/2022-10-TC_2022-11-17_11758-H2_02_R3D_RGB.tif]

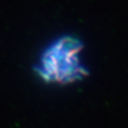

Supplement: Supplementary file 6 — Source Data Fig. 5 [file 44318_2024_34_MOESM6_ESM.zip › Data_Figure_5/2022-11-17_11758_tc/RGB/11-18-2022_cal-11758-H4-20_R3D_RGB.tif]

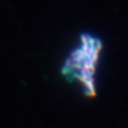

Supplement: Supplementary file 6 — Source Data Fig. 5 [file 44318_2024_34_MOESM6_ESM.zip › Data_Figure_5/2022-11-17_11758_tc/RGB/11-18-2022_cal-11758-H4-12_R3D_RGB.tif]

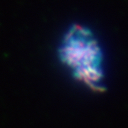

Supplement: Supplementary file 6 — Source Data Fig. 5 [file 44318_2024_34_MOESM6_ESM.zip › Data_Figure_5/2022-11-17_11758_tc/RGB/11-18-2022_cal-11758-H4-05_R3D_RGB.tif]

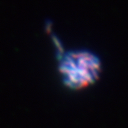

Supplement: Supplementary file 6 — Source Data Fig. 5 [file 44318_2024_34_MOESM6_ESM.zip › Data_Figure_5/2022-11-17_11758_tc/RGB/11-18-2022_cal-11758-H4-19_R3D_RGB.tif]

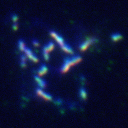

Supplement: Supplementary file 6 — Source Data Fig. 5 [file 44318_2024_34_MOESM6_ESM.zip › Data_Figure_5/2022-11-17_11758_tc/RGB/11-18-2022_cal-11758-H4-10_R3D_RGB.tif]

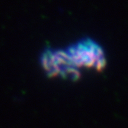

Supplement: Supplementary file 6 — Source Data Fig. 5 [file 44318_2024_34_MOESM6_ESM.zip › Data_Figure_5/2022-11-17_11758_tc/RGB/11-18-2022_cal-11758-H4-09_R3D_RGB.tif]

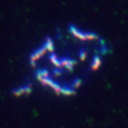

Supplement: Supplementary file 6 — Source Data Fig. 5 [file 44318_2024_34_MOESM6_ESM.zip › Data_Figure_5/2022-11-17_11758_tc/RGB/11-18-2022_cal-11758-H4-15_R3D_RGB.tif]

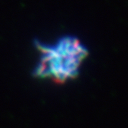

Supplement: Supplementary file 6 — Source Data Fig. 5 [file 44318_2024_34_MOESM6_ESM.zip › Data_Figure_5/2022-11-17_11758_tc/RGB/11-18-2022_cal-11758-H4-03_R3D_RGB.tif]

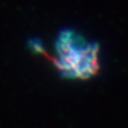

Supplement: Supplementary file 6 — Source Data Fig. 5 [file 44318_2024_34_MOESM6_ESM.zip › Data_Figure_5/2022-11-17_11758_tc/RGB/11-18-2022_cal-11758-H4-13_R3D_RGB.tif]

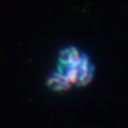

Supplement: Supplementary file 6 — Source Data Fig. 5 [file 44318_2024_34_MOESM6_ESM.zip › Data_Figure_5/2022-11-17_11758_tc/RGB/11-18-2022_cal-11758-H4-06_R3D_RGB.tif]

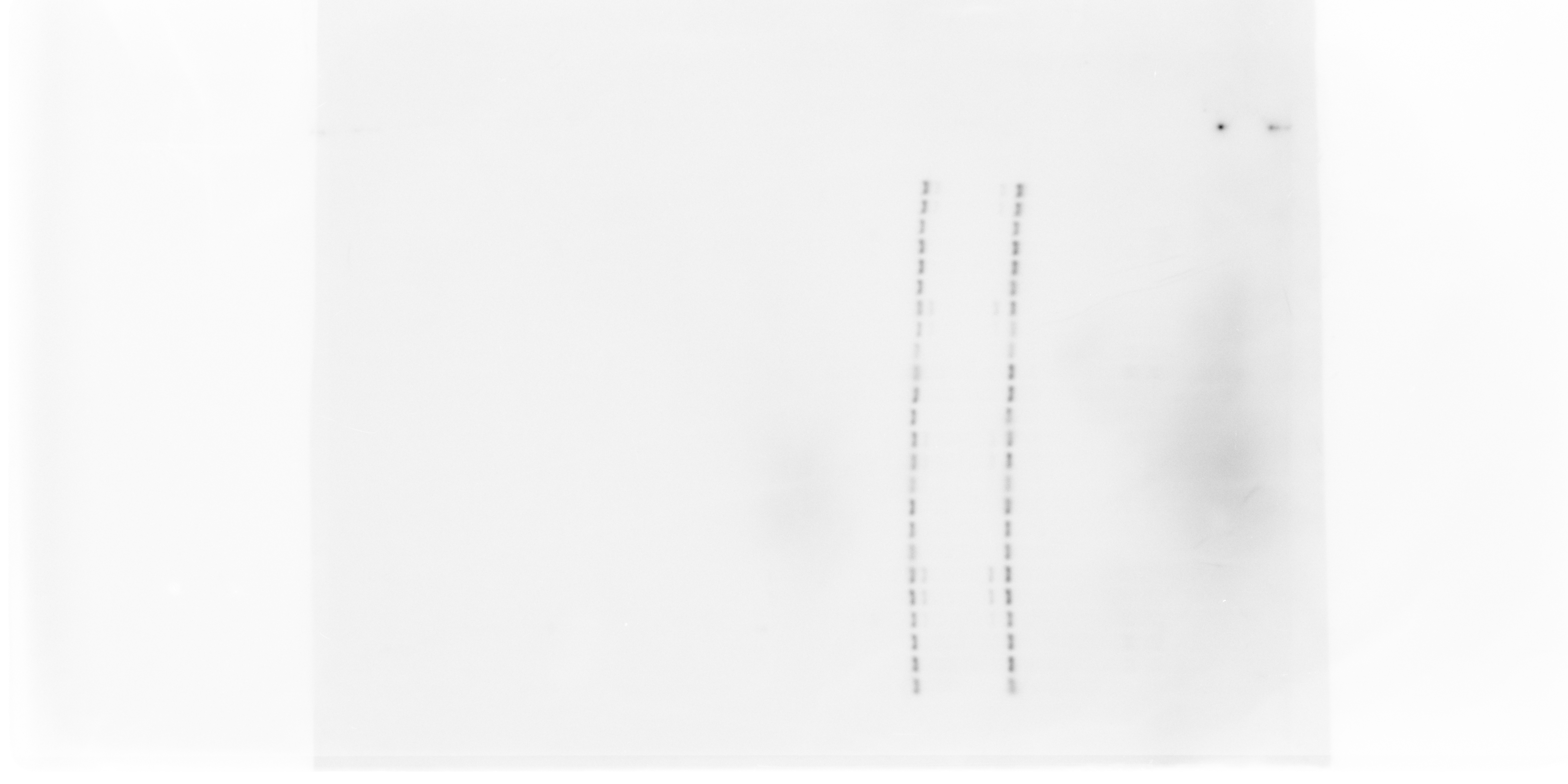

Supplement: Supplementary file 8 — Source Data Fig. 8A [file 44318_2024_34_MOESM8_ESM.zip › Figure8_PanelA/20231003-152325-1d-loop2-92923-again-[Phosphor].tif]

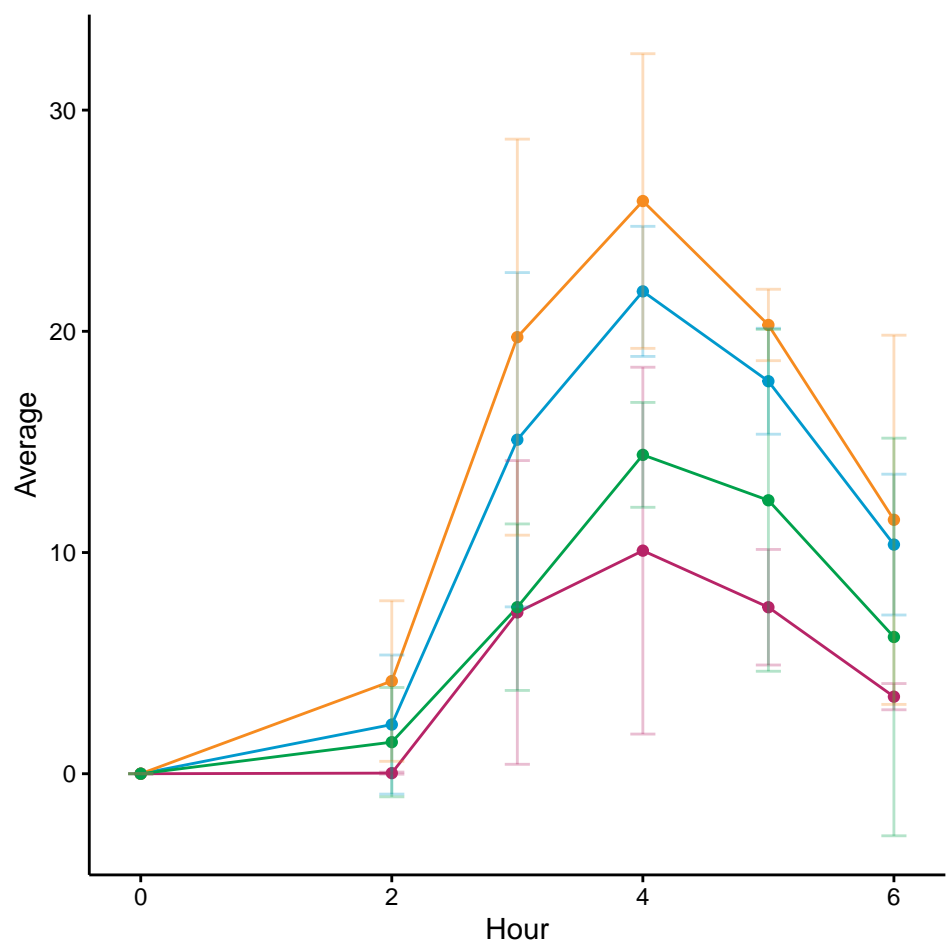

Supplement: Supplementary file 8 — Source Data Fig. 8A [file 44318_2024_34_MOESM8_ESM.zip › Figure8_PanelA/HIS4LEU2-1D-DSB_nolegend.pdf]

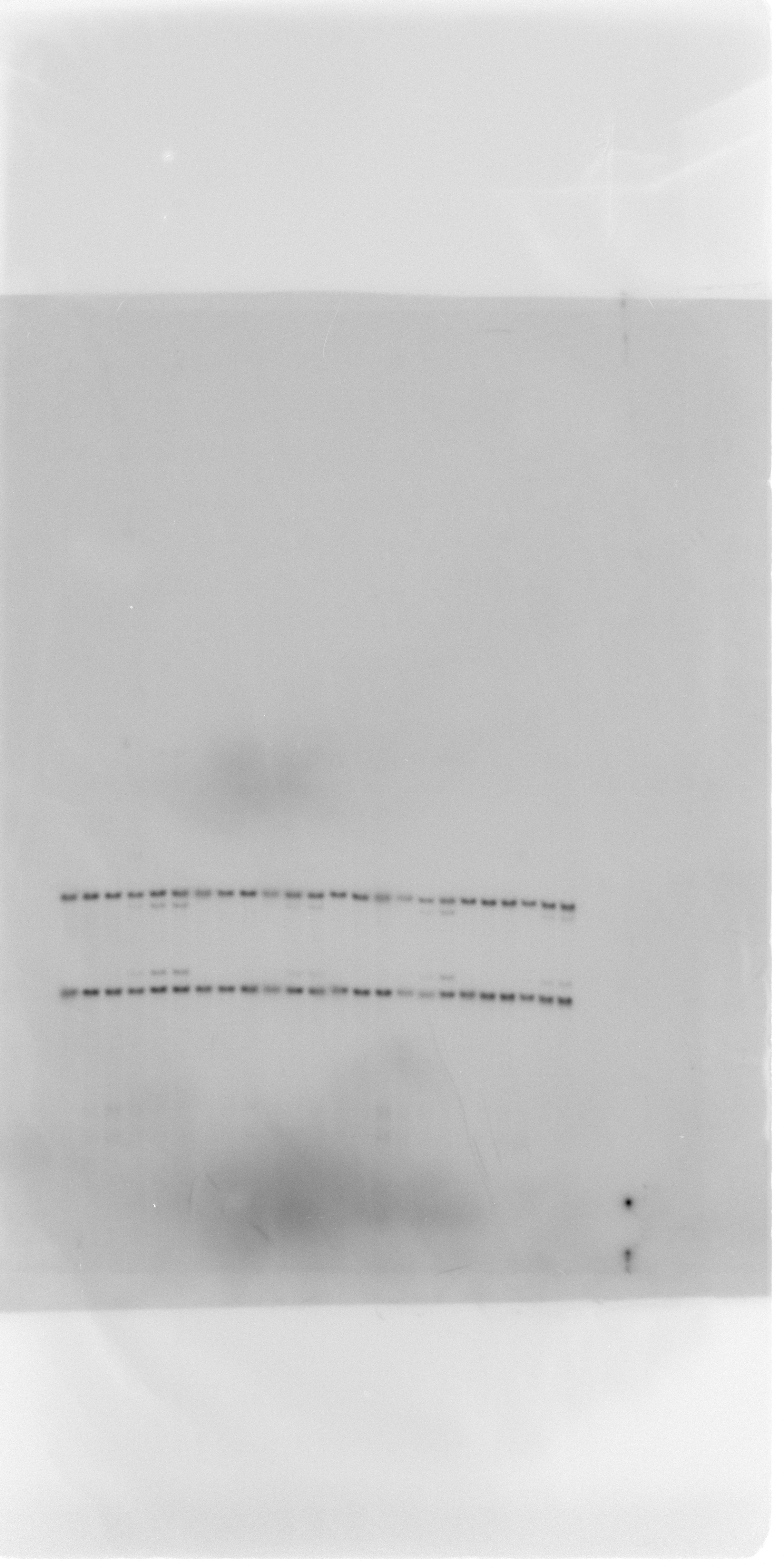

Supplement: Supplementary file 8 — Source Data Fig. 8A [file 44318_2024_34_MOESM8_ESM.zip › Figure8_PanelA/20231003-152325-1d-loop2-92923-[Phosphor].bmp]

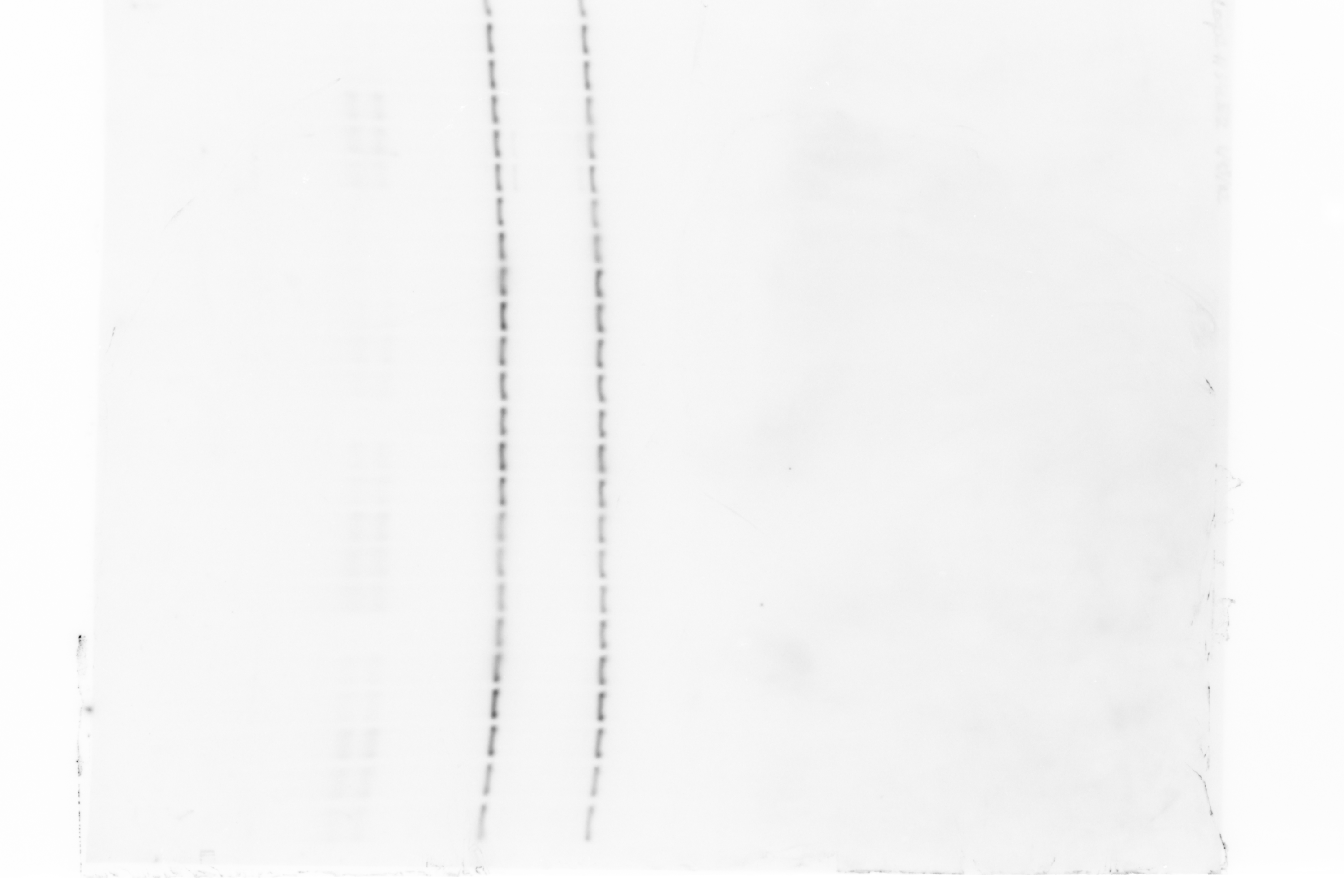

Supplement: Supplementary file 8 — Source Data Fig. 8A [file 44318_2024_34_MOESM8_ESM.zip › Figure8_PanelA/20220209 loop2 pch2 HIS4LEU2 1D 2 weeks-[Phosphor].tif]

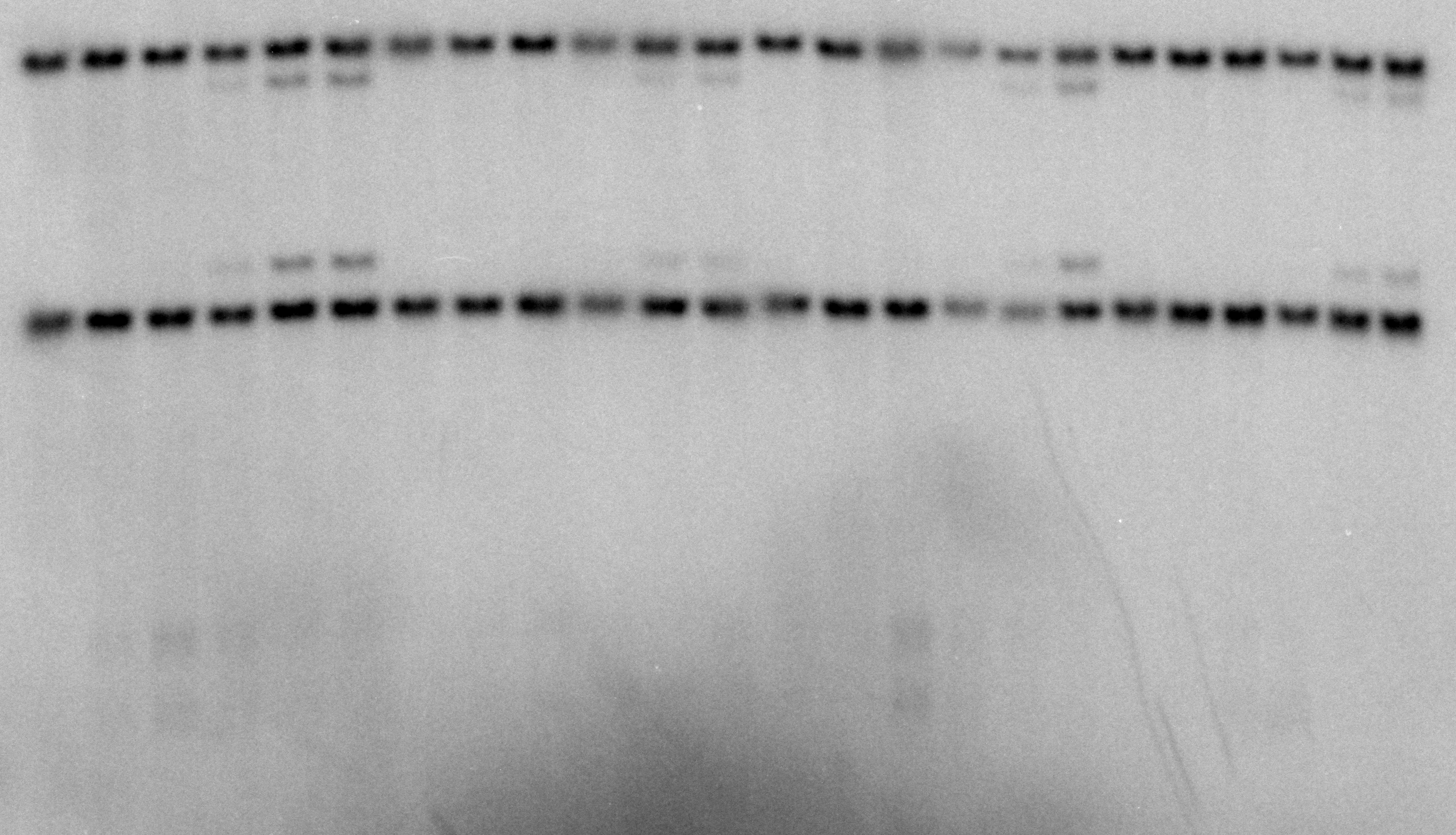

Supplement: Supplementary file 8 — Source Data Fig. 8A [file 44318_2024_34_MOESM8_ESM.zip › Figure8_PanelA/FigPanel_20231003-152325-1d-loop2-92923-[Phosphor].bmp]

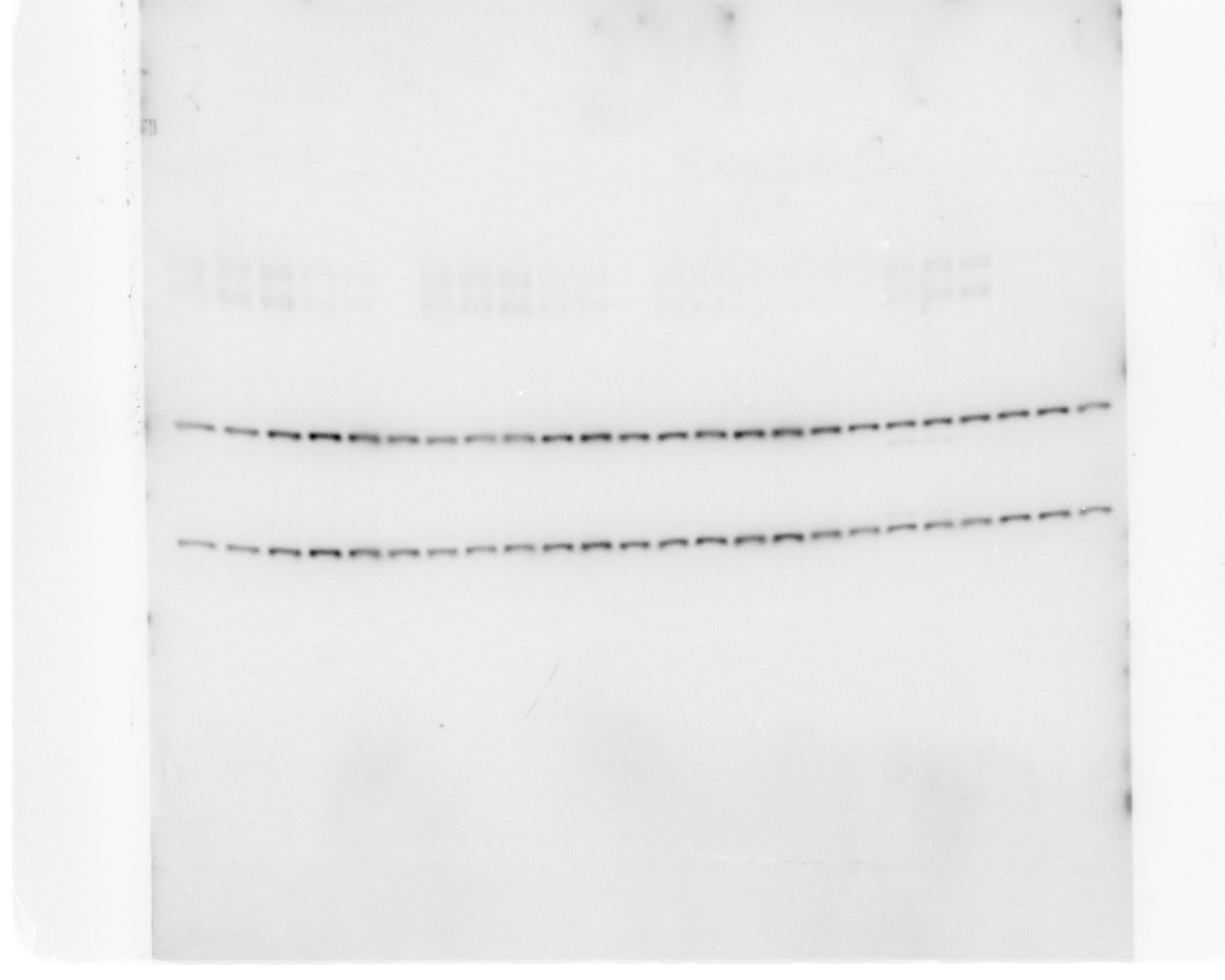

Supplement: Supplementary file 8 — Source Data Fig. 8A [file 44318_2024_34_MOESM8_ESM.zip › Figure8_PanelA/20220310 1D 4 weeks-[Phosphor].tif]

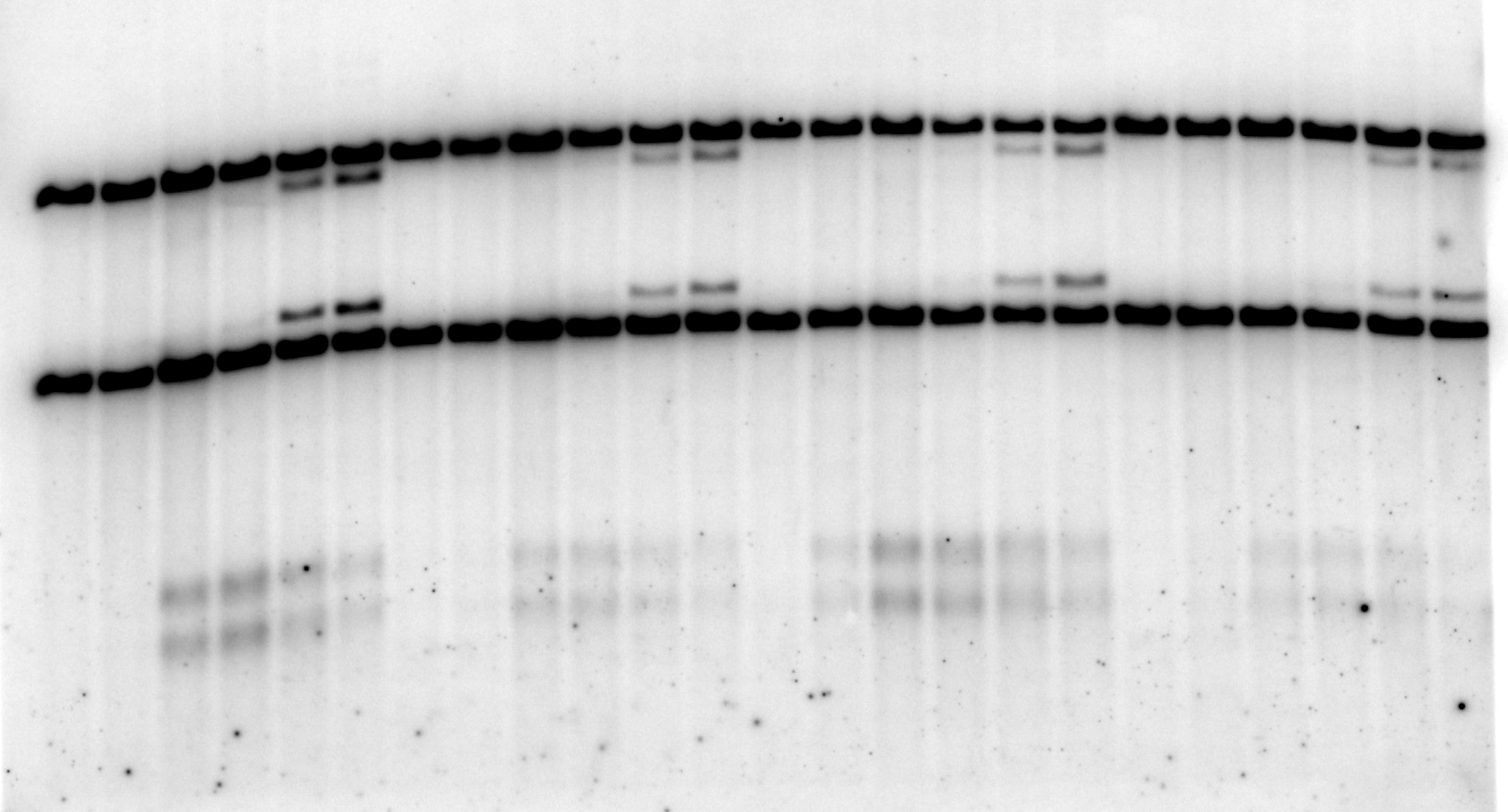

Supplement: Supplementary file 8 — Source Data Fig. 8A [file 44318_2024_34_MOESM8_ESM.zip › Figure8_PanelA/Fig_20220523 loop2 pch2 HIS4 1d 2weeks rescan-[Phosphor]-1.tif]

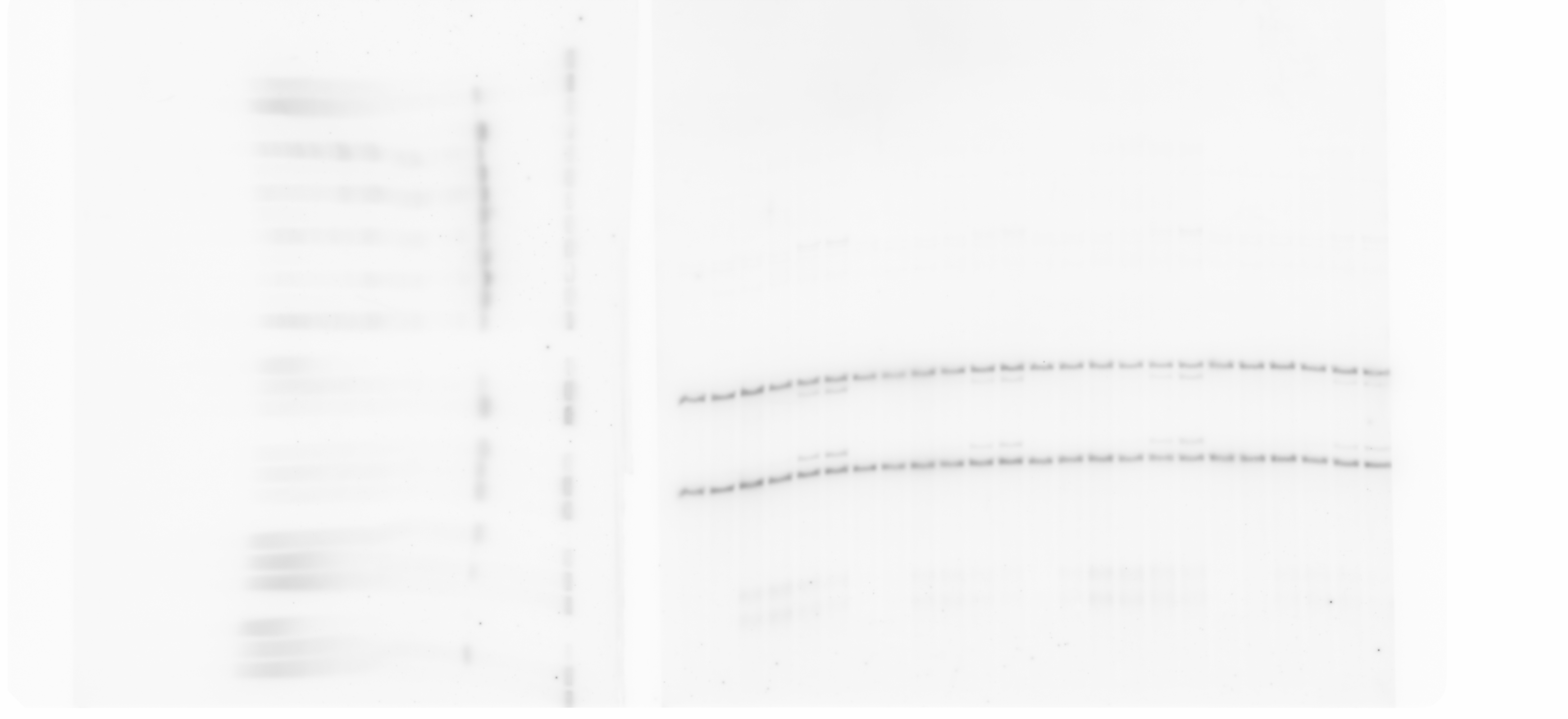

Supplement: Supplementary file 8 — Source Data Fig. 8A [file 44318_2024_34_MOESM8_ESM.zip › Figure8_PanelA/20220523 loop2 pch2 HIS4 1d 2weeks rescan-[Phosphor].tiff]

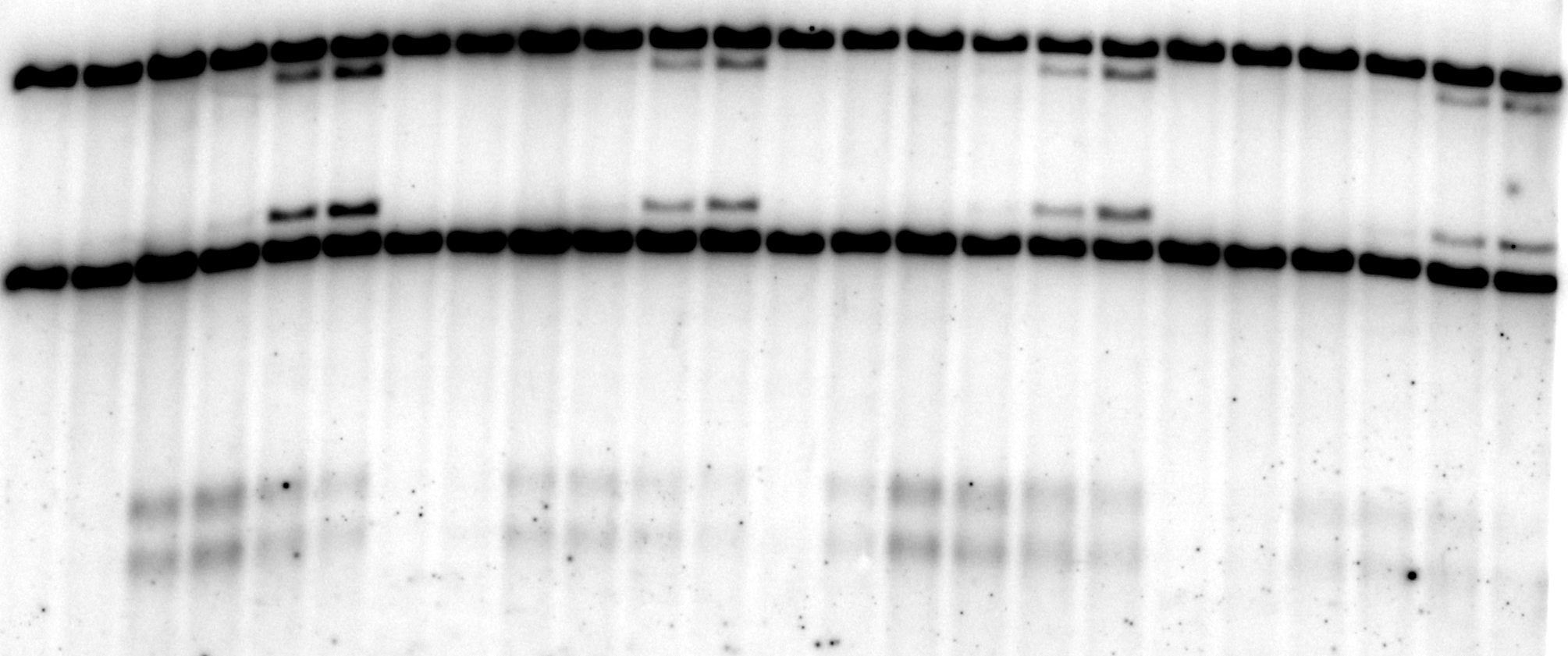

Supplement: Supplementary file 8 — Source Data Fig. 8A [file 44318_2024_34_MOESM8_ESM.zip › Figure8_PanelA/ForFigure_20220523 loop2 pch2 HIS4 1d 2weeks rescan-[Phosphor].tif]

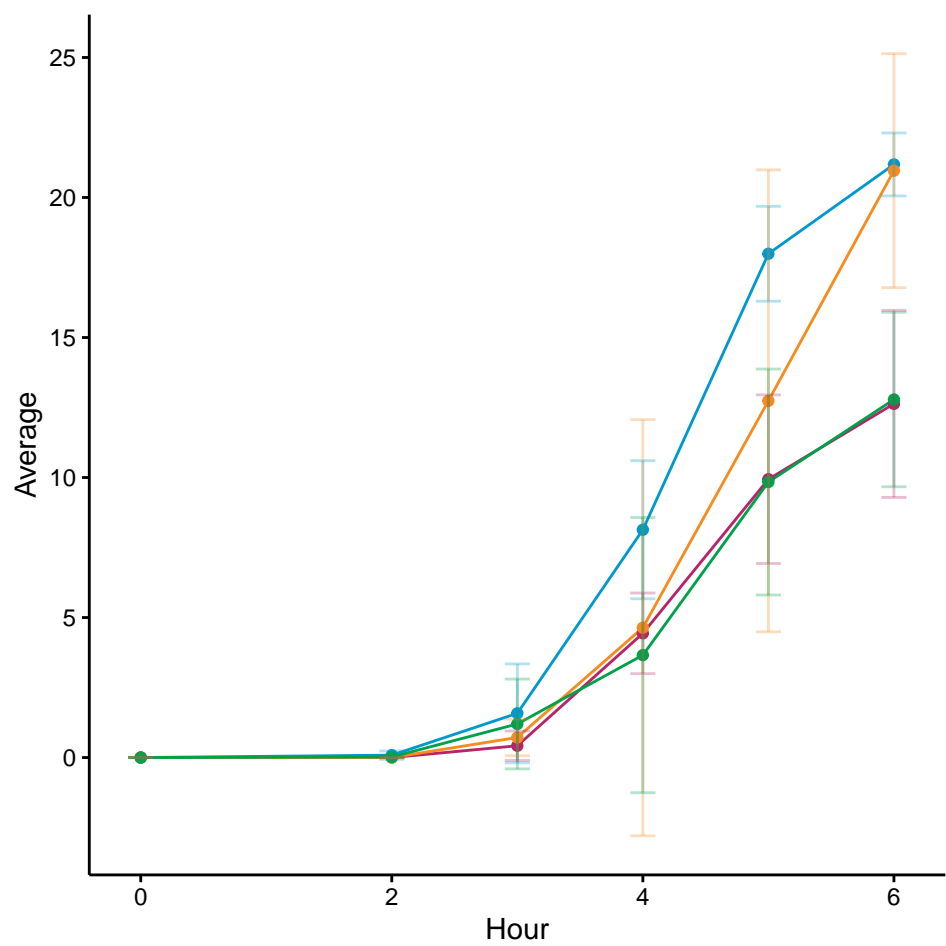

Supplement: Supplementary file 8 — Source Data Fig. 8A [file 44318_2024_34_MOESM8_ESM.zip › Figure8_PanelA/HIS4LEU2-1D-CO_nolegend.pdf]

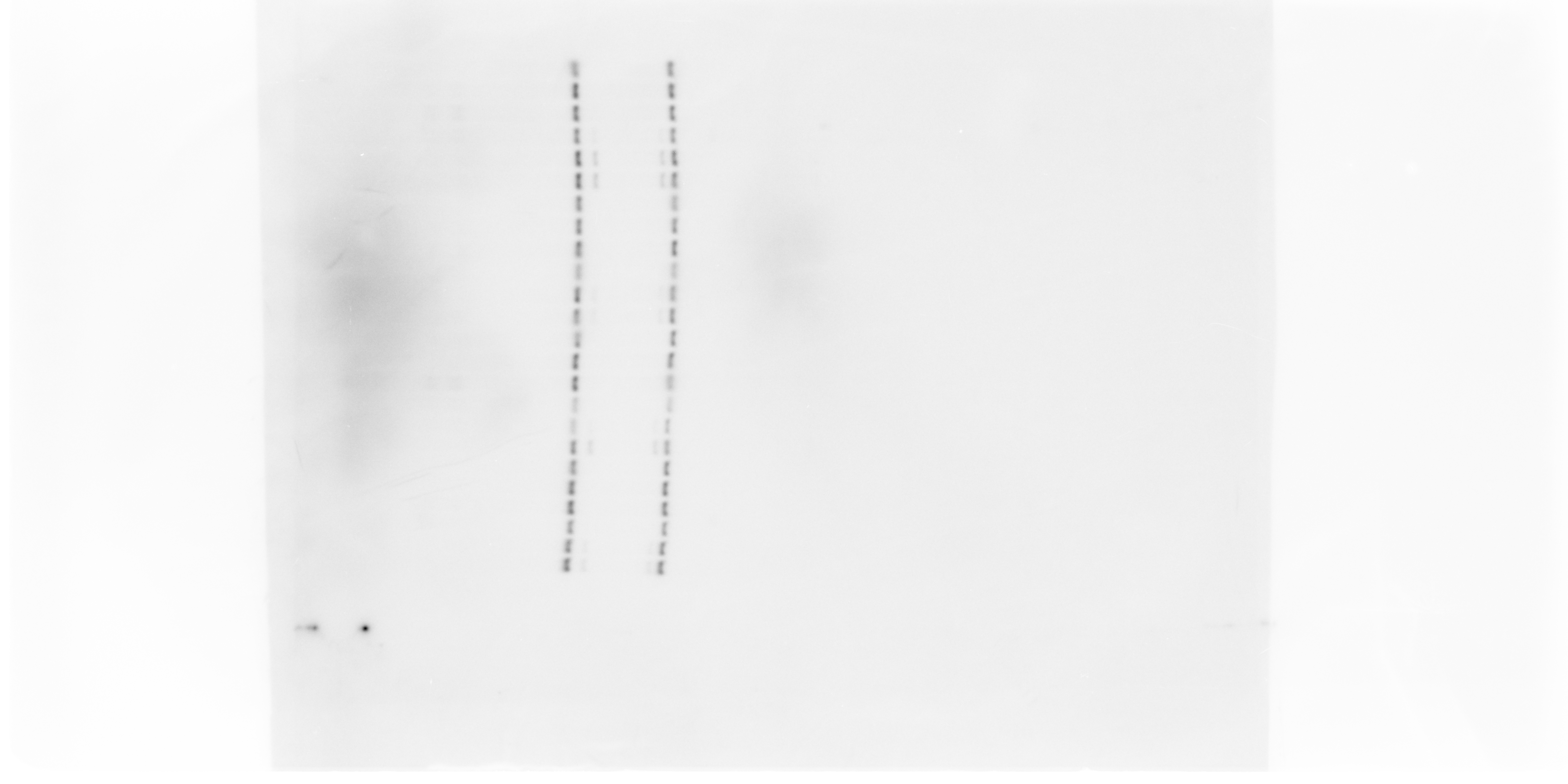

Supplement: Supplementary file 8 — Source Data Fig. 8A [file 44318_2024_34_MOESM8_ESM.zip › Figure8_PanelA/20231003-152325-1d-loop2-92923-[Phosphor].tif]

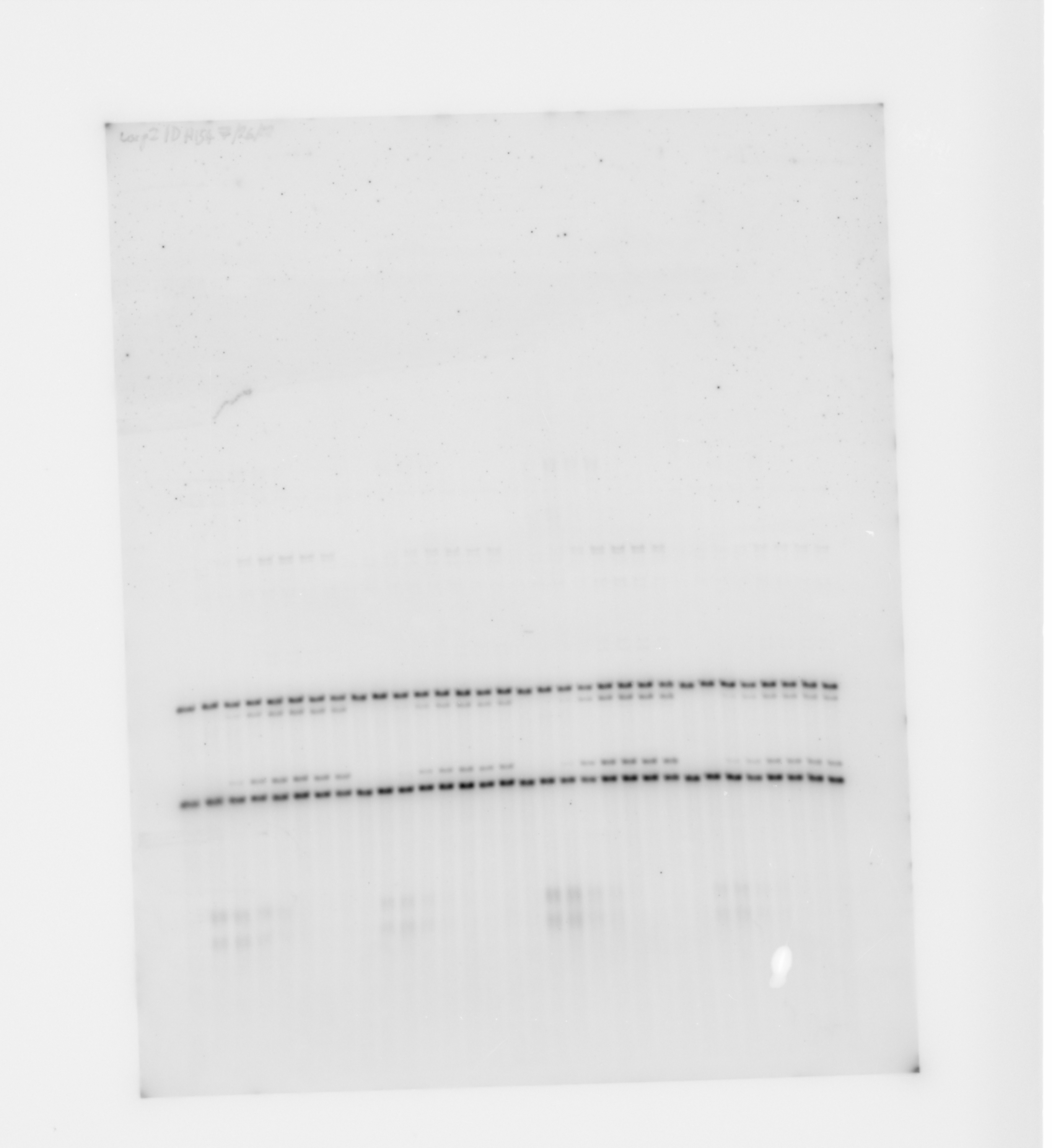

Supplement: Supplementary file 8 — Source Data Fig. 8A [file 44318_2024_34_MOESM8_ESM.zip › Figure8_PanelA/1D_cal_Analysis20221013/20221013 loop2 pch2 1D HIS4LEU2 40days-[Phosphor]-1.tif]

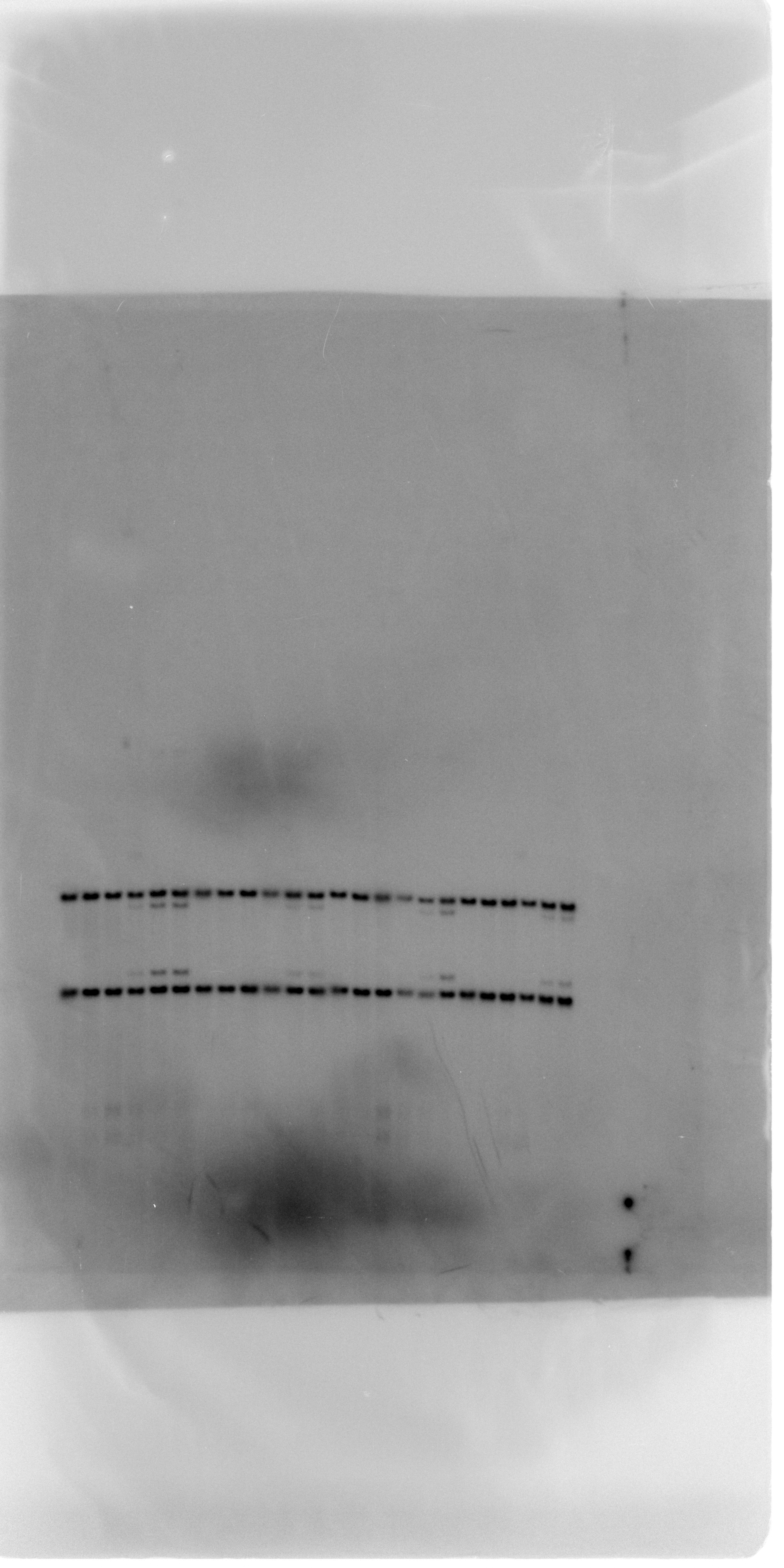

Supplement: Supplementary file 8 — Source Data Fig. 8A [file 44318_2024_34_MOESM8_ESM.zip › Figure8_PanelA/20231003-152325-1d-loop2-92923_analyzed/20231003-152325-1d-loop2-92923-[Phosphor]_measured.tif]

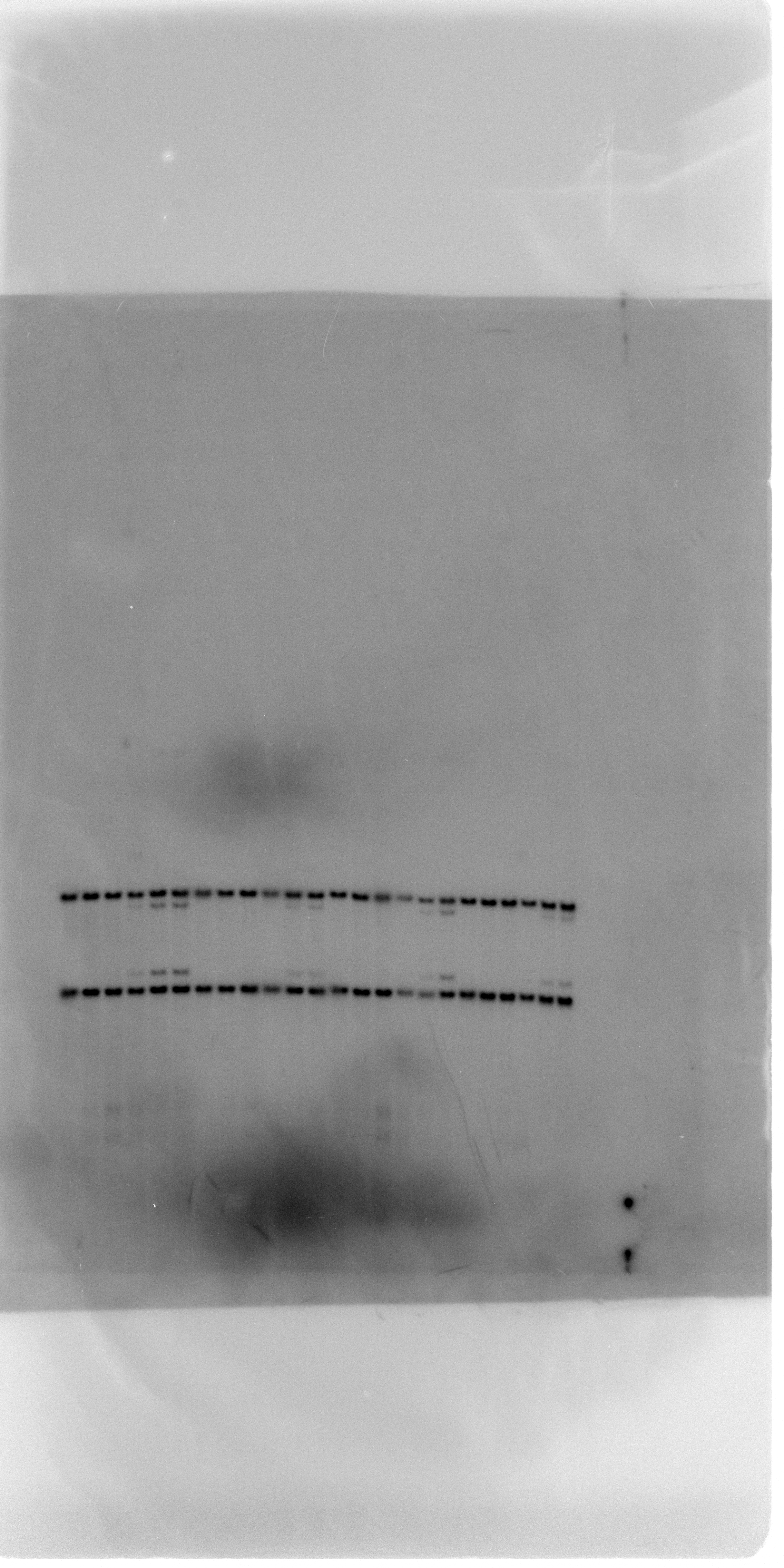

Supplement: Supplementary file 8 — Source Data Fig. 8A [file 44318_2024_34_MOESM8_ESM.zip › Figure8_PanelA/20231003-152325-1d-loop2-92923_analyzed/20231003-152325-1d-loop2-92923-[Phosphor].tif]

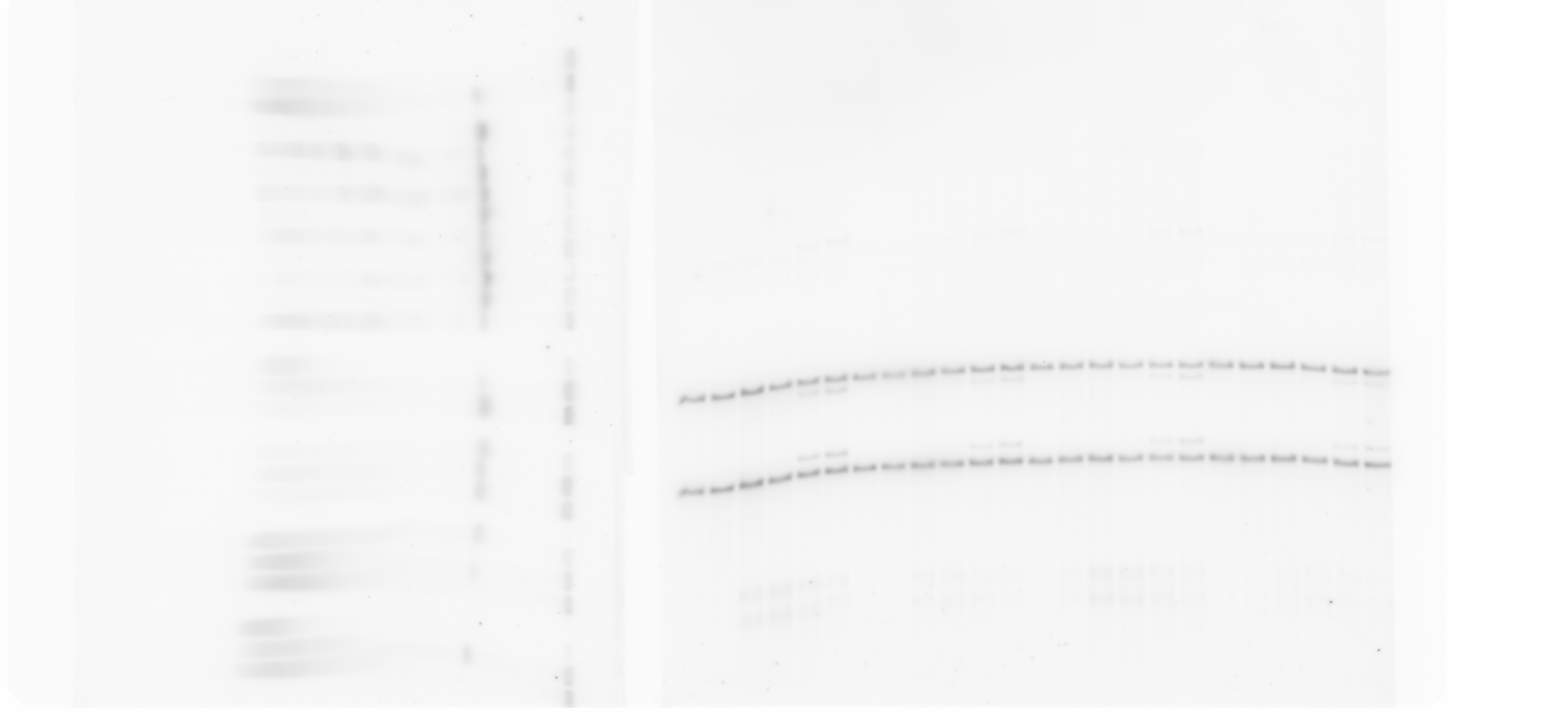

Supplement: Supplementary file 8 — Source Data Fig. 8A [file 44318_2024_34_MOESM8_ESM.zip › Figure8_PanelA/20220523_Analyzed/20220523 loop2 pch2 HIS4 1d 2weeks rescan-[Phosphor]-measured.tif]

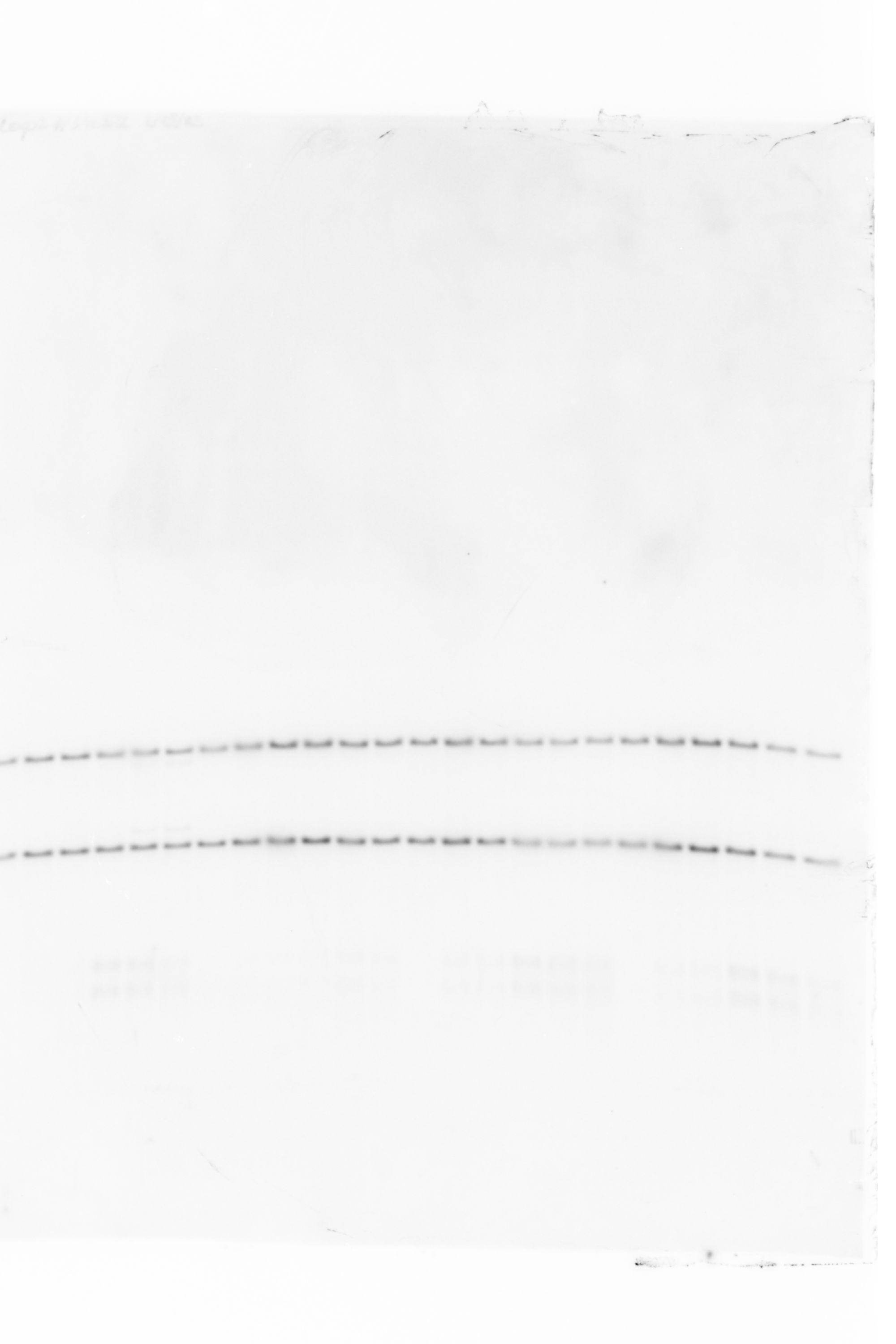

Supplement: Supplementary file 8 — Source Data Fig. 8A [file 44318_2024_34_MOESM8_ESM.zip › Figure8_PanelA/20220209/measured_20220209 loop2 pch2 HIS4LEU2 1D 2 weeks-[Phosphor].tif]

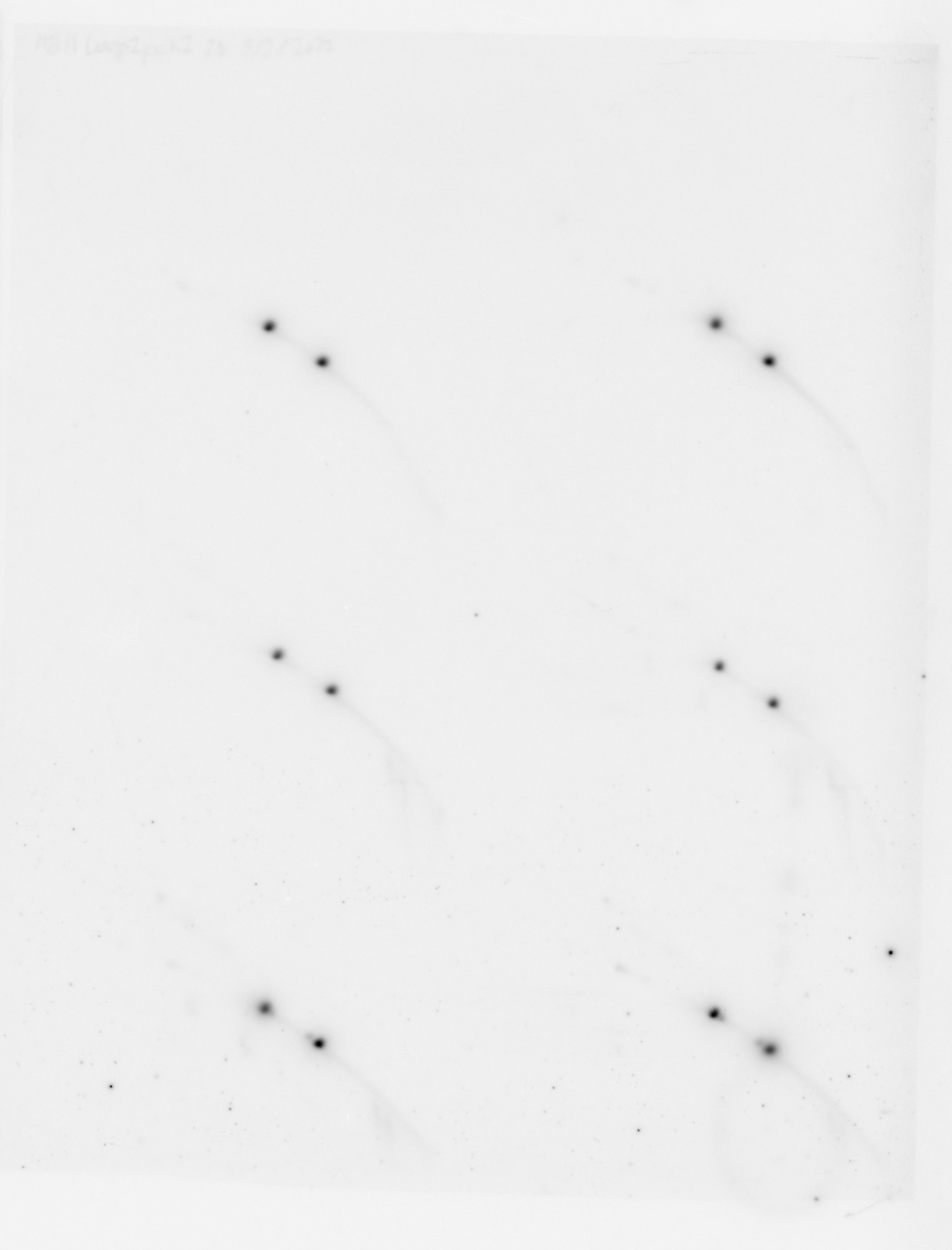

Supplement: Supplementary file 9 — Source Data Fig. 8B [file 44318_2024_34_MOESM9_ESM.zip › Figure8_PanelB/loop2_pch2_2D_20220523 loop2 pch2 HIS4 2d 2weeks-[Phosphor]-1-measured.tif]

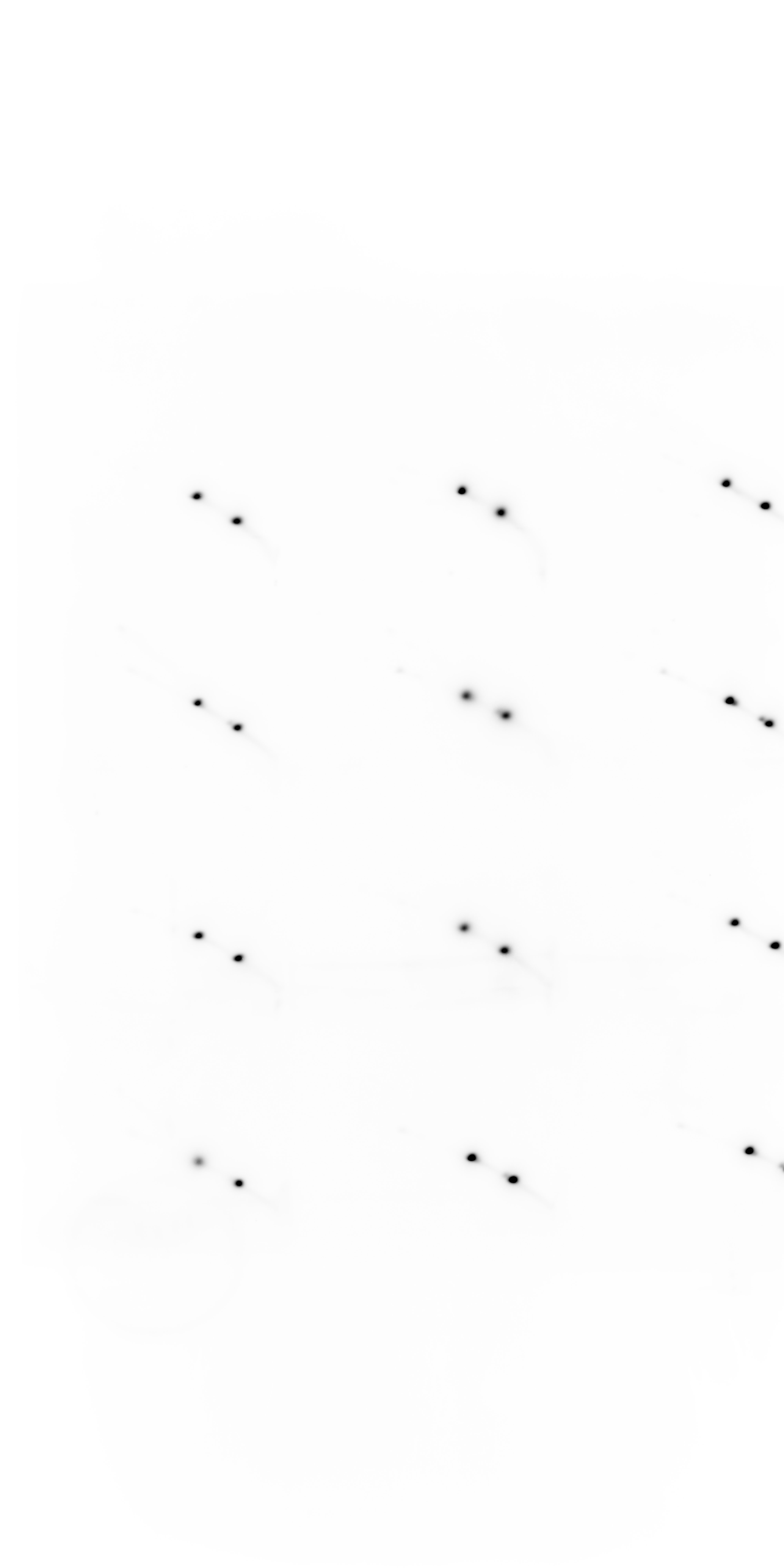

Supplement: Supplementary file 9 — Source Data Fig. 8B [file 44318_2024_34_MOESM9_ESM.zip › Figure8_PanelB/20231023-173905-11276-11688--[Phosphor]-2-measured.tif]

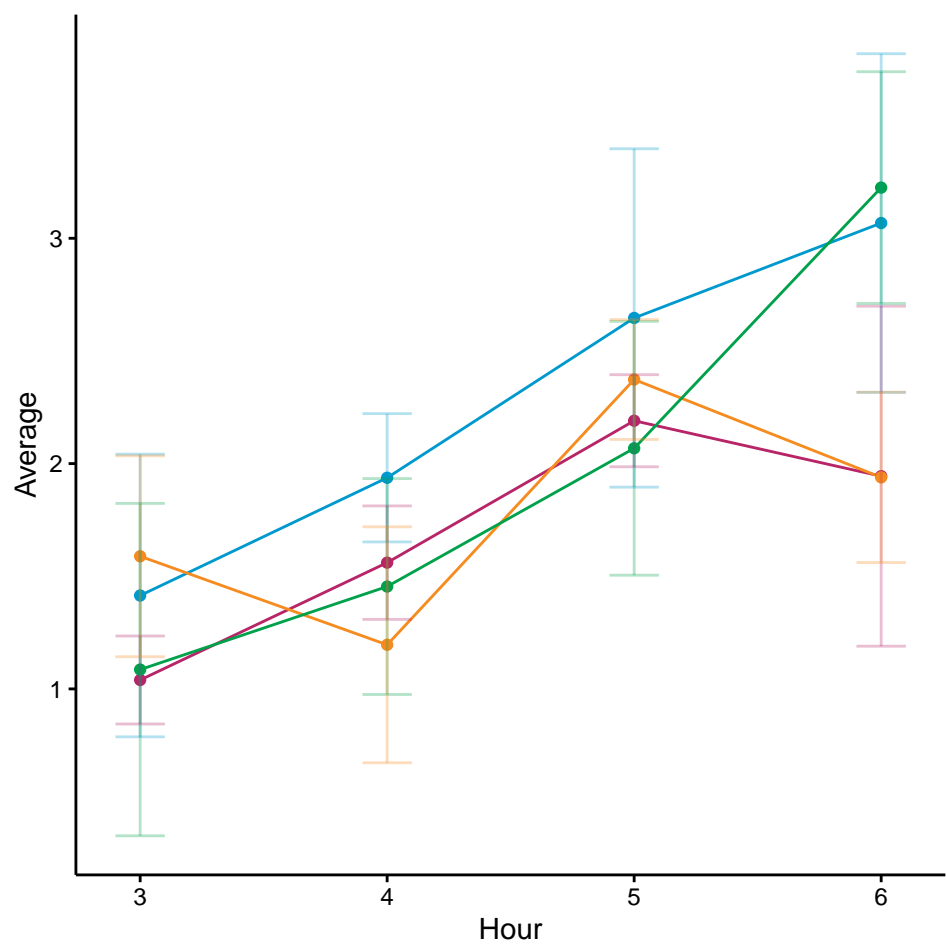

Supplement: Supplementary file 9 — Source Data Fig. 8B [file 44318_2024_34_MOESM9_ESM.zip › Figure8_PanelB/HIS4LEU2-2D_nolegend.pdf]

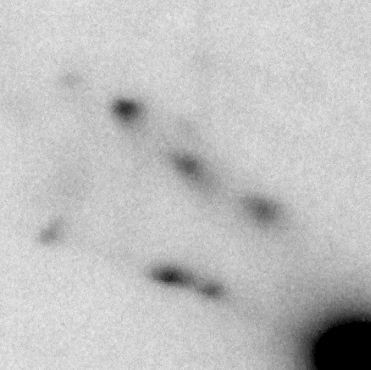

Supplement: Supplementary file 9 — Source Data Fig. 8B [file 44318_2024_34_MOESM9_ESM.zip › Figure8_PanelB/loop2Panel20220209 loop2 HIS4LEU2 2D 7 days-[Phosphor]-1-measured-1.bmp]

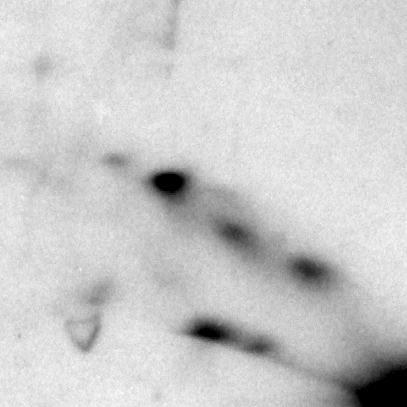

Supplement: Supplementary file 9 — Source Data Fig. 8B [file 44318_2024_34_MOESM9_ESM.zip › Figure8_PanelB/pch2_ForFig_panel_20220209 loop2 HIS4LEU2 2D 7 days-[Phosphor]-1-measured-1.bmp]

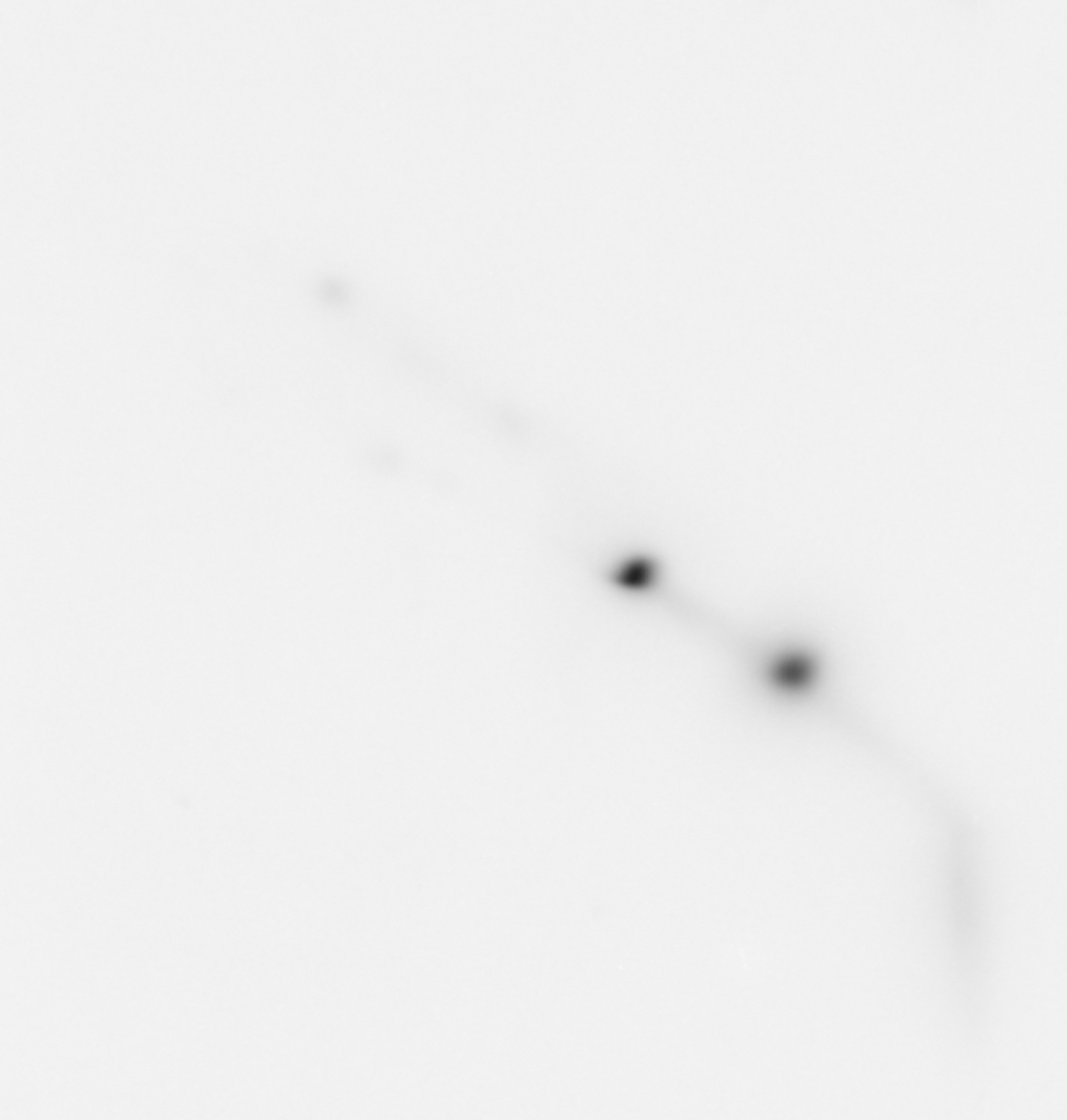

Supplement: Supplementary file 9 — Source Data Fig. 8B [file 44318_2024_34_MOESM9_ESM.zip › Figure8_PanelB/loop2-pch2_second_20231023-173905-11811-11812--[Phosphor]-1.bmp]

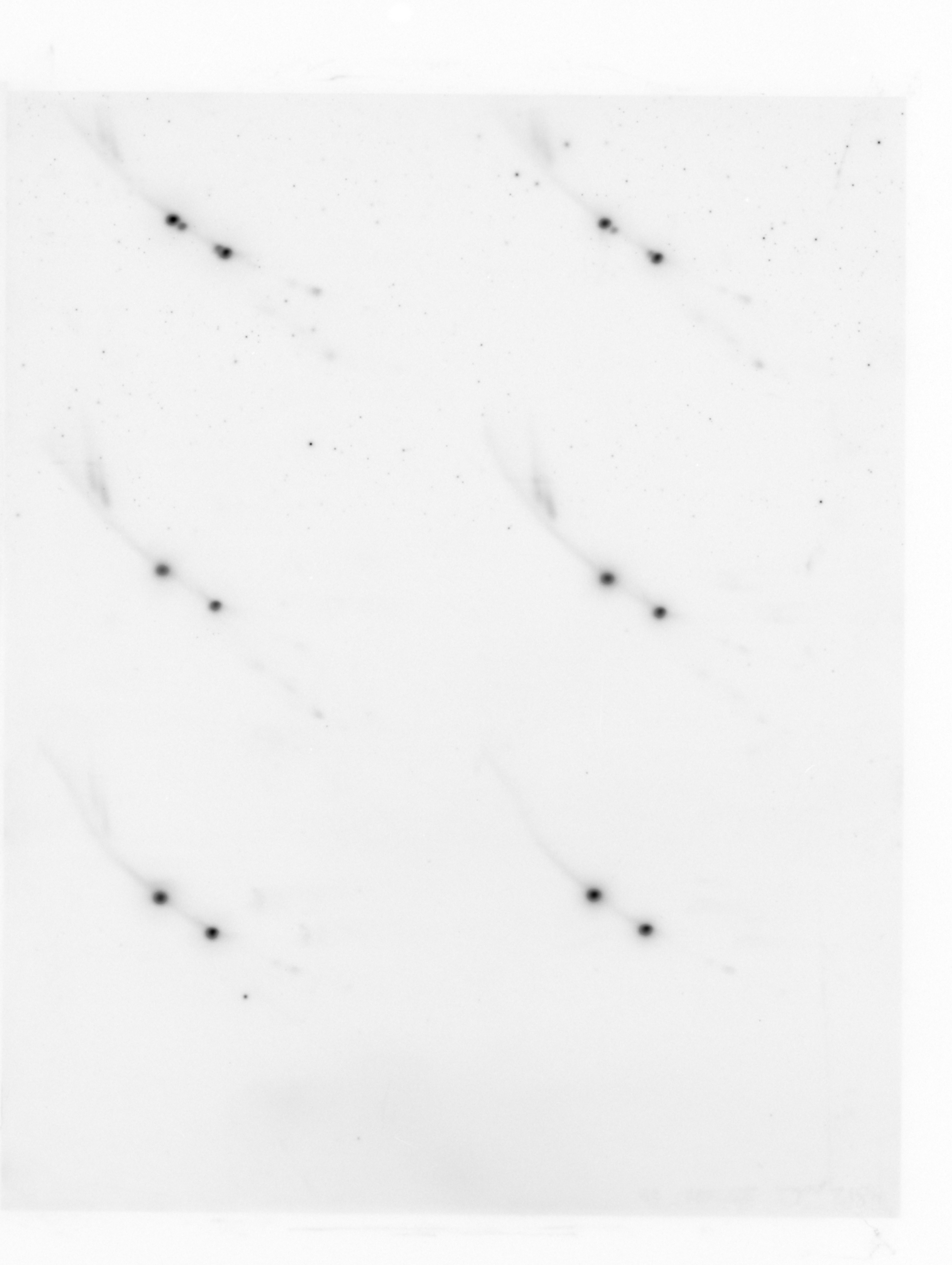

Supplement: Supplementary file 9 — Source Data Fig. 8B [file 44318_2024_34_MOESM9_ESM.zip › Figure8_PanelB/pch2_2D_20220523 loop2 pch2 HIS4 2d 2weeks-[Phosphor]-1.tif]

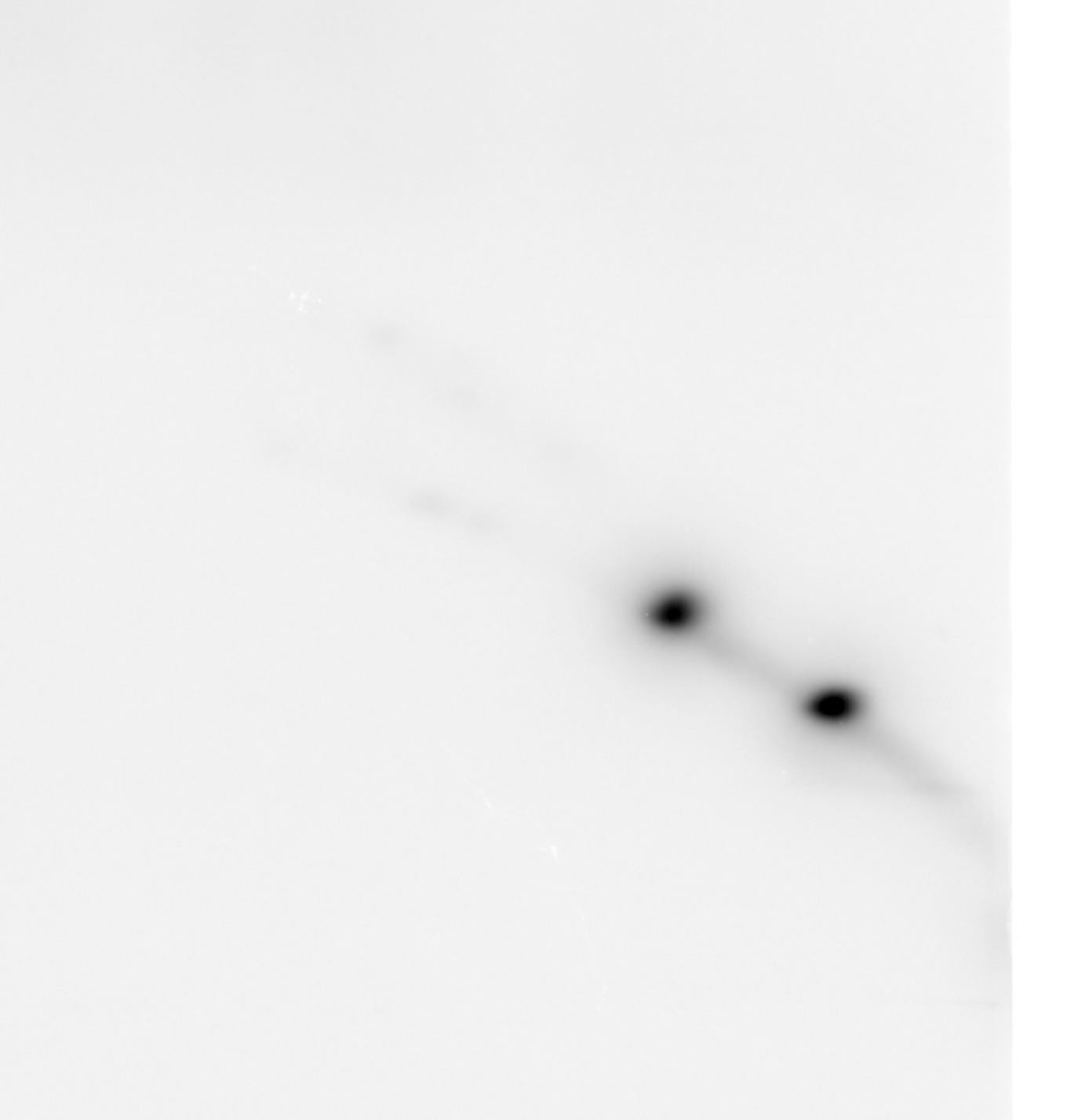

Supplement: Supplementary file 9 — Source Data Fig. 8B [file 44318_2024_34_MOESM9_ESM.zip › Figure8_PanelB/loop2pch2_tryagain_20231023-173905-11811-11812--[Phosphor]-1.bmp]

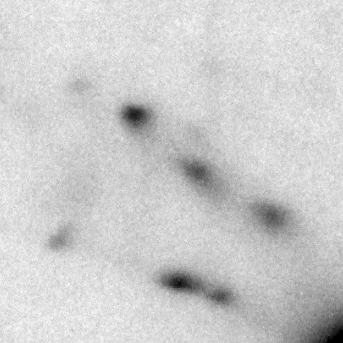

Supplement: Supplementary file 9 — Source Data Fig. 8B [file 44318_2024_34_MOESM9_ESM.zip › Figure8_PanelB/loop2Panel-2-20220209 loop2 HIS4LEU2 2D 7 days-[Phosphor]-1-measured-1.bmp]

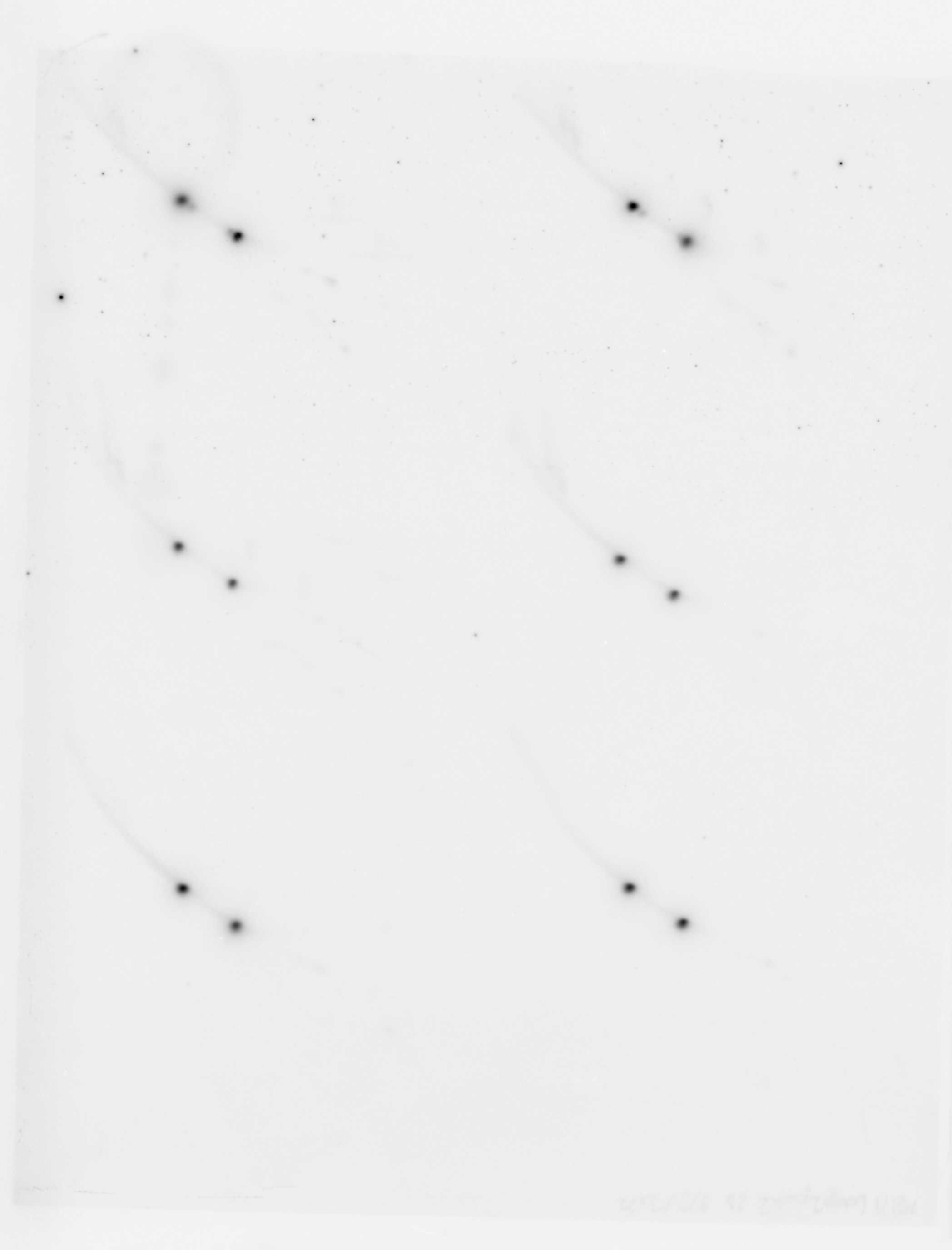

Supplement: Supplementary file 9 — Source Data Fig. 8B [file 44318_2024_34_MOESM9_ESM.zip › Figure8_PanelB/loop2_pch2_2D_20220523 loop2 pch2 HIS4 2d 2weeks-[Phosphor]-1.tif]

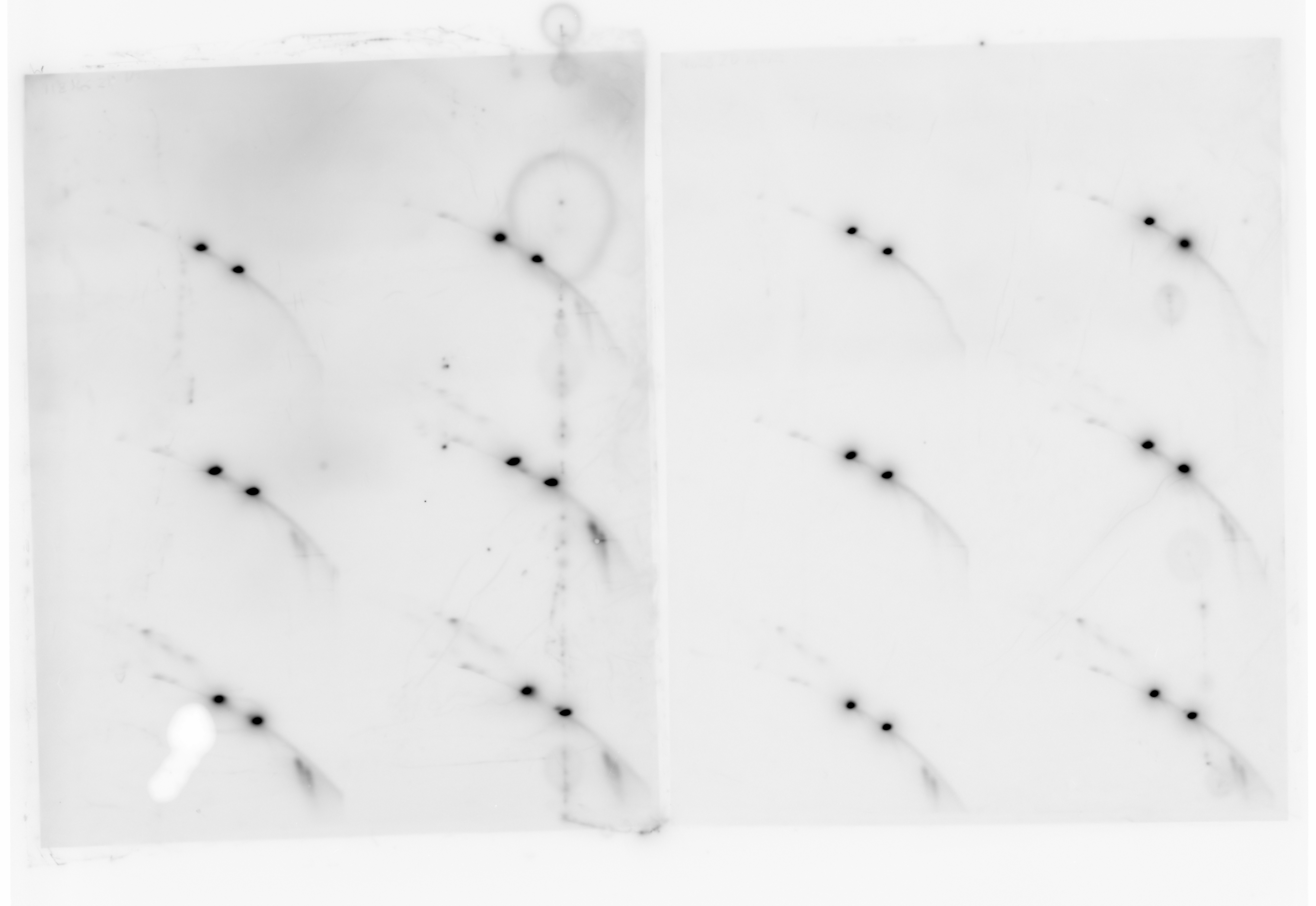

Supplement: Supplementary file 9 — Source Data Fig. 8B [file 44318_2024_34_MOESM9_ESM.zip › Figure8_PanelB/20220209 loop2 HIS4LEU2 2D 7 days-[Phosphor]-1-measured.tif]

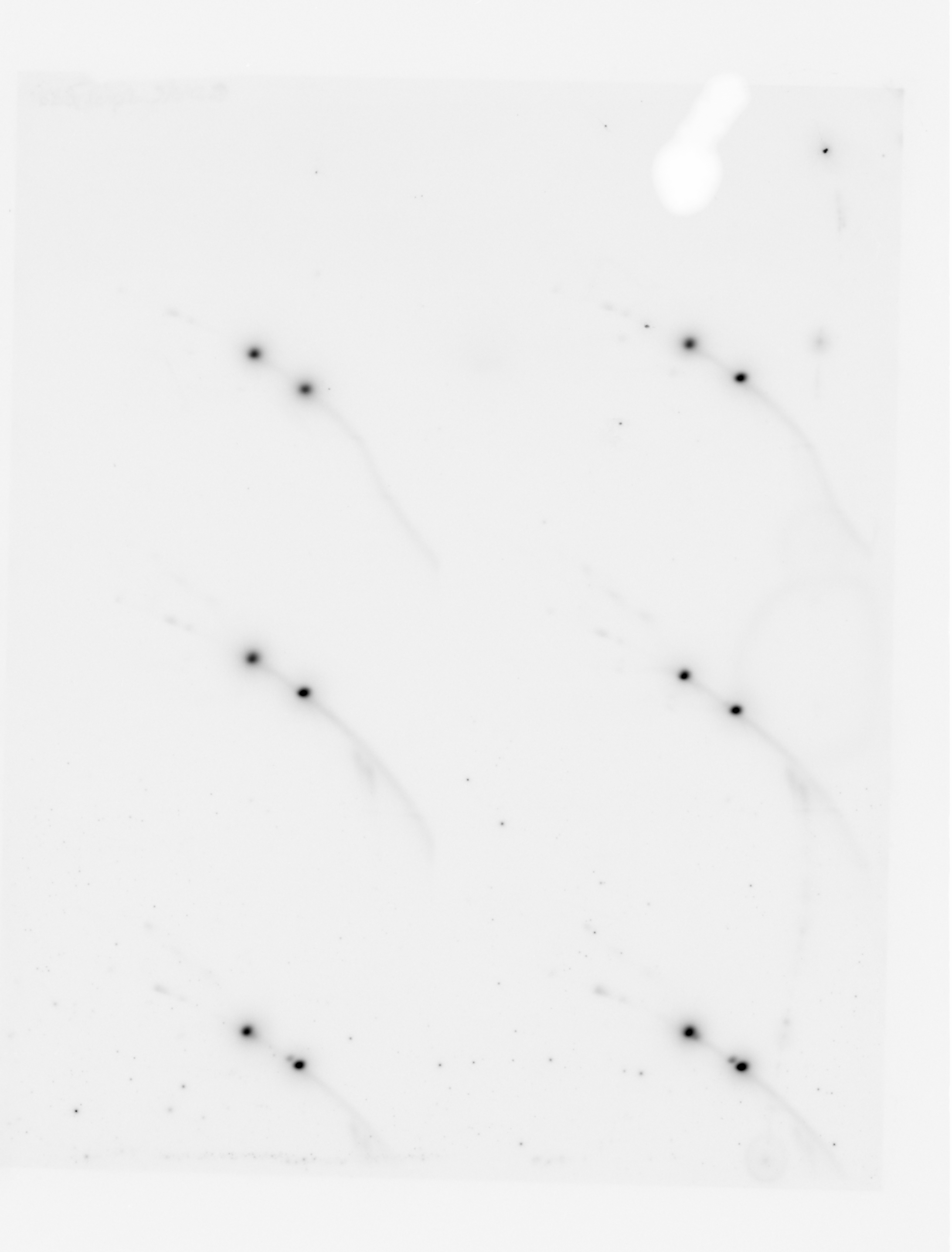

Supplement: Supplementary file 9 — Source Data Fig. 8B [file 44318_2024_34_MOESM9_ESM.zip › Figure8_PanelB/loop2_2D_20220523 loop2 WT HIS4 2d 2weeks-[Phosphor]-1.tif]

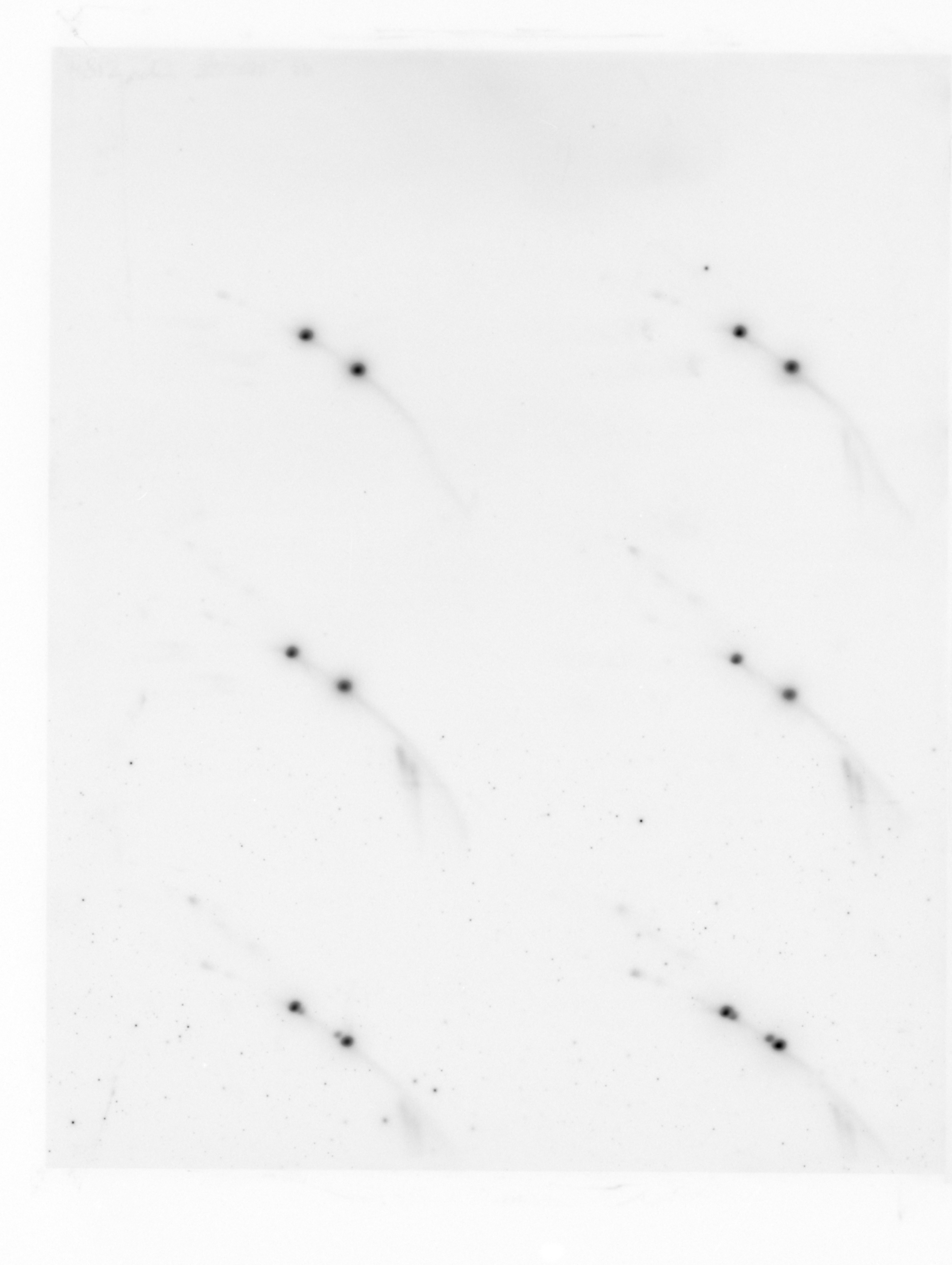

Supplement: Supplementary file 9 — Source Data Fig. 8B [file 44318_2024_34_MOESM9_ESM.zip › Figure8_PanelB/pch2_2D_20220523 loop2 pch2 HIS4 2d 2weeks-[Phosphor]-1-Measured.tif]

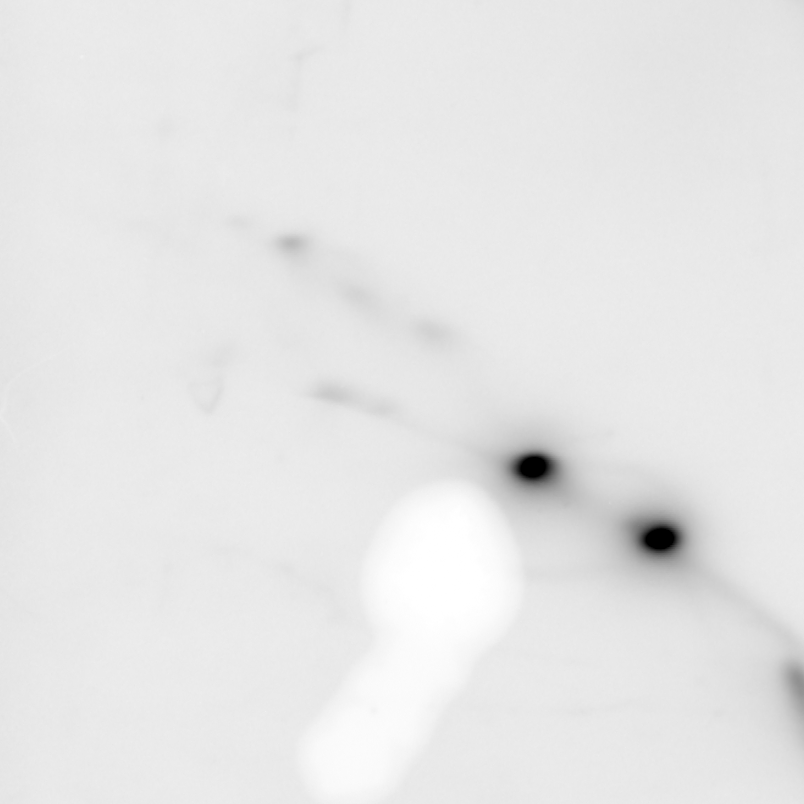

Supplement: Supplementary file 9 — Source Data Fig. 8B [file 44318_2024_34_MOESM9_ESM.zip › Figure8_PanelB/pch2_panel_20220209 loop2 HIS4LEU2 2D 7 days-[Phosphor]-1-measured-1.bmp]

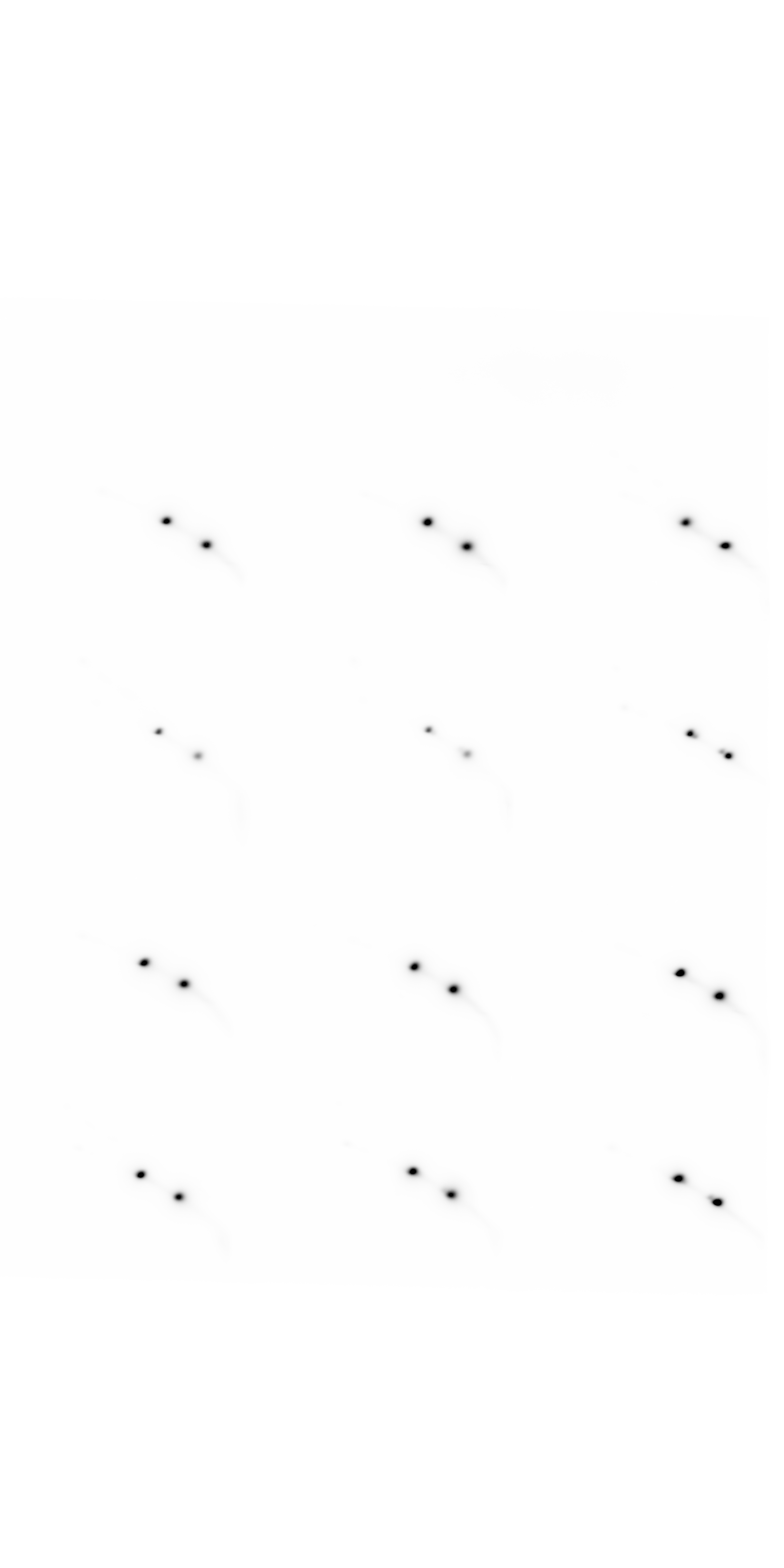

Supplement: Supplementary file 9 — Source Data Fig. 8B [file 44318_2024_34_MOESM9_ESM.zip › Figure8_PanelB/20231023-173905-11811-11812--[Phosphor]-1-Measured.tif]

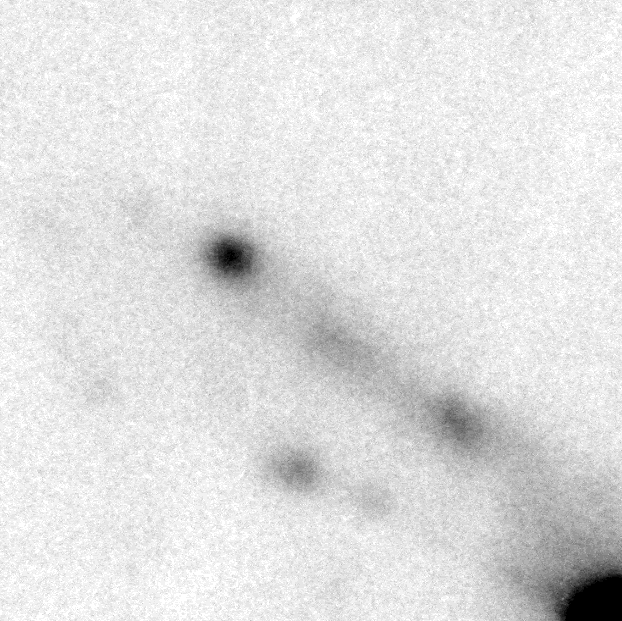

Supplement: Supplementary file 9 — Source Data Fig. 8B [file 44318_2024_34_MOESM9_ESM.zip › Figure8_PanelB/loop2-pch2_second_edits_20231023-173905-11811-11812--[Phosphor]-1.bmp]

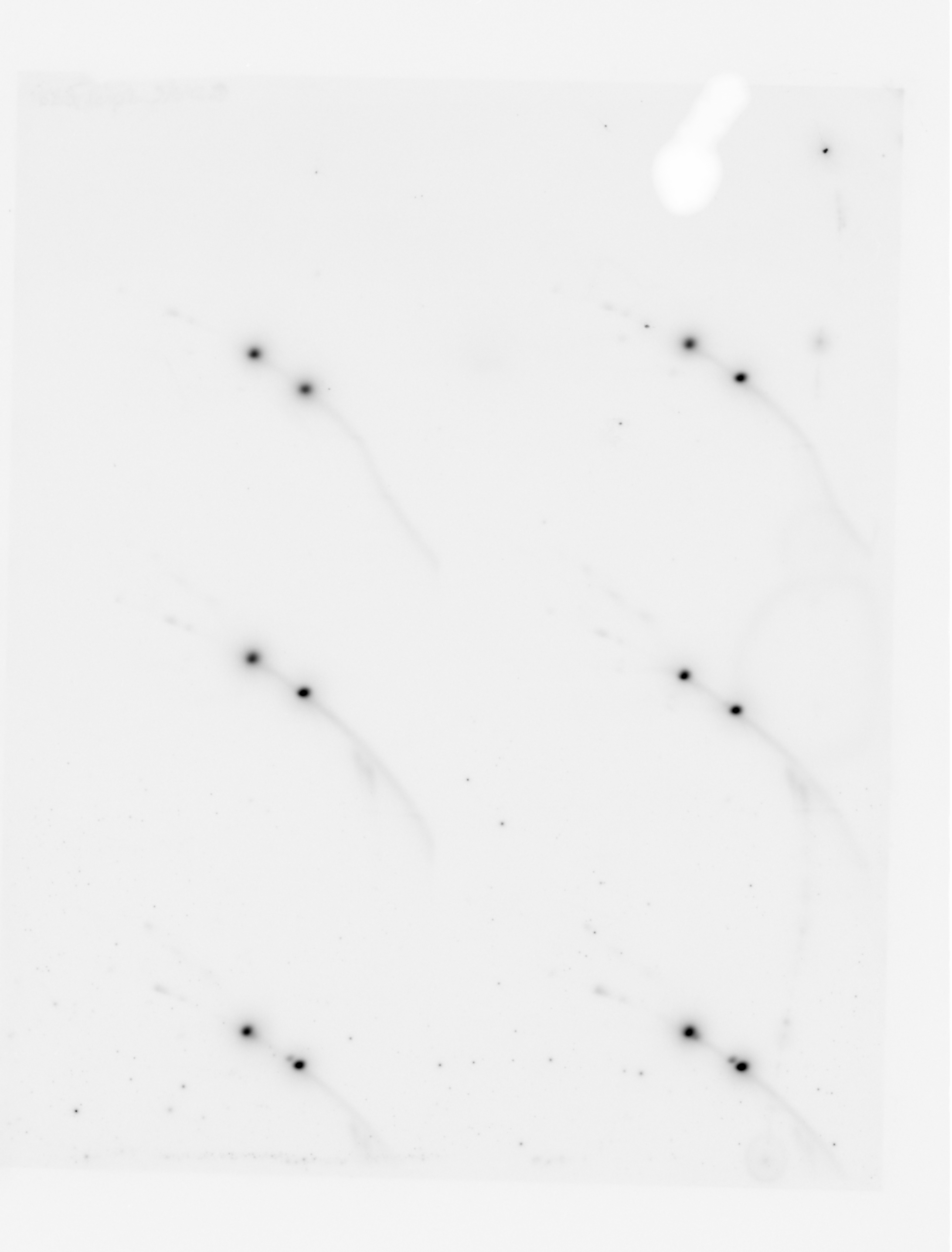

Supplement: Supplementary file 9 — Source Data Fig. 8B [file 44318_2024_34_MOESM9_ESM.zip › Figure8_PanelB/loop2_2D_20220523 loop2 WT HIS4 2d 2weeks-[Phosphor]-1-Measured.tif]

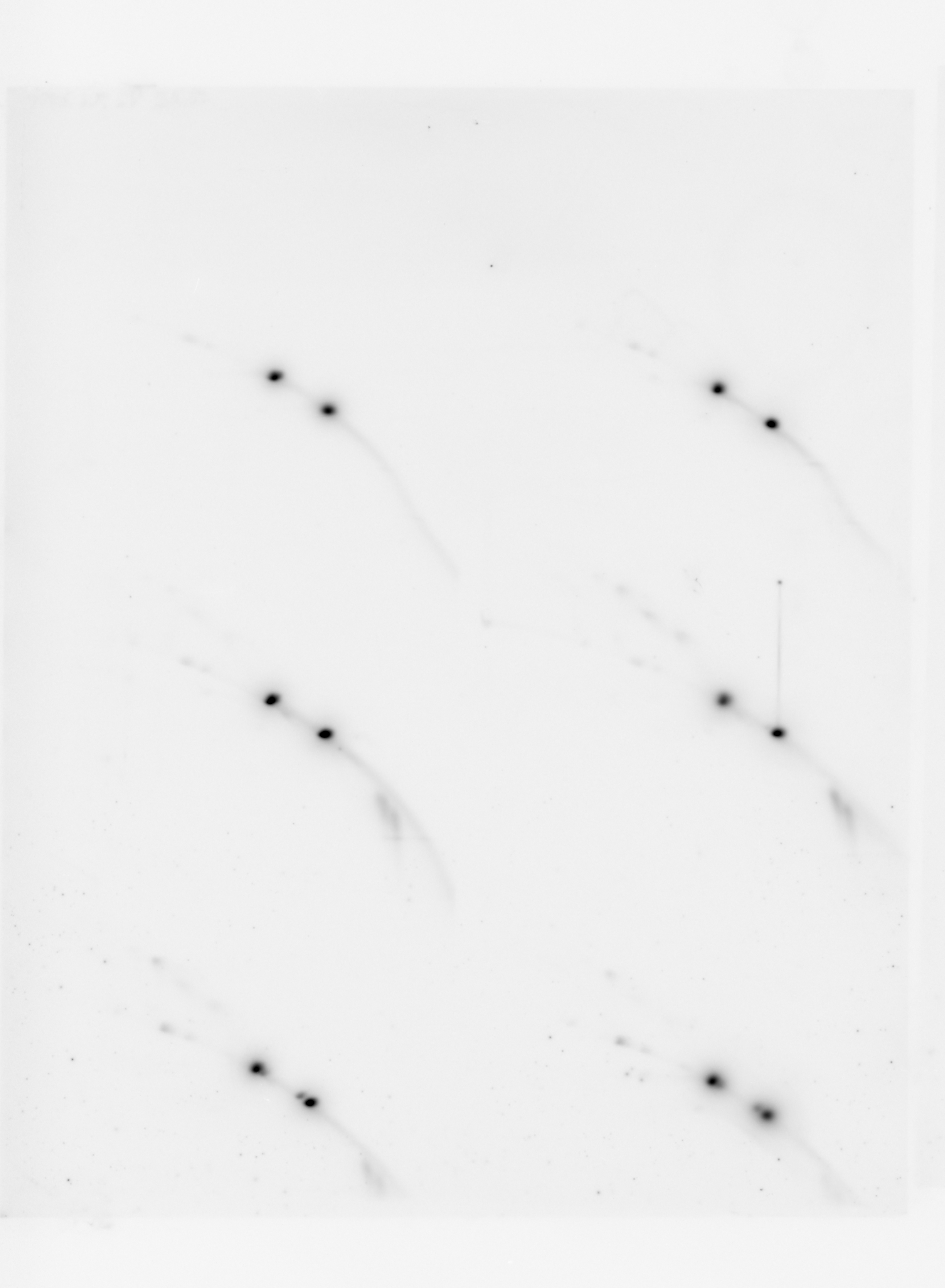

Supplement: Supplementary file 9 — Source Data Fig. 8B [file 44318_2024_34_MOESM9_ESM.zip › Figure8_PanelB/Wt_2D_20220523 loop2 WT HIS4 2d 2weeks-[Phosphor]-1-measured.tif]

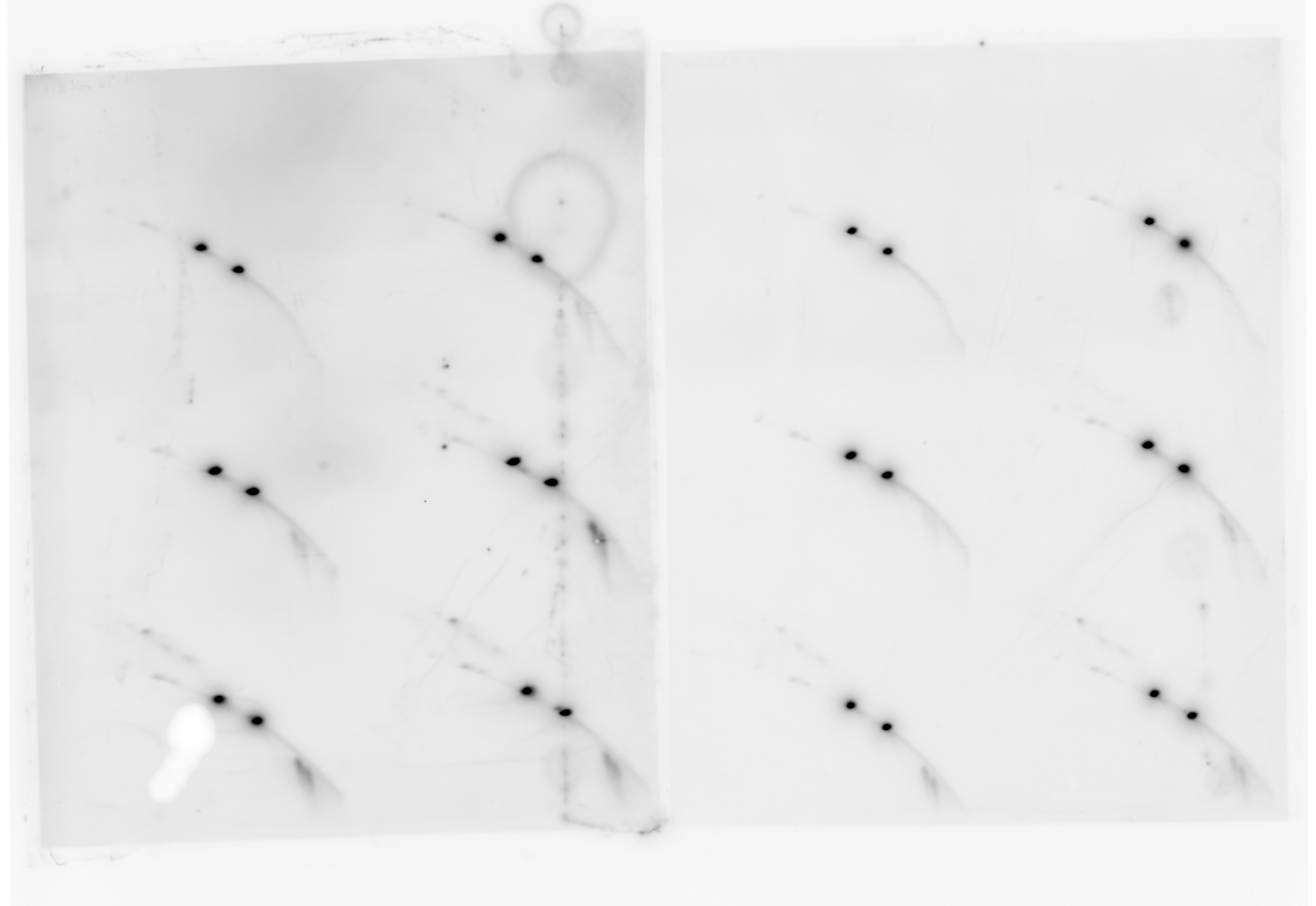

Supplement: Supplementary file 9 — Source Data Fig. 8B [file 44318_2024_34_MOESM9_ESM.zip › Figure8_PanelB/20220209 loop2 HIS4LEU2 2D 7 days-[Phosphor].gel]

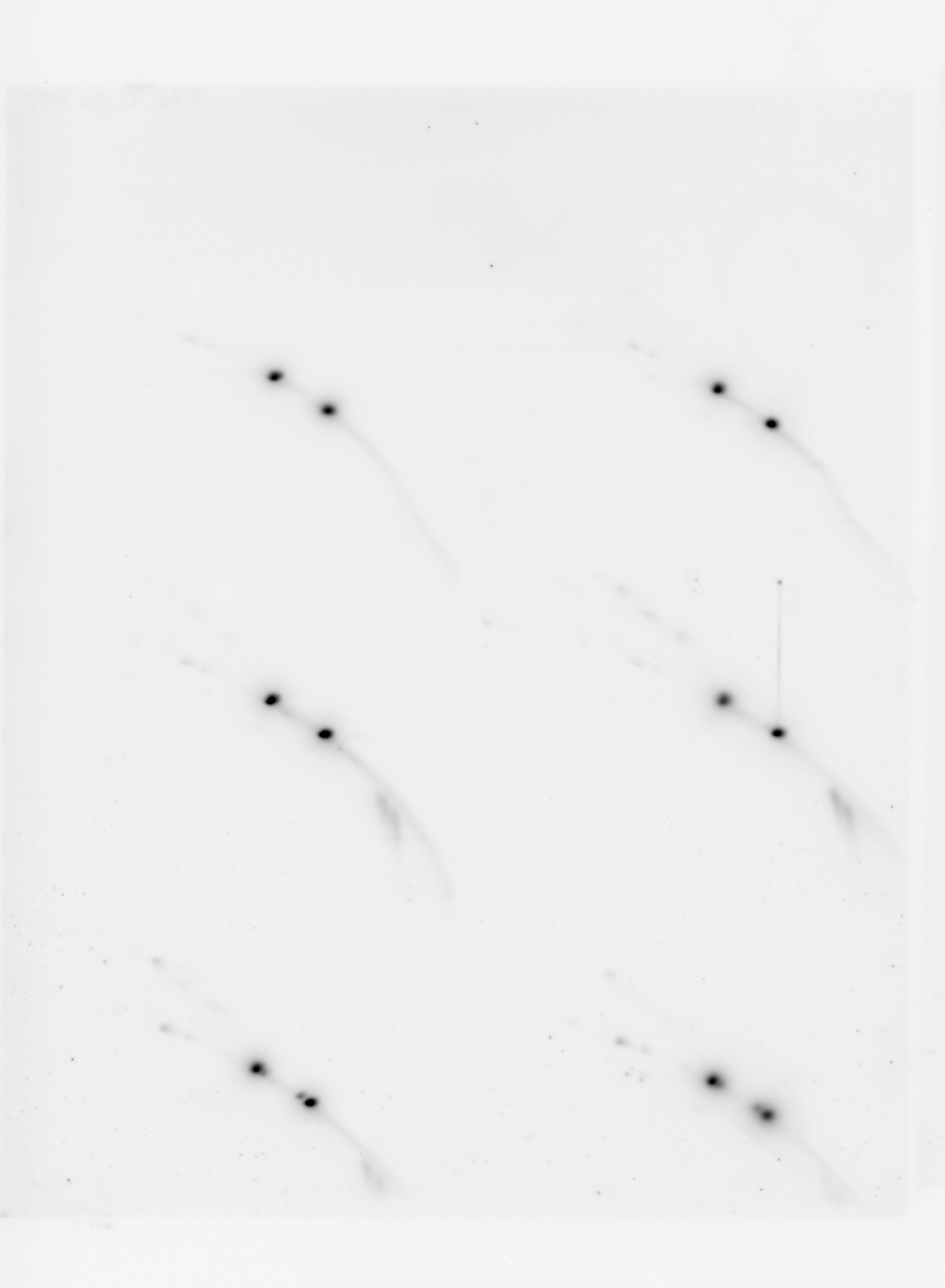

Supplement: Supplementary file 9 — Source Data Fig. 8B [file 44318_2024_34_MOESM9_ESM.zip › Figure8_PanelB/Wt_2D_20220523 loop2 WT HIS4 2d 2weeks-[Phosphor]-1.tif]

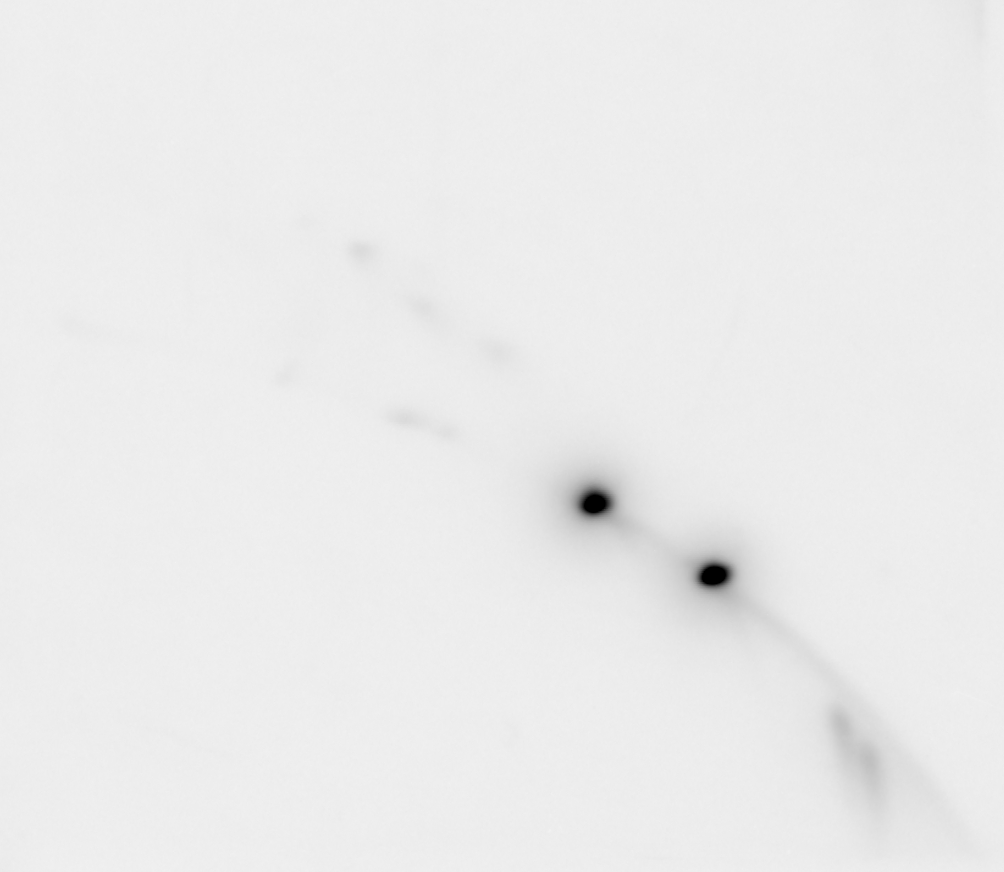

Supplement: Supplementary file 9 — Source Data Fig. 8B [file 44318_2024_34_MOESM9_ESM.zip › Figure8_PanelB/loop2Panel20220209 loop2 HIS4LEU2 2D 7 days-[Phosphor]-1-measured-1.tif]

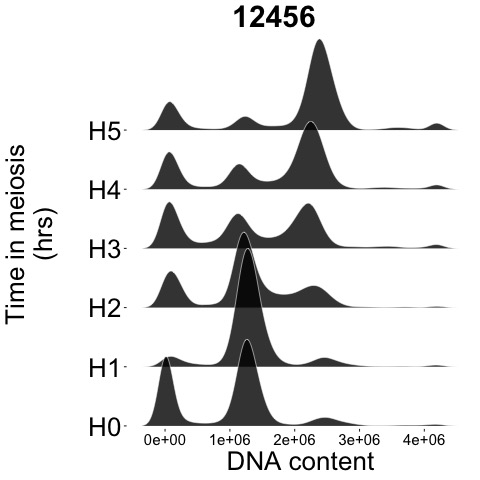

Supplement: Supplementary file 11 — Figure EV3 BC Source Data [file 44318_2024_34_MOESM11_ESM.zip › Data_Figure_EV3_BC/FACS/MTC8042023/12456.jpg]

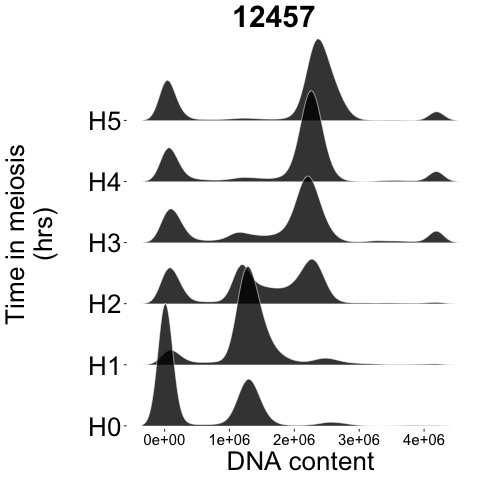

Supplement: Supplementary file 11 — Figure EV3 BC Source Data [file 44318_2024_34_MOESM11_ESM.zip › Data_Figure_EV3_BC/FACS/MTC8042023/12457.jpg]

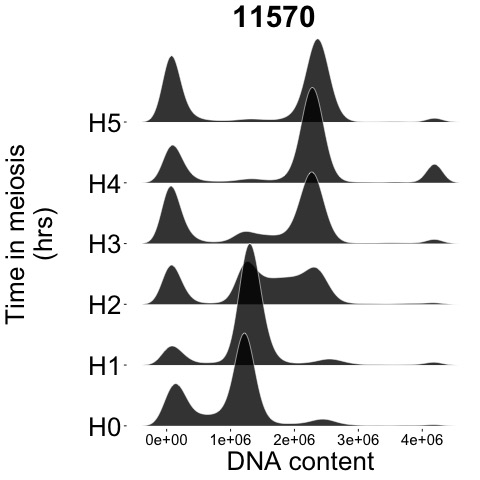

Supplement: Supplementary file 11 — Figure EV3 BC Source Data [file 44318_2024_34_MOESM11_ESM.zip › Data_Figure_EV3_BC/FACS/MTC8042023/11570.jpg]

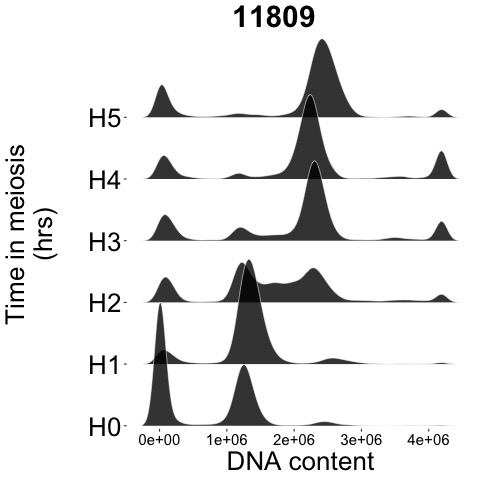

Supplement: Supplementary file 11 — Figure EV3 BC Source Data [file 44318_2024_34_MOESM11_ESM.zip › Data_Figure_EV3_BC/FACS/MTC8042023/11809.jpg]

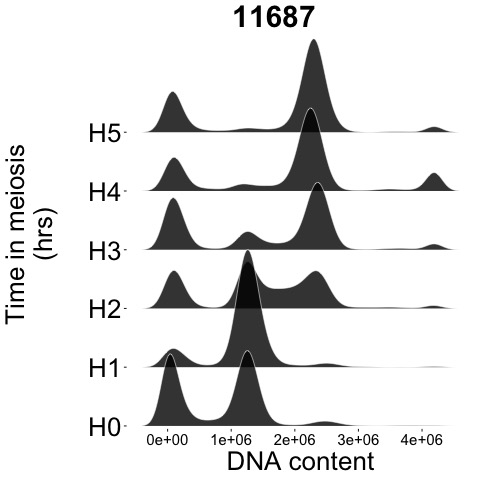

Supplement: Supplementary file 11 — Figure EV3 BC Source Data [file 44318_2024_34_MOESM11_ESM.zip › Data_Figure_EV3_BC/FACS/MTC8042023/11687.jpg]

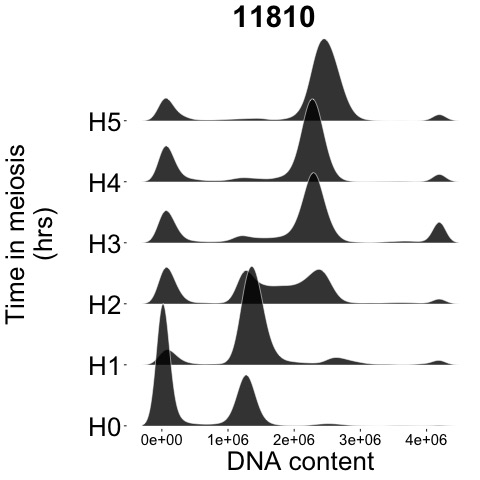

Supplement: Supplementary file 11 — Figure EV3 BC Source Data [file 44318_2024_34_MOESM11_ESM.zip › Data_Figure_EV3_BC/FACS/MTC8042023/11810.jpg]

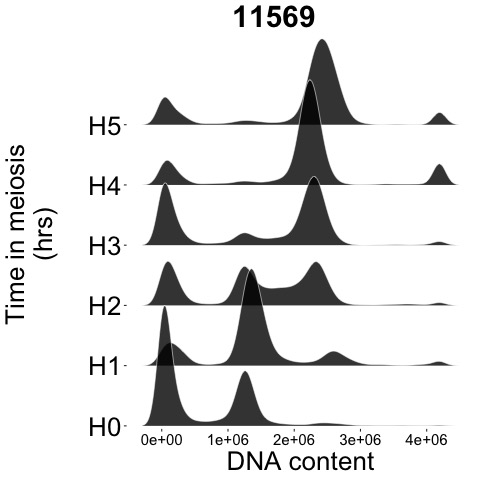

Supplement: Supplementary file 11 — Figure EV3 BC Source Data [file 44318_2024_34_MOESM11_ESM.zip › Data_Figure_EV3_BC/FACS/MTC8042023/11569.jpg]

9120

Time in meiosis  
(hrs)

H5

H4

H3

H2

H0

1e+06

2e+06

3e+06

DNA content

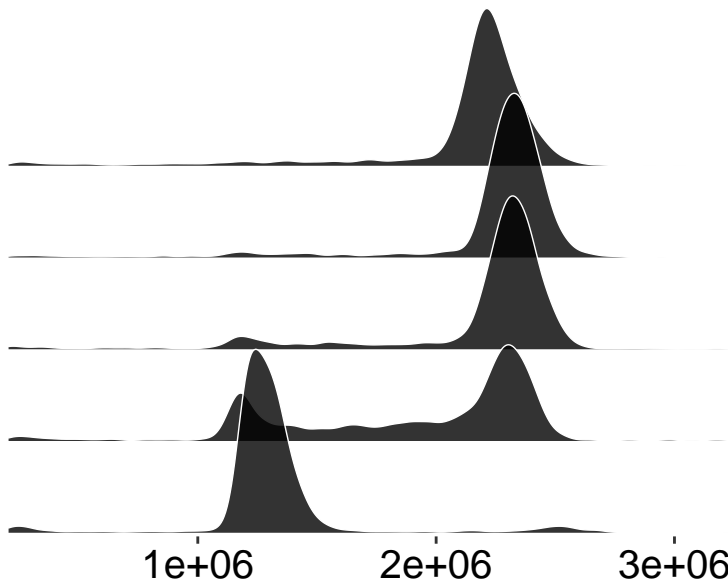

Supplement: Supplementary file 11 — Figure EV3 BC Source Data [file 44318_2024_34_MOESM11_ESM.zip › Data_Figure_EV3_BC/FACS/FACS_FigureMaterial/9120.pdf]

**7797**

Time in meiosis  
(hrs)

H5-

H4-

H3-

H2-

H0-

1e+06

2e+06

3e+06

DNA content

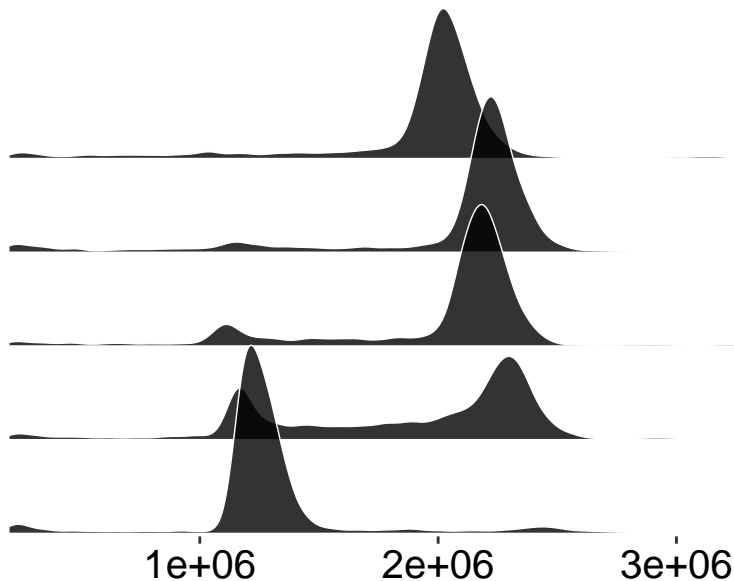

Supplement: Supplementary file 11 — Figure EV3 BC Source Data [file 44318_2024_34_MOESM11_ESM.zip › Data_Figure_EV3_BC/FACS/FACS_FigureMaterial/Wt-7797_6282022_IF.pdf]

**11644**

Time in meiosis  
(hrs)

H5

H4

H3

H2

H0

1e+06

2e+06

3e+06

DNA content

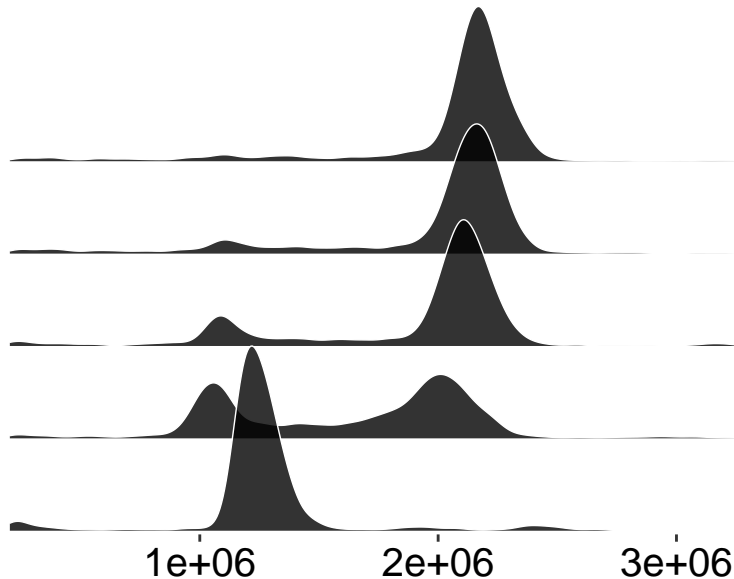

Supplement: Supplementary file 11 — Figure EV3 BC Source Data [file 44318_2024_34_MOESM11_ESM.zip › Data_Figure_EV3_BC/FACS/FACS_FigureMaterial/11644.pdf]

11757

Time in meiosis  
(hrs)

H5

H4

H3

H2

H0

1e+06

2e+06

3e+06

DNA content

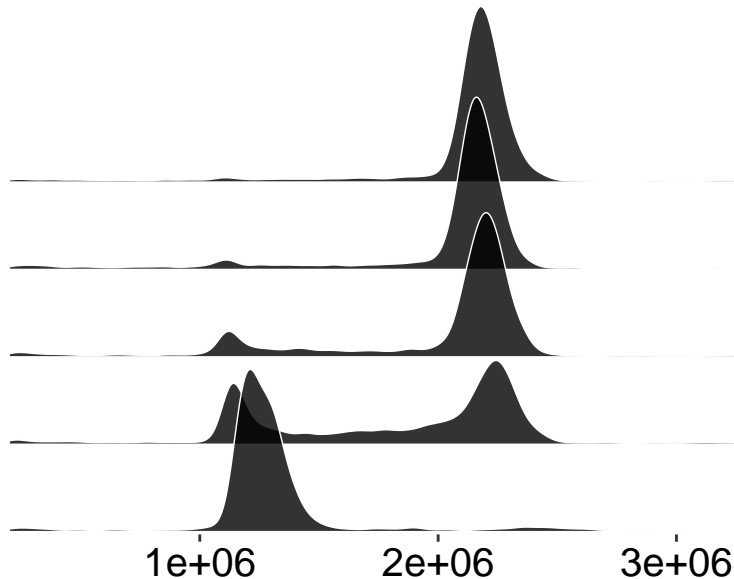

Supplement: Supplementary file 11 — Figure EV3 BC Source Data [file 44318_2024_34_MOESM11_ESM.zip › Data_Figure_EV3_BC/FACS/FACS_FigureMaterial/11757.pdf]

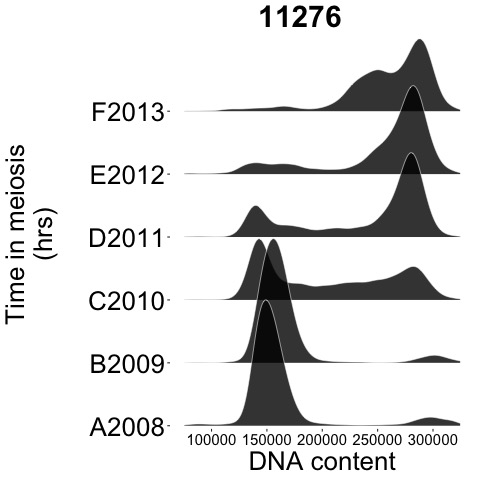

Supplement: Supplementary file 11 — Figure EV3 BC Source Data [file 44318_2024_34_MOESM11_ESM.zip › Data_Figure_EV3_BC/FACS/2023-08-29/11276.jpg]
