# Supplementary figures and images for: Chromatin binding by HORMAD proteins regulates meiotic recombination initiation (part 2 of 2)
Source: EMBO J. 2024 Feb 8;43(5):8. doi: 10.1038/s44318-024-00034-3 (PMC10907721; doi:10.1038/s44318-024-00034-3)

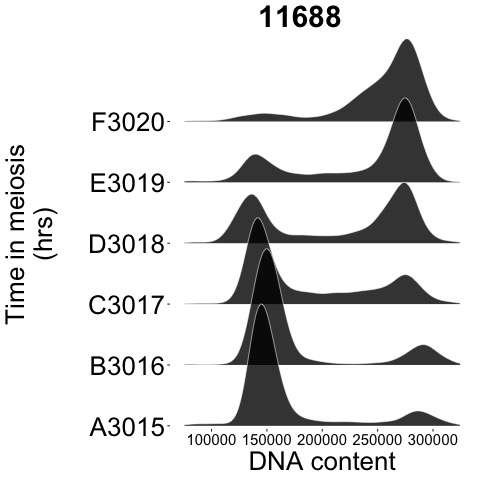

Supplement: Supplementary file 11 — Figure EV3 BC Source Data [file 44318_2024_34_MOESM11_ESM.zip › Data_Figure_EV3_BC/FACS/2023-08-29/11688.jpg]

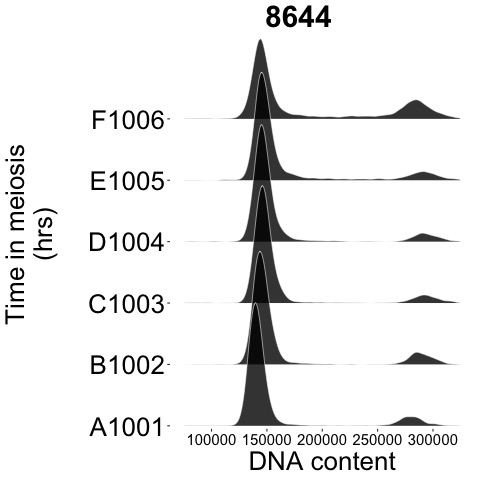

Supplement: Supplementary file 11 — Figure EV3 BC Source Data [file 44318_2024_34_MOESM11_ESM.zip › Data_Figure_EV3_BC/FACS/2023-08-29/8644.jpg]

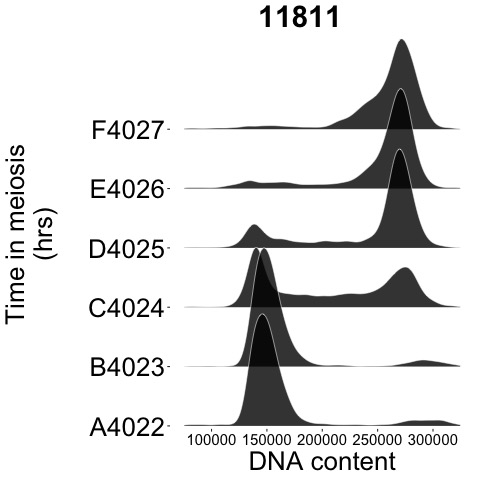

Supplement: Supplementary file 11 — Figure EV3 BC Source Data [file 44318_2024_34_MOESM11_ESM.zip › Data_Figure_EV3_BC/FACS/2023-08-29/11811.jpg]

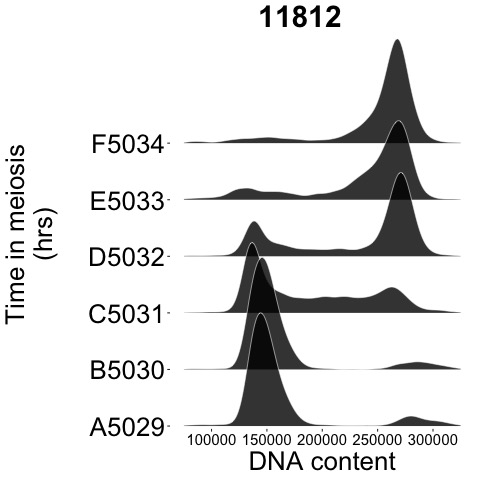

Supplement: Supplementary file 11 — Figure EV3 BC Source Data [file 44318_2024_34_MOESM11_ESM.zip › Data_Figure_EV3_BC/FACS/2023-08-29/11812.jpg]

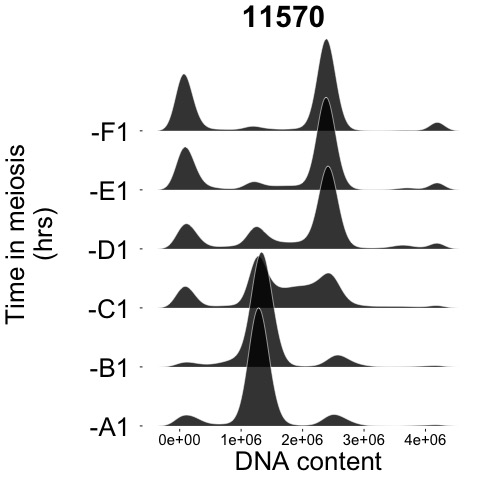

Supplement: Supplementary file 11 — Figure EV3 BC Source Data [file 44318_2024_34_MOESM11_ESM.zip › Data_Figure_EV3_BC/FACS/2023-08-13/Batch_A/11570.jpg]

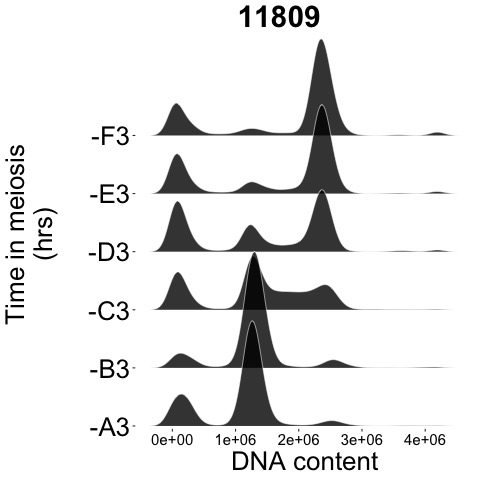

Supplement: Supplementary file 11 — Figure EV3 BC Source Data [file 44318_2024_34_MOESM11_ESM.zip › Data_Figure_EV3_BC/FACS/2023-08-13/Batch_A/11809.jpg]

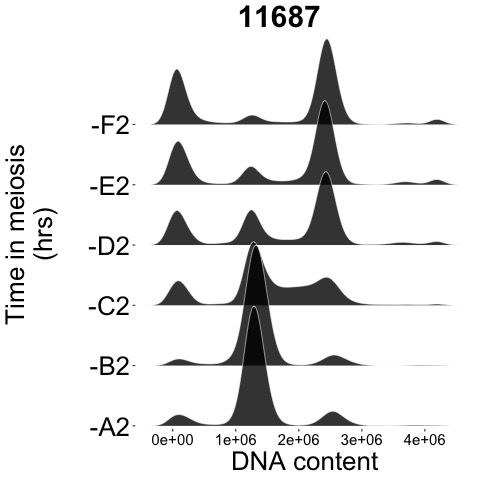

Supplement: Supplementary file 11 — Figure EV3 BC Source Data [file 44318_2024_34_MOESM11_ESM.zip › Data_Figure_EV3_BC/FACS/2023-08-13/Batch_A/11687.jpg]

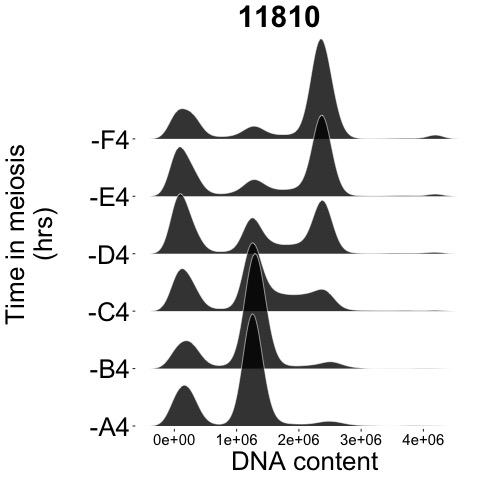

Supplement: Supplementary file 11 — Figure EV3 BC Source Data [file 44318_2024_34_MOESM11_ESM.zip › Data_Figure_EV3_BC/FACS/2023-08-13/Batch_A/11810.jpg]

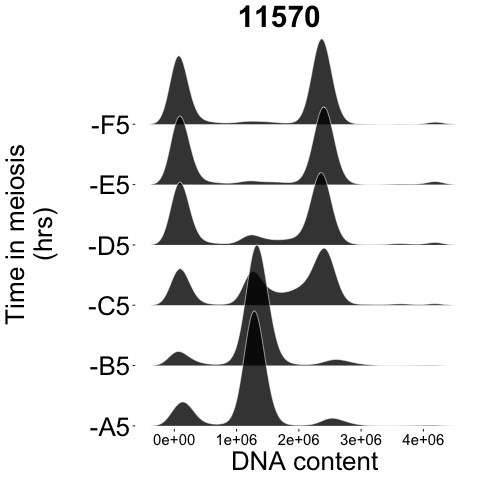

Supplement: Supplementary file 11 — Figure EV3 BC Source Data [file 44318_2024_34_MOESM11_ESM.zip › Data_Figure_EV3_BC/FACS/2023-08-13/Batch_B/11570.jpg]

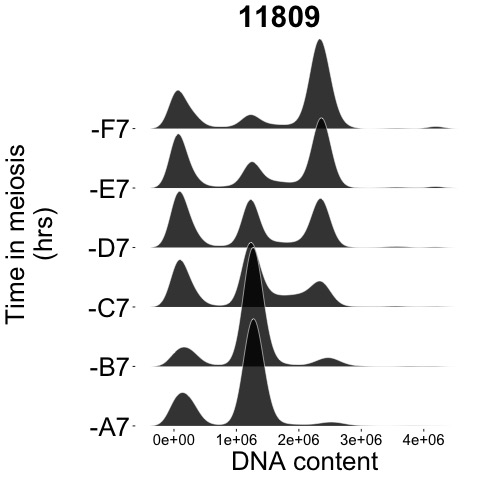

Supplement: Supplementary file 11 — Figure EV3 BC Source Data [file 44318_2024_34_MOESM11_ESM.zip › Data_Figure_EV3_BC/FACS/2023-08-13/Batch_B/11809.jpg]

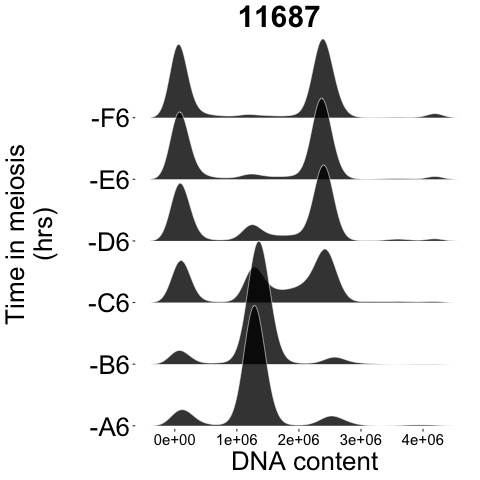

Supplement: Supplementary file 11 — Figure EV3 BC Source Data [file 44318_2024_34_MOESM11_ESM.zip › Data_Figure_EV3_BC/FACS/2023-08-13/Batch_B/11687.jpg]

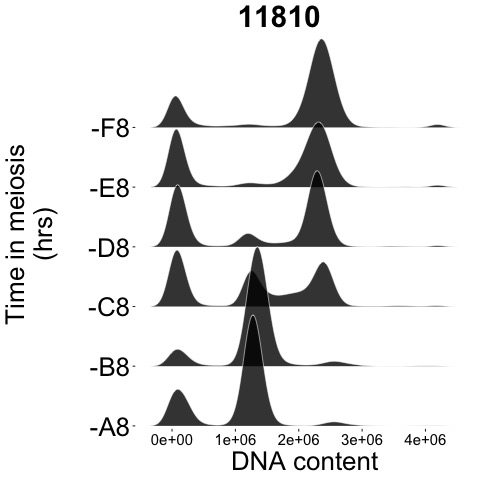

Supplement: Supplementary file 11 — Figure EV3 BC Source Data [file 44318_2024_34_MOESM11_ESM.zip › Data_Figure_EV3_BC/FACS/2023-08-13/Batch_B/11810.jpg]

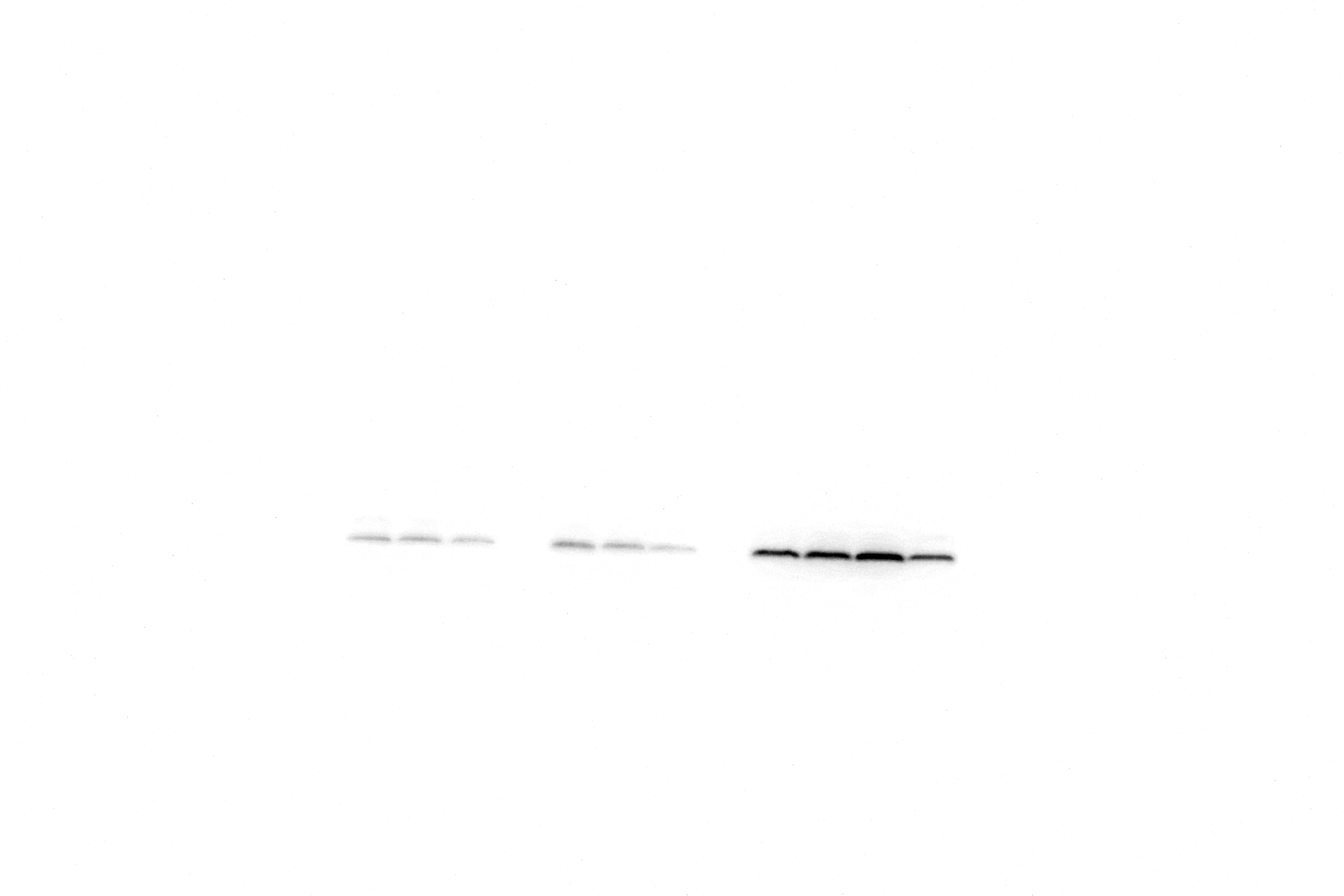

Supplement: Supplementary file 12 — Figure EV3 D Source Data [file 44318_2024_34_MOESM12_ESM.zip › Data_Figure_EV_D/DSCF0072_BW.jpg]

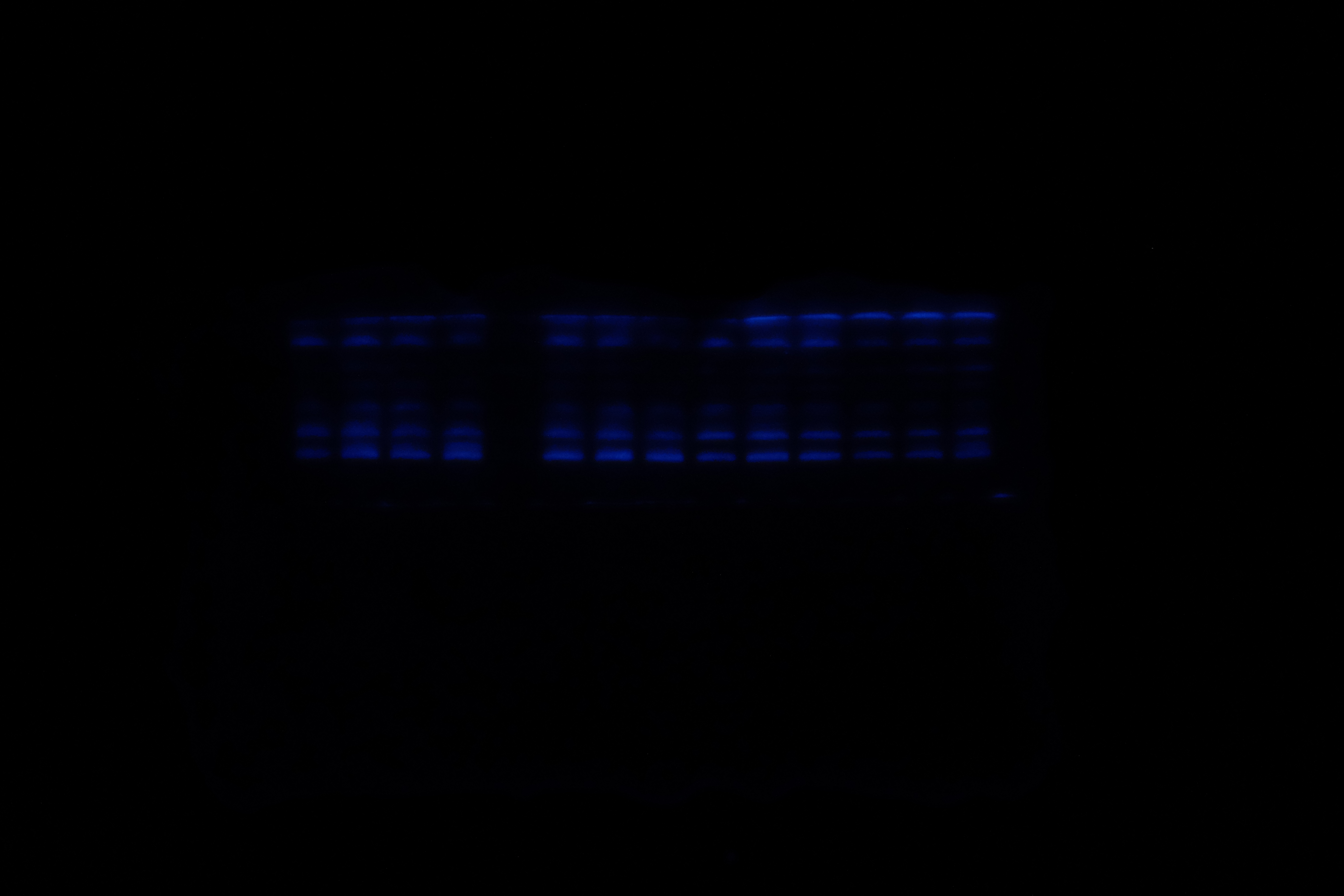

Supplement: Supplementary file 12 — Figure EV3 D Source Data [file 44318_2024_34_MOESM12_ESM.zip › Data_Figure_EV_D/DSCF0084.JPG]

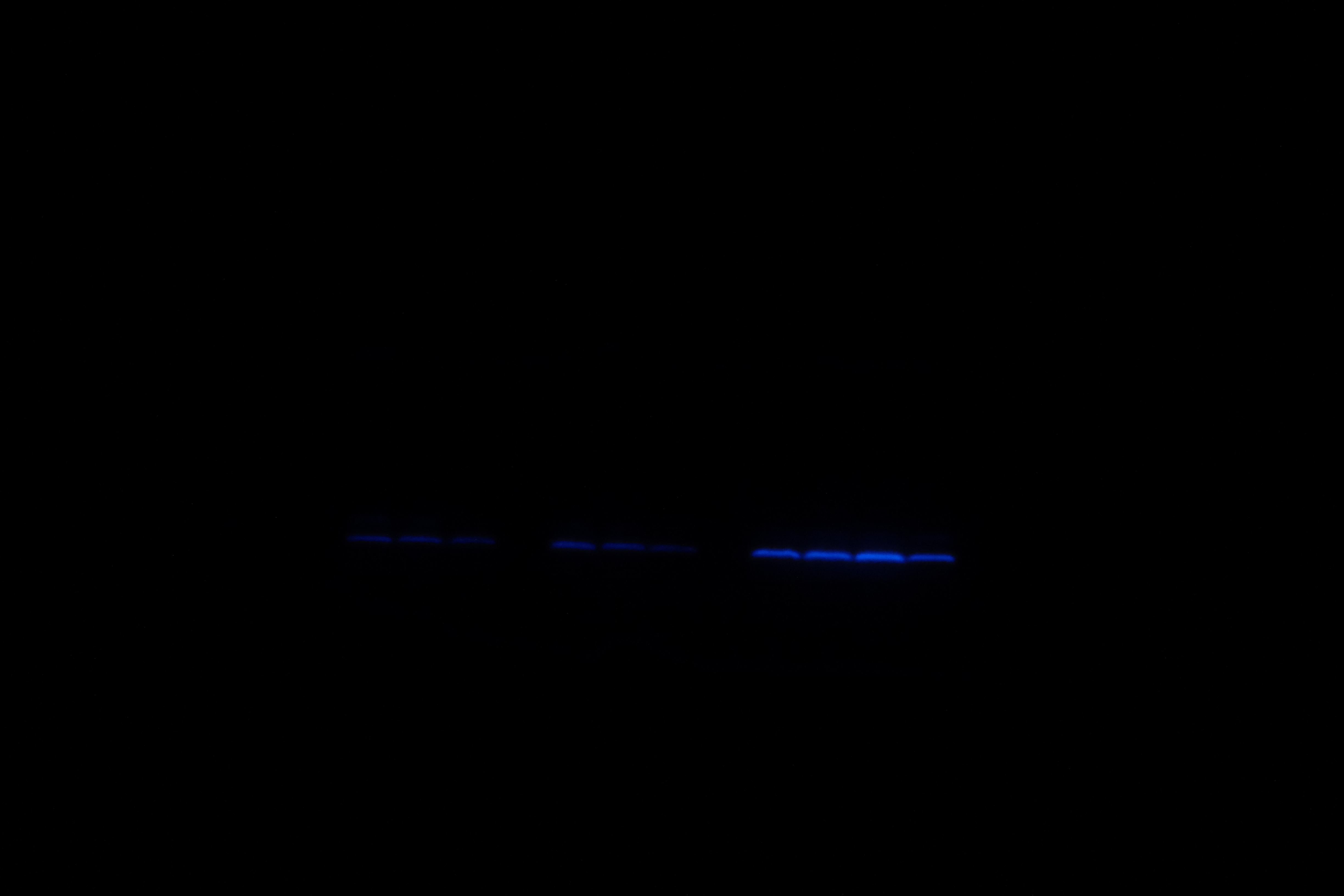

Supplement: Supplementary file 12 — Figure EV3 D Source Data [file 44318_2024_34_MOESM12_ESM.zip › Data_Figure_EV_D/DSCF0072.JPG]

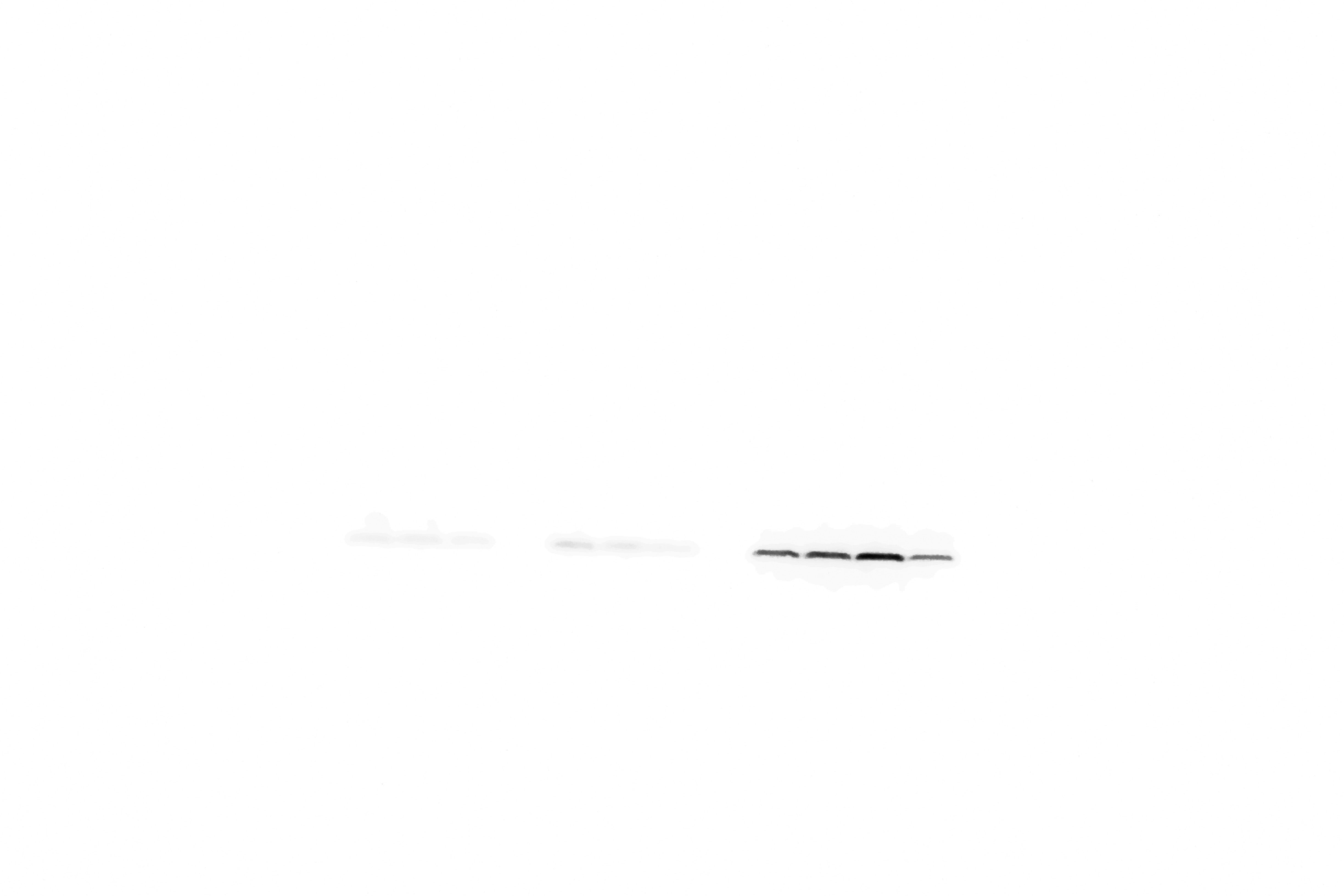

Supplement: Supplementary file 12 — Figure EV3 D Source Data [file 44318_2024_34_MOESM12_ESM.zip › Data_Figure_EV_D/2022-10-20_11644-11757_DSCF0071.jpg_BW.jpg]

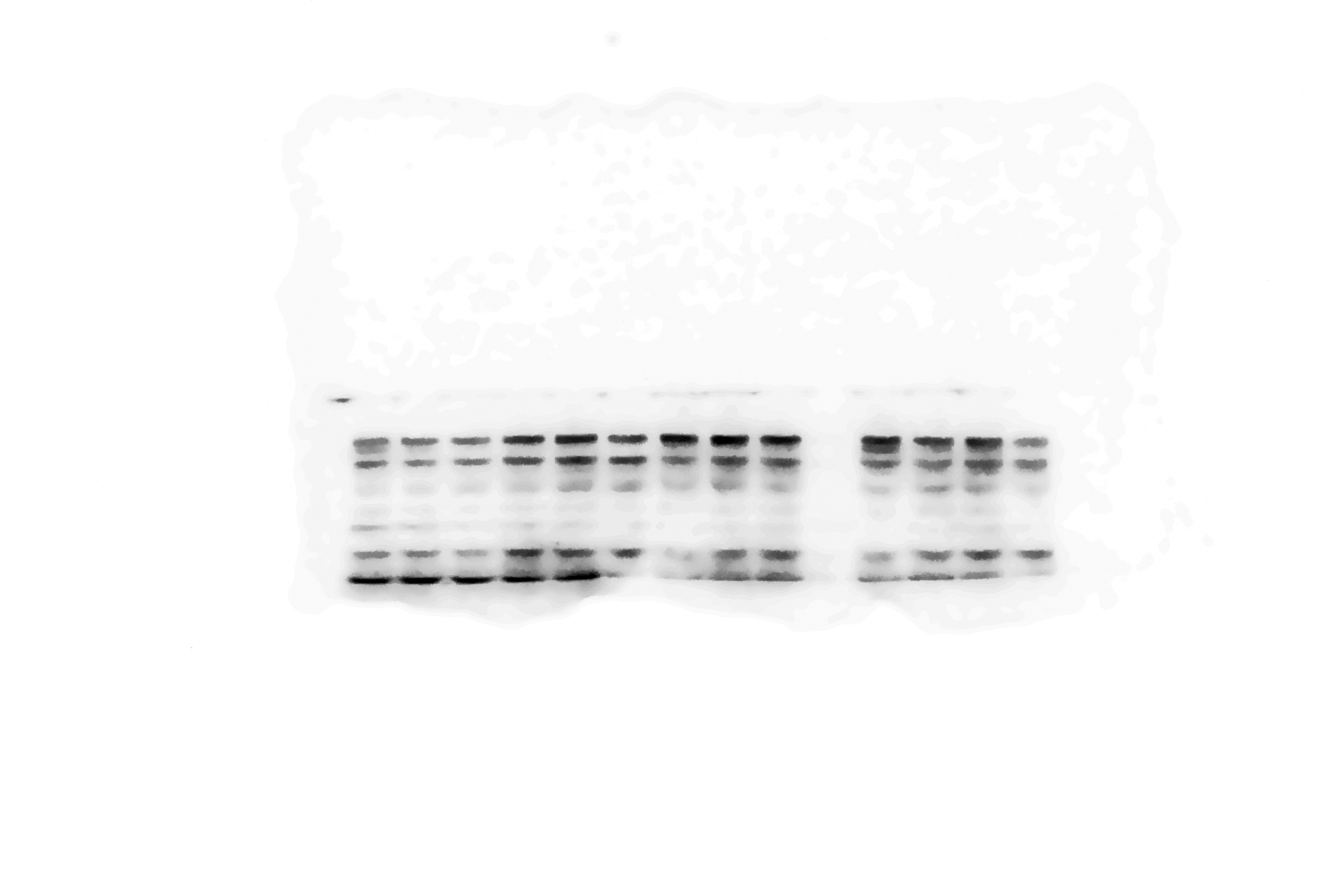

Supplement: Supplementary file 12 — Figure EV3 D Source Data [file 44318_2024_34_MOESM12_ESM.zip › Data_Figure_EV_D/DSCF0084_BW.jpg]

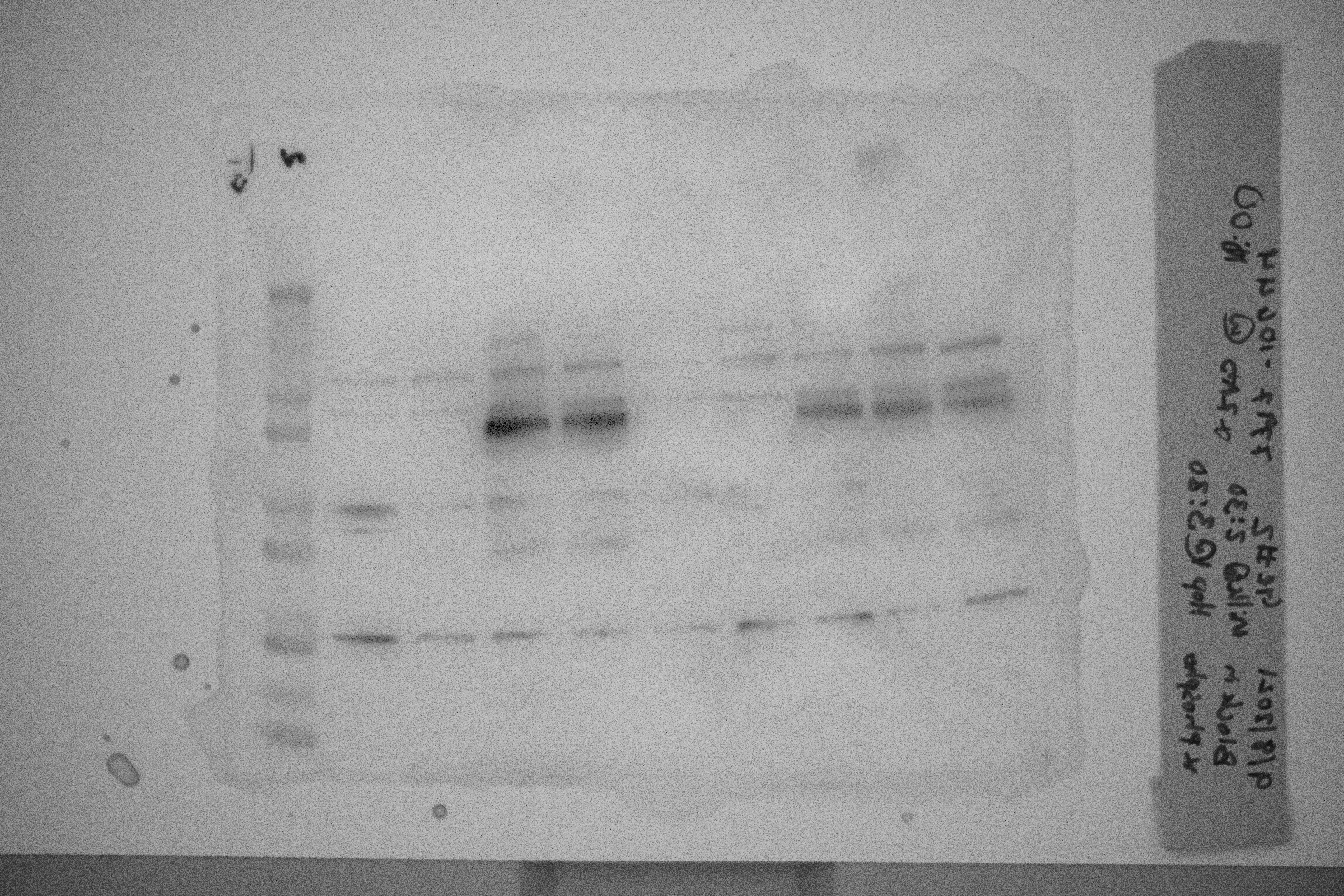

Supplement: Supplementary file 12 — Figure EV3 D Source Data [file 44318_2024_34_MOESM12_ESM.zip › Data_Figure_EV_D/mergedDSCF0069.png]

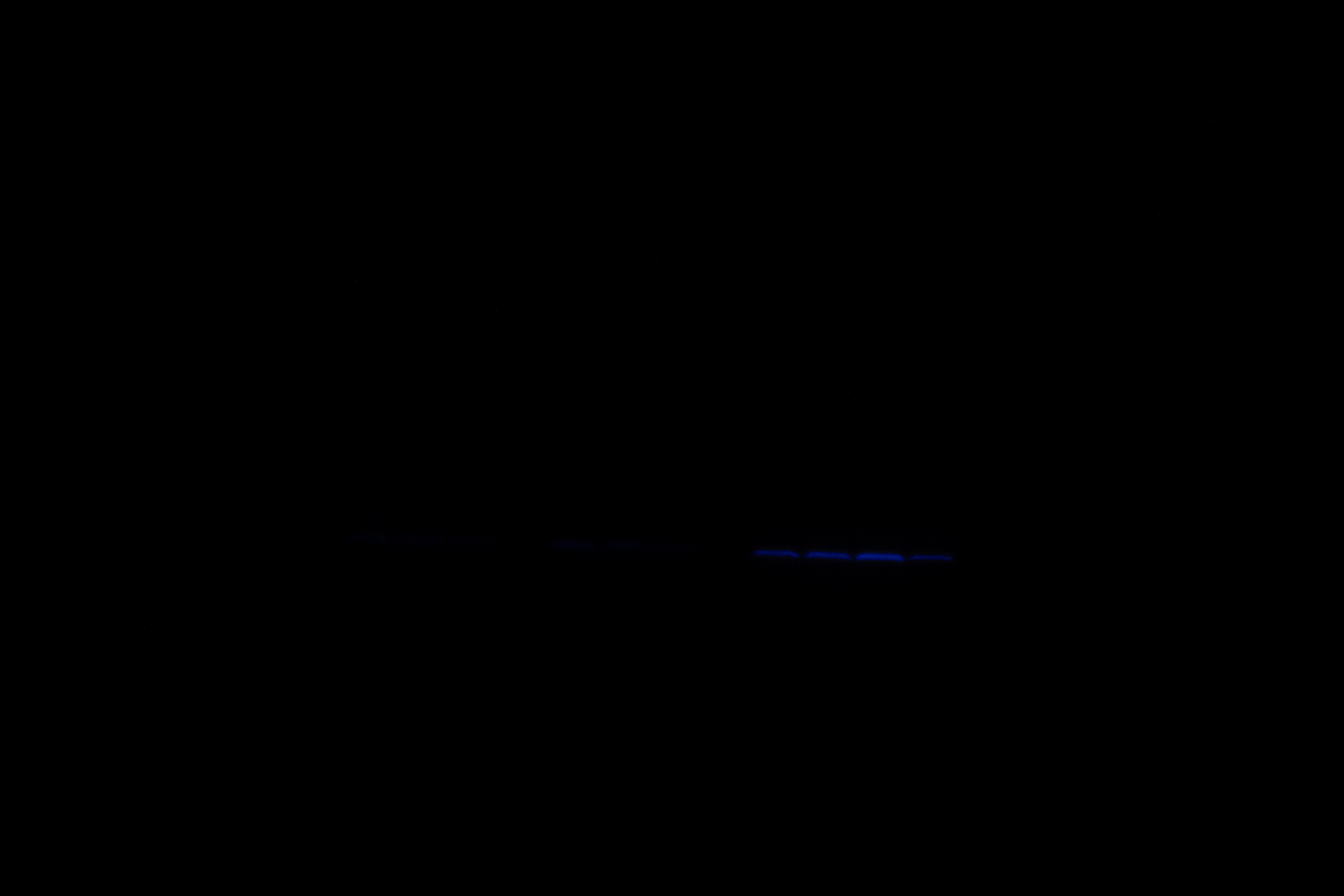

Supplement: Supplementary file 12 — Figure EV3 D Source Data [file 44318_2024_34_MOESM12_ESM.zip › Data_Figure_EV_D/2022-10-20_11644-11757_DSCF0071.JPG]
