# Supplementary material for: Chromatin binding by HORMAD proteins regulates meiotic recombination initiation
Source: EMBO J. 2024 Feb 8;43(5):8. doi: 10.1038/s44318-024-00034-3 (PMC10907721; doi:10.1038/s44318-024-00034-3)
Supplement: Supplementary file 17 — Expanded View Figures [file 44318_2024_34_MOESM17_ESM.pdf]

Expanded View Figures

Figure EV1. Structure of a nucleosome + 2 Hop1 CBR complex.

(**A**) Three views of cryo-EM density for a nucleosome + 2 Hop1 CBR complex (3.15 Å overall resolution, gaussian-smoothed with a σ of 1.1 Å). DNA is colored gray, histones colored yellow (H2A), red (H2B), blue (H3), and green (H4), and two Hop1 CBR domains colored yellow and orange. (**B**) Molecular model for a nucleosome + 2 Hop1 CBR complex, colored as in panel (**A**), except Hop1 CBR is colored blue (PHD), green (wHTH), and orange (HTH-C). (**C**) Schematic of the Widom 601 DNA sequence used for nucleosome assembly, with binding sites for Hop1 CBR #1 (blue) and #2 (green) noted. SHL: superhelical locations, 0 at the nucleosome dyad axis to 7 at the DNA ends. (**D**) Structural overlay of the DNA bound to Hop1 CBR #1 (blue) and #2 (green). In sequence alignment at bottom, solid lines indicate identity and dotted lines indicate shared status as either pyrimidine or purine.

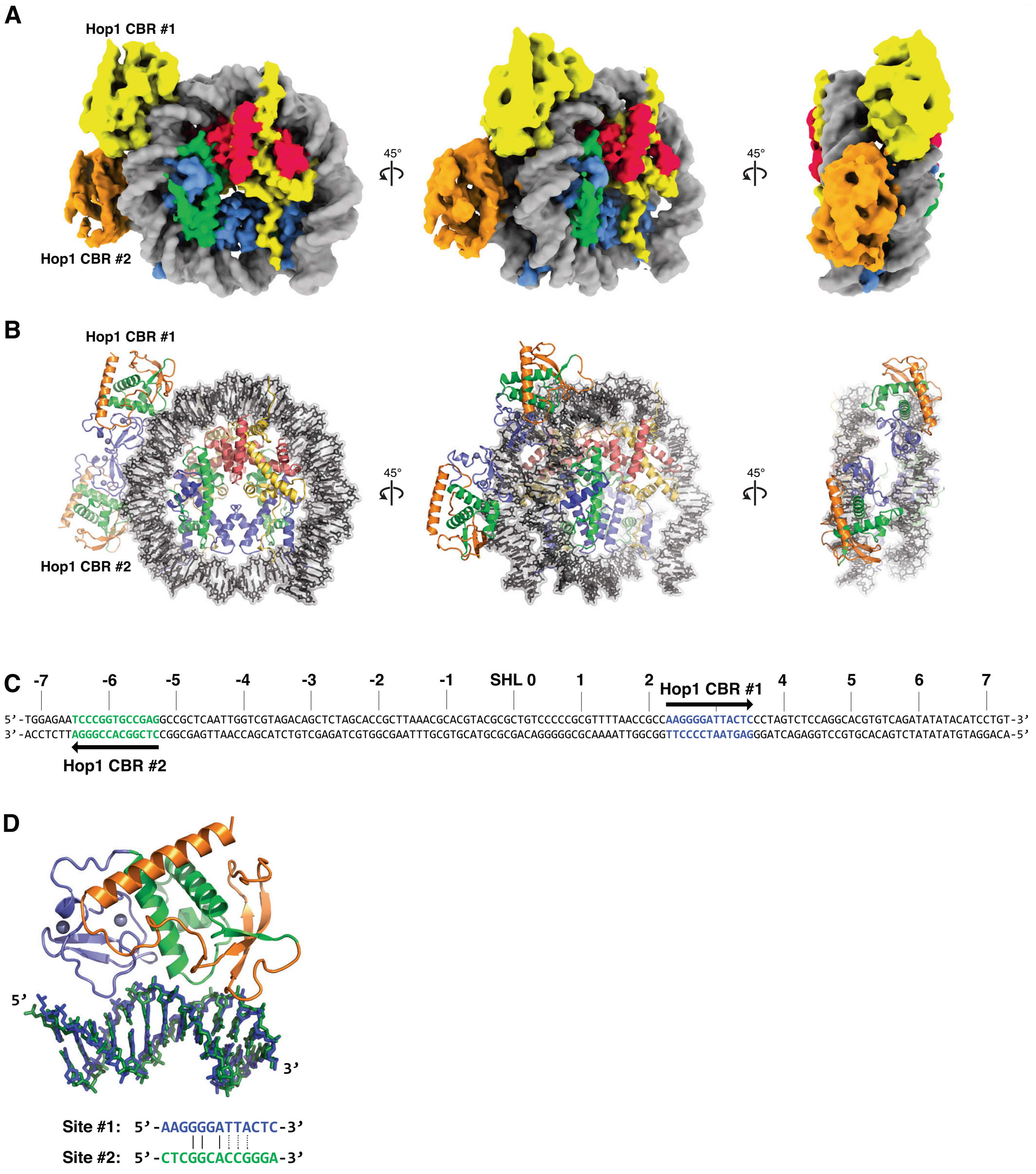
A
Hop1 CBR #1
Hop1 CBR #2
45°
45°
B
Hop1 CBR #1
Hop1 CBR #2
45°
45°
C
-7 -6 -5 -4 -3 -2 -1 SHL 0 1 2 4 5 6 7
Hop1 CBR #1
5’-TGGAGAATCCCGGTGCCGAGGCCGCTCAATTGGTCGTAGACAGCTCTAGCACCGCTTAAACGCACGTACGCGCTGTCCCCCGCGTTTTAACCGCCAAGGGGATTACTCCCTAGTCTCCAGGCACGTGTCAGATATATACATCCTGT-3’
3’-ACCTCTTAGGGCCACGGCTCCGGCGAGTTAACCAGCATCTGTCGAGATCGTGGCGAATTTGCGTGCATGCGCGACAGGGGGCGCAAAATTGGCGGTTCCCCTAATGAGGGATCAGAGGTCCGTGCACAGTCTATATATGTAGGACA-5’
Hop1 CBR #2
D
5’
3’
Site #1: 5’-AAGGGGATTACTC-3’
Site #2: 5’-CTCGGCACCGGGA-3’

 © The Author(s)

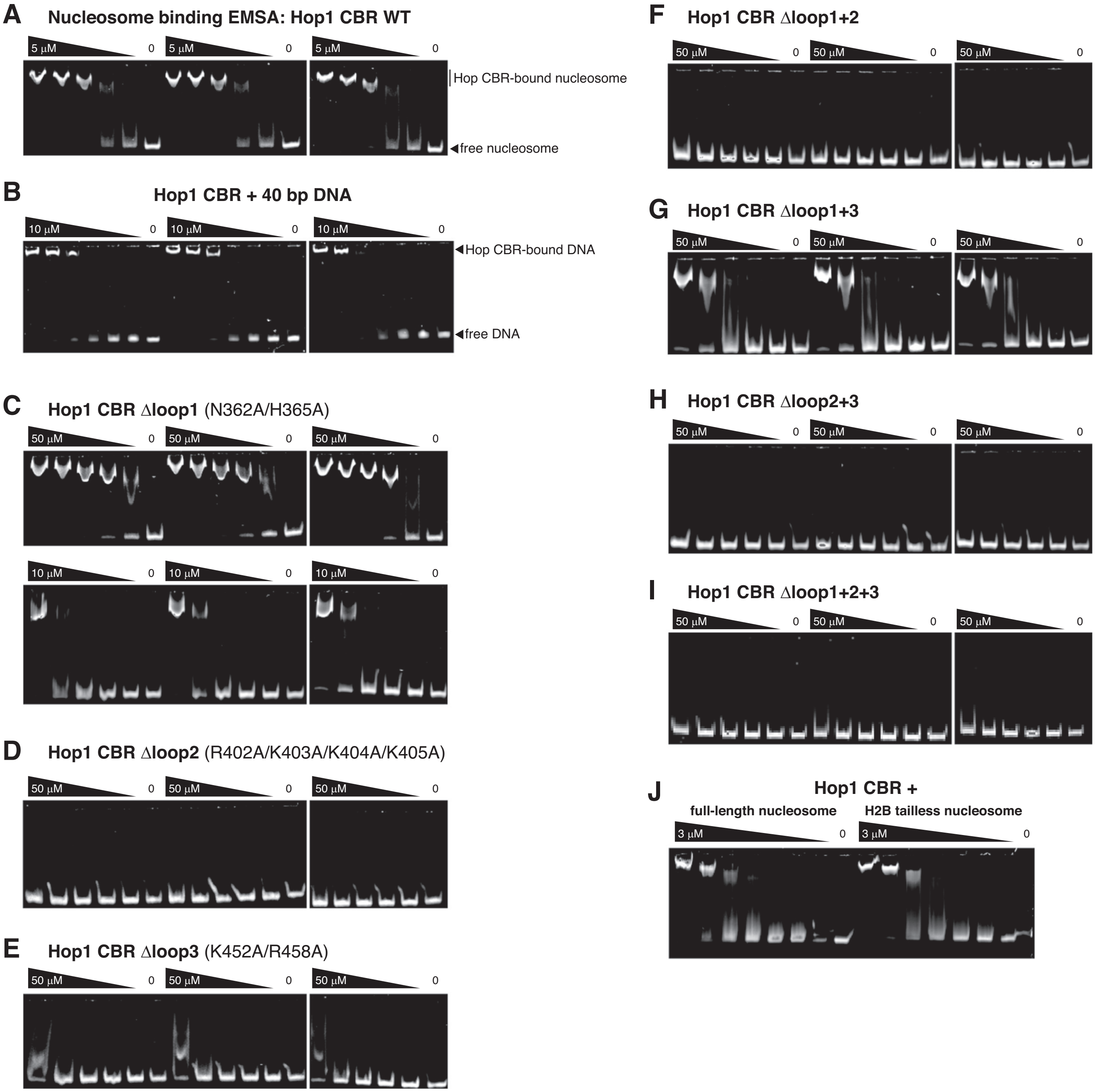

Figure EV2. Nucleosome and DNA binding by the *S. cerevisiae* Hop1 CBR.

(**A**) Triplicate electrophoretic mobility shift assays (EMSAs) for wild-type Hop1 CBR binding to reconstituted nucleosomes. The highest concentration of Hop1 CBR used was 5 μM, followed by serial 2× dilutions. Band intensities were quantified in ImageJ and K_d was calculated in Prism using a single-site binding model ($K_d = 0.47 +/- 0.11$ μM). The representative gel image shown in Fig. 1F also appears here. (**B**) Triplicate EMSAs for wild-type Hop1 CBR binding a 40-bp DNA encompassing its preferred binding site on the Widom 601 DNA ($K_d = 2.1 +/- 0.6$ μM). (**C**) Triplicate EMSAs for Hop1 CBR Δloop 1 mutant (N362A/H365A) binding nucleosomes, with two different starting protein concentrations (top: 50 μM; bottom: 10 μM) ($K_d = 2.0 +/- 0.4$ μM). (**D**) Triplicate EMSAs for Hop1 CBR Δloop2 mutant (R402A/K403A/K404A/K405A) binding nucleosomes ($K_d > 50$ μM). The representative gel image shown in Fig. 1F also appears here. (**E**) Triplicate EMSAs for Hop1 CBR Δloop 3 mutant (K452A/R456A) binding nucleosomes ($K_d \sim 50$ μM). (**F**) Triplicate EMSAs for Hop1 CBR Δloop 1 + 2 mutant binding nucleosomes ($K_d > 50$ μM). (**G**) Triplicate EMSAs for Hop1 CBR Δloop 1 + 3 mutant binding nucleosomes ($K_d = 13 +/- 3$ μM). (**H**) Triplicate EMSAs for Hop1 CBR Δloop2 + 3 mutant binding nucleosomes ($K_d > 50$ μM). (**I**) Triplicate EMSAs for Hop1 CBR Δloop 1 + 2 + 3 mutant binding nucleosomes ($K_d > 50$ μM). The representative gel image shown in Fig. 1F also appears here. (**J**) EMSAs comparing binding of wild-type Hop1 CBR to nucleosomes reconstituted with full-length (left) or tailless (right) histone H2B.

© The Author(s)

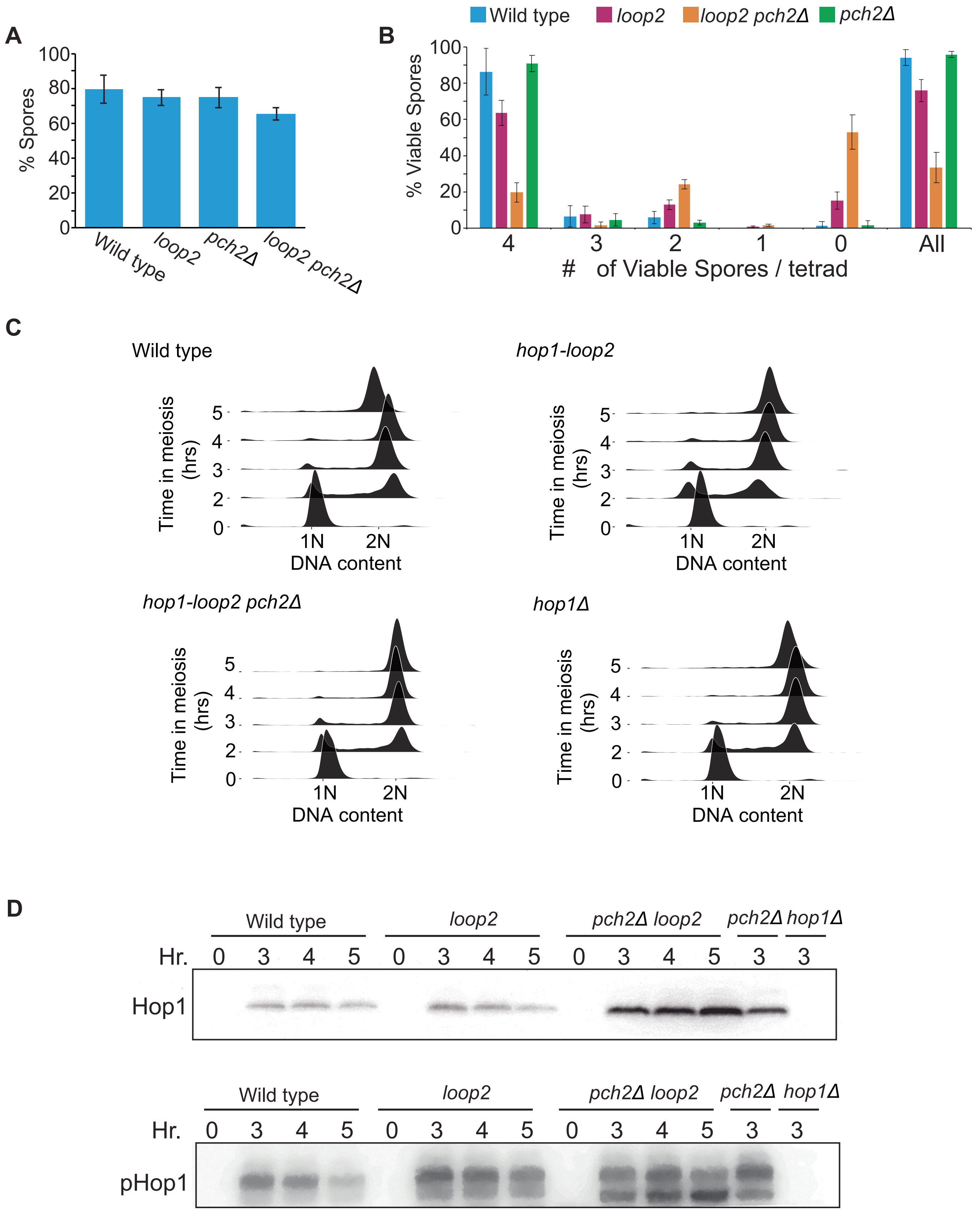
A
% Spores
100
80
60
40
20
0
Wild type
loop2
pch2Δ
loop2 pch2Δ
B
Wild type
loop2
loop2 pch2Δ
pch2Δ
% Viable Spores
100
80
60
40
20
0
4
3
2
1
0
All
# of Viable Spores / tetrad
C
Wild type
hop1-loop2
hop1-loop2 pch2Δ
hop1Δ
Time in meiosis (hrs)
5
4
3
2
0
1N
2N
DNA content
D
Wild type
loop2
pch2Δ loop2
pch2Δ
hop1Δ
Hr.
0 3 4 5 0 3 4 5 0 3 4 5 3 3
Hop1
pHop1

 © The Author(s)

◀ **Figure EV3. Hop1 protein and phosphorylation levels across genotypes.**

The *hop1-loop2* allele is simplified to *loop2* in figure labels. (**A**) Sporulation efficiency data for the indicated genotypes. This data represents at least four biological replicates. (**B**) Number of viable spores per tetrad for each genotype. At least 50 tetrads were dissected from each genotype. (**C**) Flow cytometry analysis of DNA content, monitoring DNA synthesis to indicate synchronous meiotic entry. This was performed every time samples were prepared from a meiotic yeast culture. (**D**) Western blots showing protein levels across genotypes for Hop1 protein (Top) (Subramanian et al, 2016), and for phosphorylated Hop1 T318 (Subramanian et al, 2016). This result was observed with at least three biological replicates. Source data are available online for this figure.

© The Author(s)

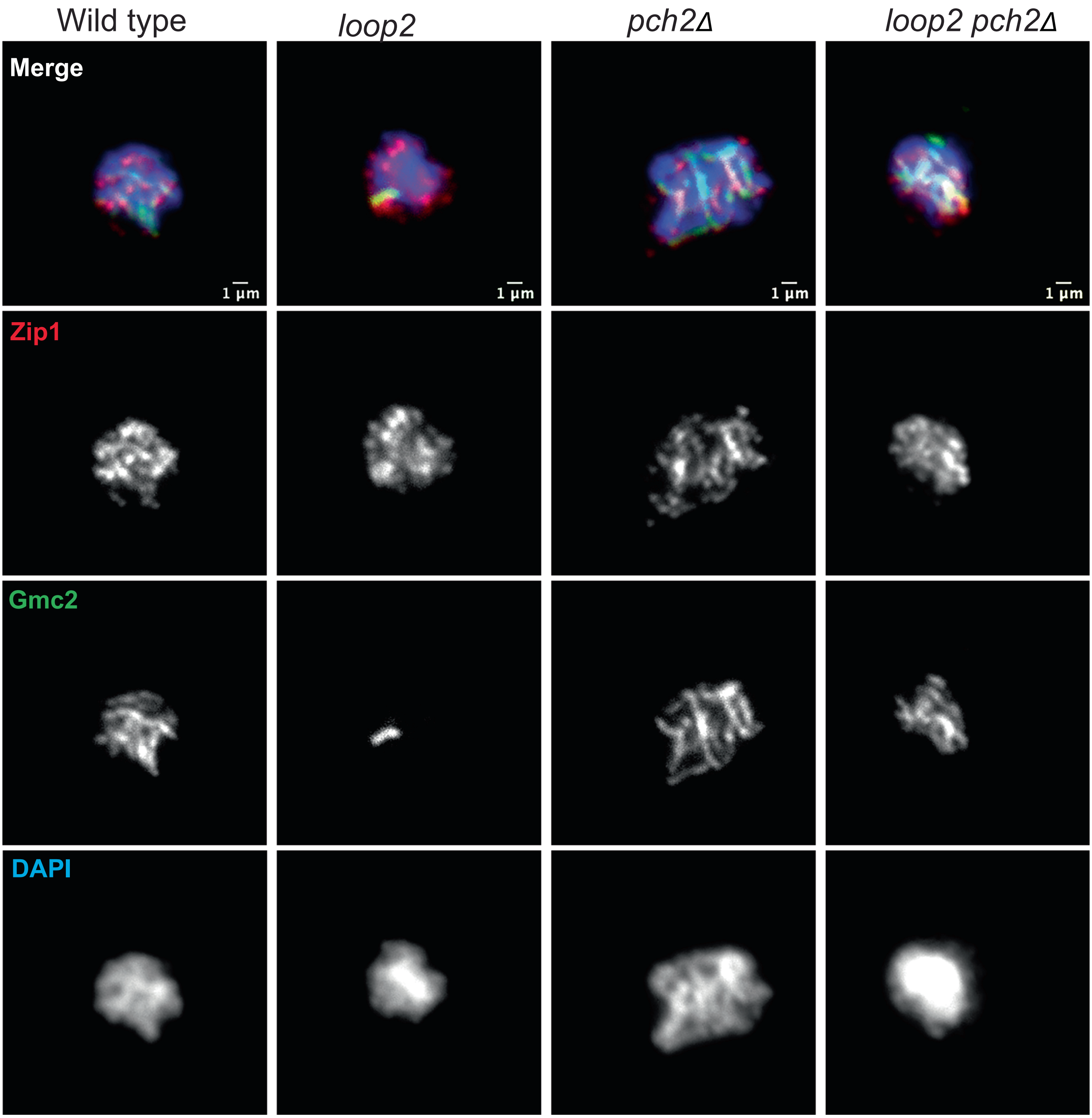

Figure EV4. Localization of the central-element protein Gmc2.

The *hop1-loop2* allele is simplified to *loop2* in figure labels. Samples were taken from meiotic cultures of the indicated genotypes at hour 3, chromosome spreads were prepared, hybridized with antibodies against Zip1 (pink) and Gmc2 (green), and stained with DAPI (blue). This was generated from samples taken from one meiotic time course. Source data are available online for this figure.

 © The Author(s)

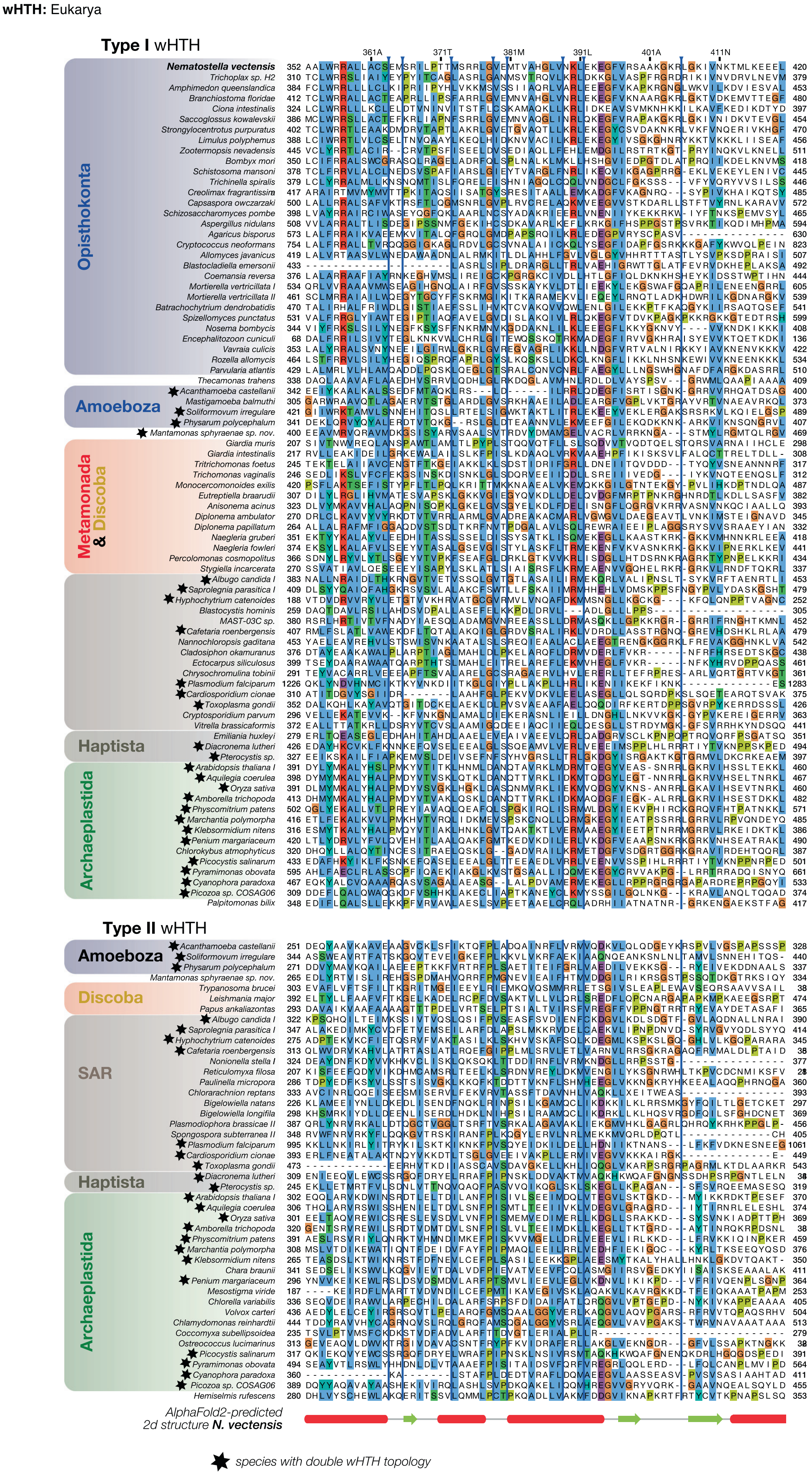
wHTH: Eukarya
Type I wHTH
361A
371T
381M
391L
401A
411N
Opisthokonta
Amoeboza
Metamonada & Discoba
Haptista
Archaeplastida
Type II wHTH
Amoeboza
Discoba
SAR
Haptista
Archaeplastida
AlphaFold2-predicted 2d structure N. vectensis
species with double wHTH topology

Figure EV5. Meiotic HORMAD wHTH domains can be classified into two types of which one of each type is present in meiotic HORMADs with a tandem wHTH configuration.

Sequence alignment of wHTH domains found in 75 single wHTH and 25 double wHTH among 105 different eukaryotic species (Table EV1)—excluding those of the Saccharomycetaceae. To make the alignment more easily readable, we used the wHTH domain of *Nematostella vectensis* (Uniprot ID A7RLI6) as a reference and excluded any column in the multiple alignment that was not found in the sequence of this domain (see blue lines for marks of column removal). Full multiple alignments can be found in File S1 on https://github.com/hochwagenlab/Hop1-loop2. wHTH domains were manually classified into two types that correspond to either one of the wHTH domains found in meiotic HORMADs with two wHTH domains (see star). Phylogenetic tree analyses did not yield statistically sensible trees, likely due to the rather high sequence divergence found among the wHTH domains in these meiotic HORMAD proteins. Type 1 wHTHs are characterized by a conserved positively charged patch in the first alpha helix, while Type 2 wHTHs harbor a conserved 'FP' motif in a loop between two central alpha helices (see for classification Table EV1). Note that the wHTH domain is conserved until the first beta strand of the 'wing'. Most wHTHs in other meiotic HORMADs found among eukaryotes do have a second strand and a capping helix, but this part of the domain is highly divergent between lineages (i.e., where loop 3 in Saccharomycetaceae resides).

 © The Author(s)
