# Supplementary material for: Chromatin binding by HORMAD proteins regulates meiotic recombination initiation
Source: EMBO J. 2024 Feb 8;43(5):8. doi: 10.1038/s44318-024-00034-3 (PMC10907721; doi:10.1038/s44318-024-00034-3)
Supplement: Supplementary file 6 — Source Data Fig. 5 [file 44318_2024_34_MOESM6_ESM.zip › Data_Figure_5/Zip1TC_loop2/results/graphs_pdf/Average Zip1 Branch Length.pdf]

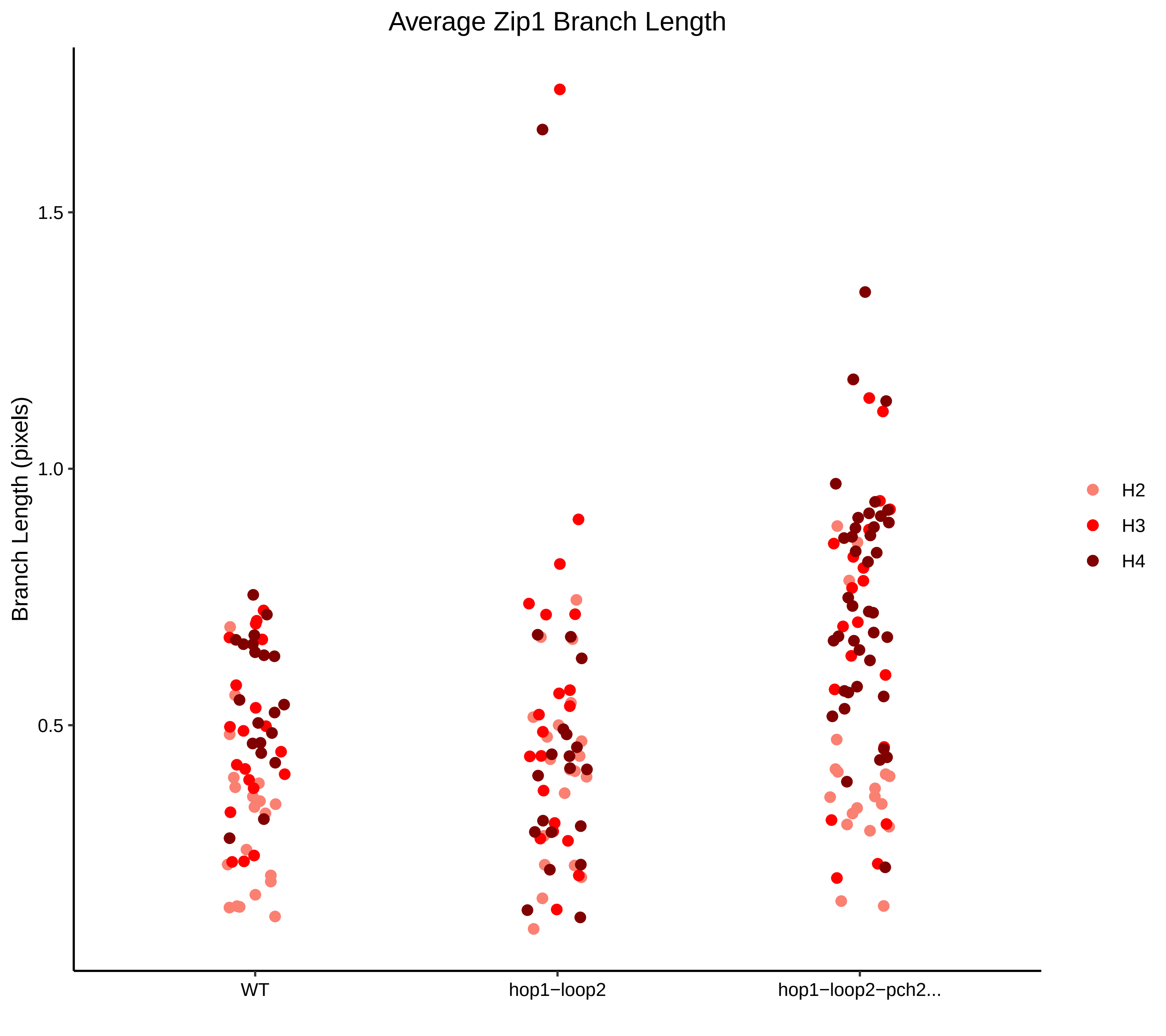
Average Zip1 Branch Length
Branch Length (pixels)
1.5
1.0
0.5
WT
hop1−loop2
hop1−loop2−pch2...
H2
H3
H4
