# Supplementary material for: Chromatin binding by HORMAD proteins regulates meiotic recombination initiation
Source: EMBO J. 2024 Feb 8;43(5):8. doi: 10.1038/s44318-024-00034-3 (PMC10907721; doi:10.1038/s44318-024-00034-3)
Supplement: Supplementary file 6 — Source Data Fig. 5 [file 44318_2024_34_MOESM6_ESM.zip › Data_Figure_5/2022-11-17_11758_tc/results/graphs/pch2delta alone/Hop1 Intensity - pch2 only.pdf]

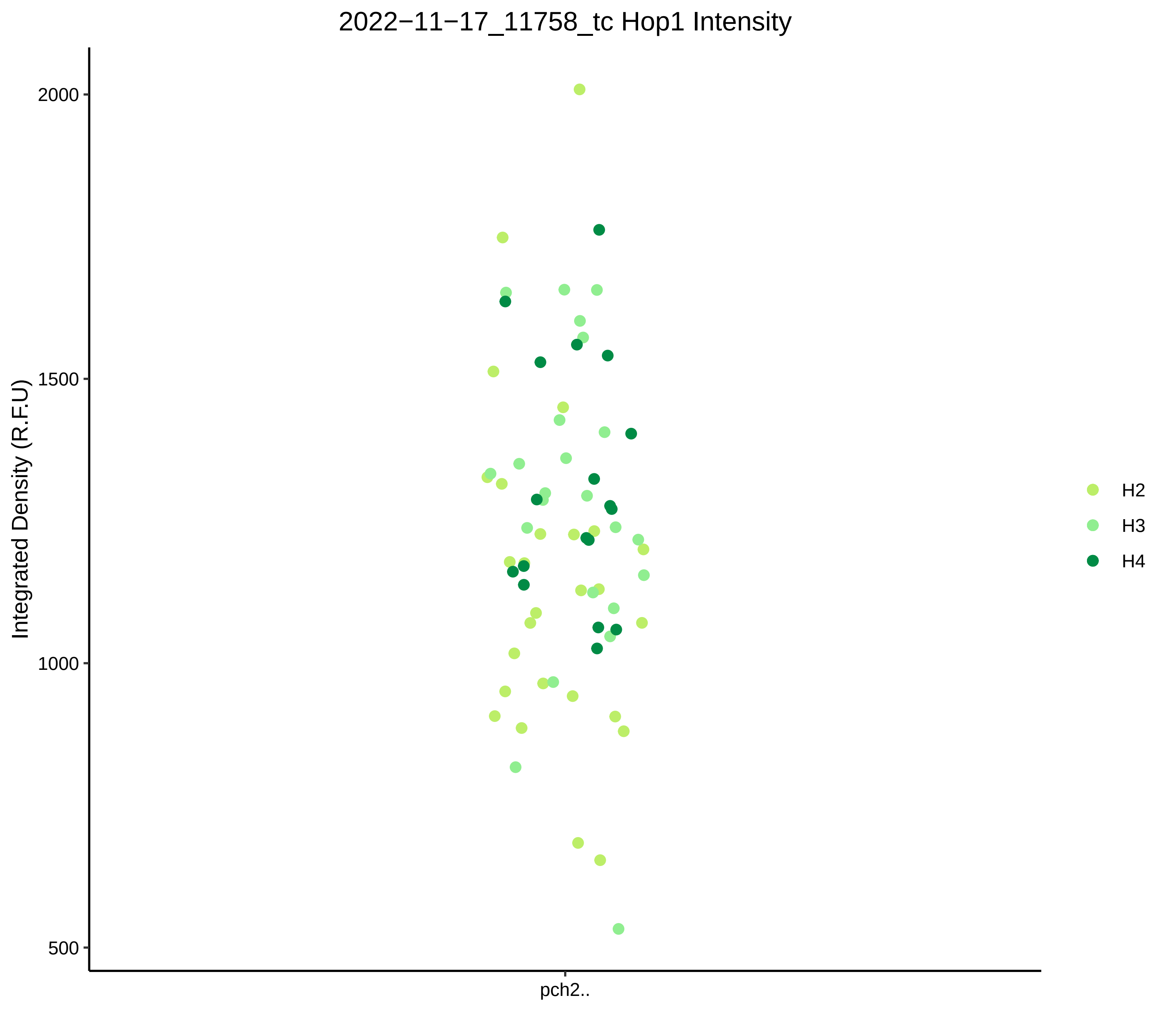
2022−11−17_11758_tc Hop1 Intensity
Integrated Density (R.F.U)
2000
1500
1000
500
pch2..
H2
H3
H4
