# Supplementary material for: Chromatin binding by HORMAD proteins regulates meiotic recombination initiation
Source: EMBO J. 2024 Feb 8;43(5):8. doi: 10.1038/s44318-024-00034-3 (PMC10907721; doi:10.1038/s44318-024-00034-3)
Supplement: Supplementary file 7 — Source Data Fig. 6 [file 44318_2024_34_MOESM7_ESM.zip › Data_Figure_6/GAT1_PstI-RE/HIS4LEU2-1D-DSB_nolegend.pdf]

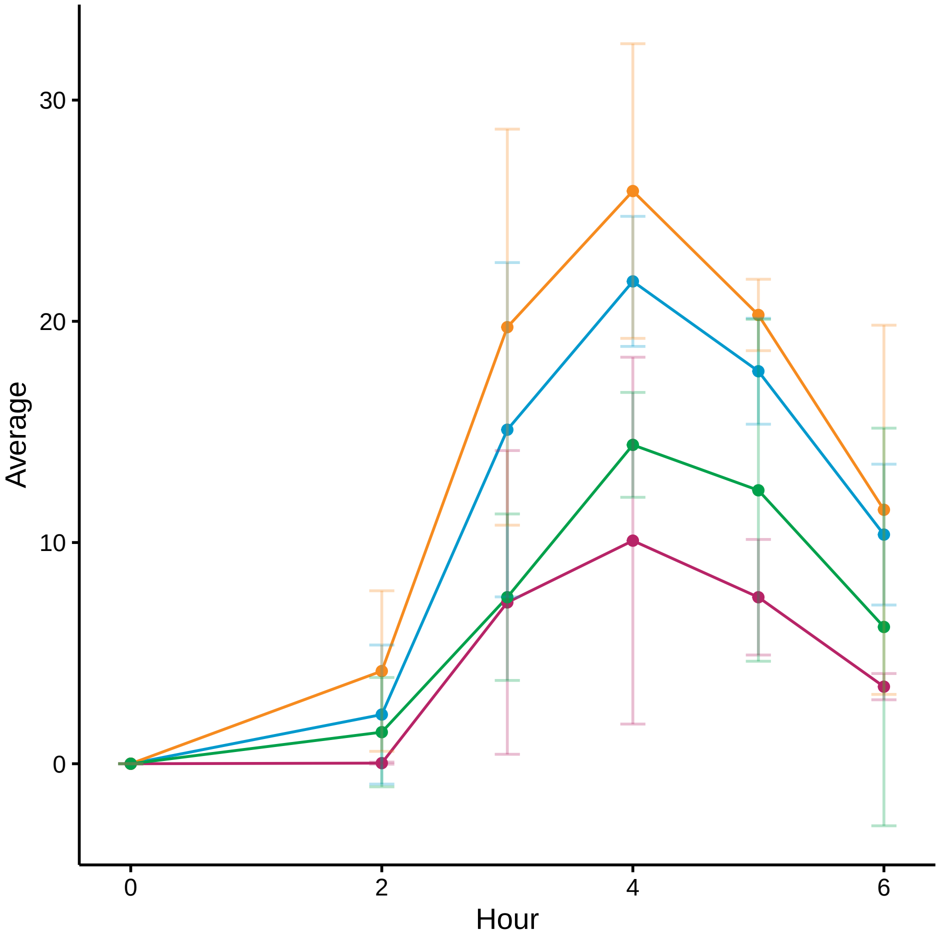

Average
30
20
10
0
0
2
4
6
Hour
