# Supplementary material for: Chromatin binding by HORMAD proteins regulates meiotic recombination initiation
Source: EMBO J. 2024 Feb 8;43(5):8. doi: 10.1038/s44318-024-00034-3 (PMC10907721; doi:10.1038/s44318-024-00034-3)
Supplement: Supplementary file 7 — Source Data Fig. 6 [file 44318_2024_34_MOESM7_ESM.zip › Data_Figure_6/GAT1_PstI-RE/Gat1_all.pdf]

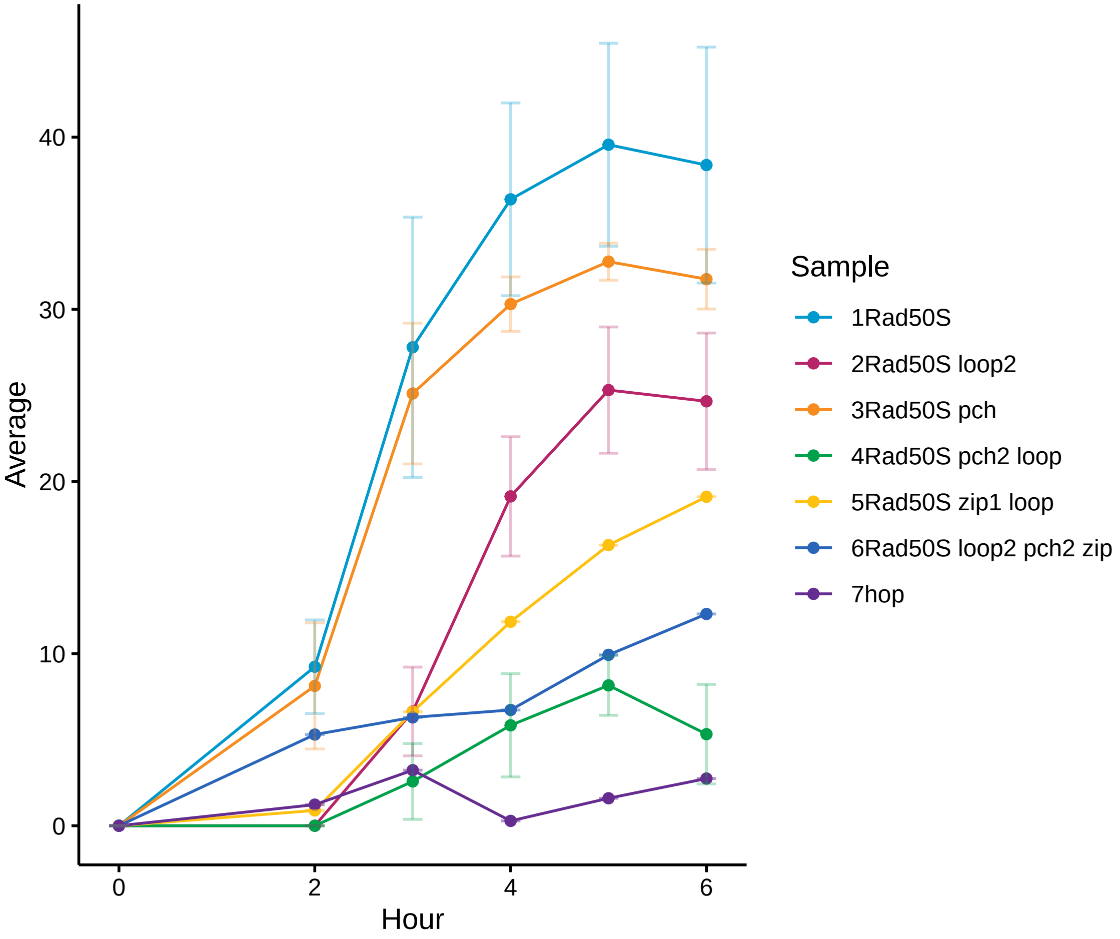

Average
40
30
20
10
0
0
2
4
6
Hour
Sample
1Rad50S
2Rad50S loop2
3Rad50S pch
4Rad50S pch2 loop
5Rad50S zip1 loop
6Rad50S loop2 pch2 zip
7hop
