# Supplementary material for: Chromatin binding by HORMAD proteins regulates meiotic recombination initiation
Source: EMBO J. 2024 Feb 8;43(5):8. doi: 10.1038/s44318-024-00034-3 (PMC10907721; doi:10.1038/s44318-024-00034-3)
Supplement: Supplementary file 15 — Appendix Figure 9 Source Data [file 44318_2024_34_MOESM15_ESM.zip › Data_AppendixFigure9/Chr16_Quant_images/Sam3_all_nolegend.pdf]

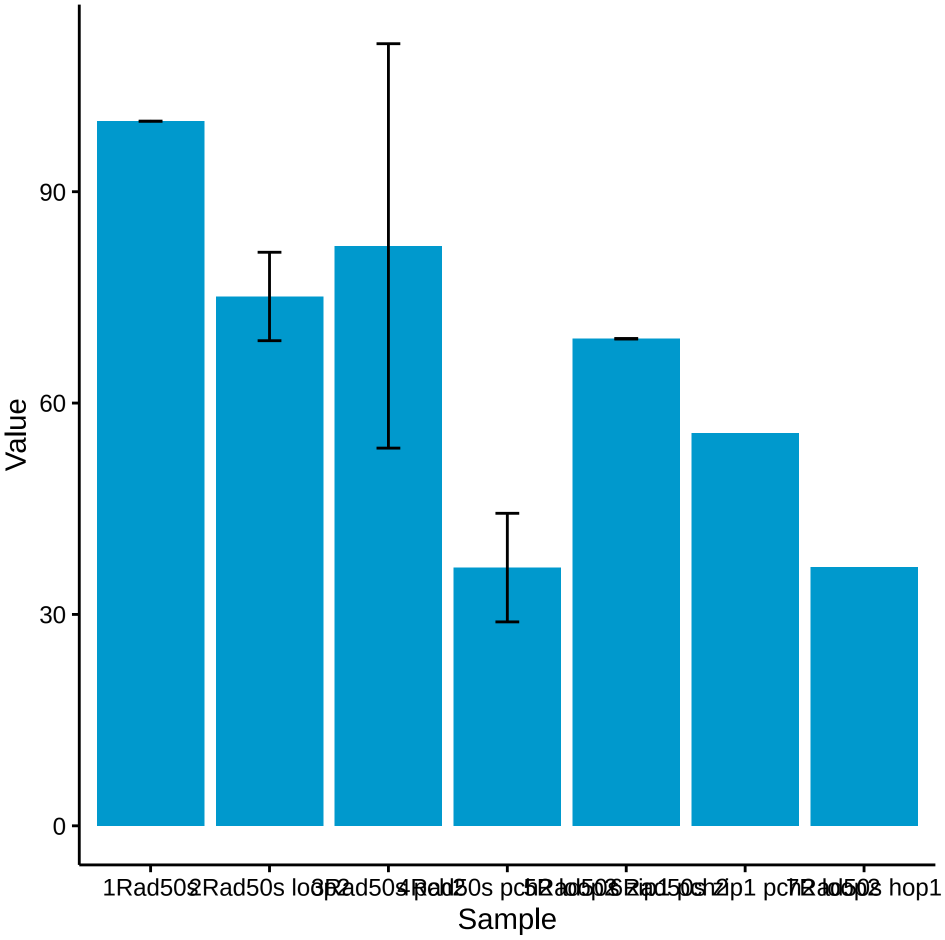
Value
90
60
30
0
Sample
